# Biosafety status analysis and risk assessment of laboratories from 2021 to 2023 in Jiaxing, China

**DOI:** 10.3389/fbioe.2025.1442651

**Published:** 2025-04-16

**Authors:** Yong Yan, Chan Li, Jingyu Xu, Lei Gao, Zhenggang Jiang, Shencong Lv, Ping Li, Zelin Xiang, Peiyan He, Ganglin Ren, Guoying Zhu, Zhongwen Chen

**Affiliations:** ^1^ Jiaxing Key Laboratory of Pathogenic Microbiology, Jiaxing Center for Disease Control and Prevention, Jiaxing, Zhejiang, China; ^2^ Department of Research and Emergency, Zhejiang Provincial Center for Disease Control and Prevention, Hangzhou, Zhejiang, China; ^3^ Department of Microbiology, Haiyan Center for Disease Control and Prevention, Haiyan, Zhejiang, China

**Keywords:** biosafety, biosecurity, risk assessment, laboratory, quality control, public health

## Abstract

In order to understand the laboratory biosafety status of Jiaxing from 2021 to 2023, before and after the end of the COVID-19 strict control strategy, this study used Zhejiang local standard DB33/T 2540 to conduct biosafety quality control inspection and risk identification, and used Chinese industry standard RB/T 040 for risk analysis and assessment. The results showed that the major problems in biosafety management were from organization management, laboratory housekeeping, material and label management, and facilities and equipment, accounting for 39.76%, 28.97% and 14.69% respectively. Statistical analysis showed that the significant improvement of laboratory filing requirements (*χ* ^*2*^ = 5.84, *P* = 0.016) was the main reason for the decrease of organization management problems (*χ* ^*2*^ = 5.007, *P* = 0.025). The problems of laboratory housekeeping, experimental material and safety label management had become increasingly prominent (*χ* ^*2*^ = 6.192, *P* = 0.013), especially the nonstandard use of biosafety label (*χ* ^*2*^ = 5.218, *P* = 0.022). Assessment results showed that all found problems as identified risk factors in the past 3 years were determined at the level of medium or low. These suggested that in 2021–2023, the overall laboratory biosafety risk level in Jiaxing was controllable and acceptable, and the organization management had been improved greatly. At the same time, the management of laboratory housekeeping, materials and labels, especially the use of biosafety labels, had become increasingly prominent and need to be standardized and strengthened.

## 1 Introduction

Biosafety (or biosecurity) has become a scientific discipline which has to be implemented to protect public health and safety and to provide a safe environment for scientific growth and protection of individuals. It is extensive and diverse, and related to the fate of a nation, a country, and even the whole mankind (Wang et al., 2022). Laboratory biosafety is a crucial aspect of biosafety management, and it has become an important component of China’s national biosafety concept (Cao, 2021; Xiao and Chen, 2020; Zhao et al., 2020; Wang, 2020). Emergency response for laboratory biosafety accidents and risk assessment and control for laboratory biosafety risks are two important aspects of laboratory biosafety management. Conducting biosafety risk assessment and control by regular internal audit and external quality control inspection is of great significance for a laboratory or institution to operate safely and securely, prevent laboratory biosafety risks and reduce potential accidents (Pavone et al., 2024; Peng et al., 2018; Humblet and Saegerman, 2023).

In China, the SARS epidemic and related biosafety accidents at the end of 2002 made people realize the importance of biosafety management (Inglesby and Cicero, 2017; Lim et al., 2006). With the promulgation of Regulations on Biosafety Management of Pathogenic Microbiology Laboratories in 2004 (State Council Bulletin - China Government Website, 2019), the biosafety management of laboratories in China embarked on a standardized process. By 2018, laboratories in China had generally established laboratory biosafety management systems, and different types and levels of biosafety quality management organizations had been found in various regions, including administrative biosafety management leading groups, advisory expert committees, and technical-guiding laboratory biosafety quality control centers (LBQCCs). With the outbreak of the COVID-19 epidemic in December 2019 and the release of the Biosafety Law in October 2020, the biosecurity work of laboratories across the country had been further strengthened and emphasized (Phyu et al., 2022; Li et al., 2021). Jiaxing, as a coastal city in the north of Zhejiang, adjacent to Shanghai and Hangzhou, less than 100 km away, was greatly affected by the epidemic, and the pressure on laboratory biosafety was highlighted (Zhou and Cen, 2020).

In December 2022, China’s COVID-19 posture was relaxed duly from strict control, and entered a state of normal prevention and control (Diao et al., 2023). Therefore, 2021–2023 became an important period before and after the change of COVID-19 control strategy in China. Additionally, since 2020, with the establishment of the biosafety quality management organization LBQCC in each county, Jiaxing had achieved full coverage of the quality control network, and actively explored and gradually formed a long-term biosafety management model characterized by the collaborative participation from administrative supervision and management, expert technical guidance, and institution quality management. This study was to analyze the problems found in the biosafety quality control inspection of the laboratory in Jiaxing region before and after the strict control of the COVID-19 epidemic in 2021–2023, and to conduct risk assessment of the laboratory biosafety status, so as to propose countermeasures and improvement measures for the biosafety management of Jiaxing in the future.

## 2 Materials and methods

From 2021 to 2023, a total of 40 biosafety quality control inspections of Jiaxing laboratories were carried out, consisting of routine spot inspections by county-level laboratory biosafety quality control centers, and regular spot inspections by Jiaxing Laboratory Biosafety Quality Control Center (Jiaxing LBQCC) and Zhejiang provincial quality control centers (mainly Diagnostic Center and Detection Center), according to the task from the administrative department of Zhejiang Province, more than 40% of the filed/registered laboratories be randomly inspected every year. All inspections were conducted in accordance with the Zhejiang local standard DB33/T 2540 (Zhejiang Provincial Administration for Market Regulation Intellectual Property Office, 2022a; Zhejiang Provincial Administration for Market Regulation Intellectual Property Office, 2022b). It was worth noting that the manual inspection checklists based on the unpublished version of DB33/T 2540 were used before September 2022, and then the Zhejiang Biosafety Quality Control and Evaluation System (referred to as the Evaluation System) based on the published version of the standard was used in the whole province. The system, together with the Zhejiang Pathogenic Microbiology Laboratory Management Information Network (referred to as the Filing System), the Zhejiang Pathogenic Microorganism Transportation Monitoring System, the Zhejiang “Sheng’an Eye” Laboratory Biosafety Monitoring System and the Zhejiang Laboratory Biosafety Visualization Cockpit, had become an integral part of Zhejiang “Biosafety Online” Digital Intelligence Supervision System, and jointly built a “1+4”biosafety supervision matrix in Zhejiang Province (China Reform Newspaper, 2022).

From the inspection data collected from 2021 to 2023, a total of 1,001 problems or risk factors have been identified from 437 filed laboratories in Jiaxing, covering hospitals, disease control centers, blood collection and supply institutions, customs technical service centers, food and drug testing institutes, third-party testing institutions, enterprises and so on. These problems were judged by using 67 third-level indicators, and were sorted into 40 second-level indicators and eight first-level indicators, according to the standard DB33/T 2540. The three-year trends were analyzed by the statistical method Chi-Square Tests (Linear-by-Linear Association) with the software IBM SPSS Statistics version 23 (64- bit edition), and the level of significance was set at 0.05 (*P* = 0.05).

All found problems were regarded as identified risk factors in Jiaxing region, and the risk assessment was carried out by using risk level matrix method, combining the occurrence likelihood of a risk-induced incident with its consequence severity, according to the Chinese industry standard RB/T 040 (CNCA, 2020; National Standards and Technical Evaluation Center of the State Administration for Market Regulation, 2024). This method divides the likelihood of incidents (occurrence frequency) into five levels, 1–5, and divides the severity of the leakage of pathogenic microorganisms and the harm to society (personnel infection, environmental pollution, property damage, and social impact) into five levels, 1–5, and combines the two into the risk matrix table to determine the risk level from four levels: low, medium, high and extremely high.

## 3 Results

### 3.1 Major problems in 2021–2023

**FIGURE 1:**
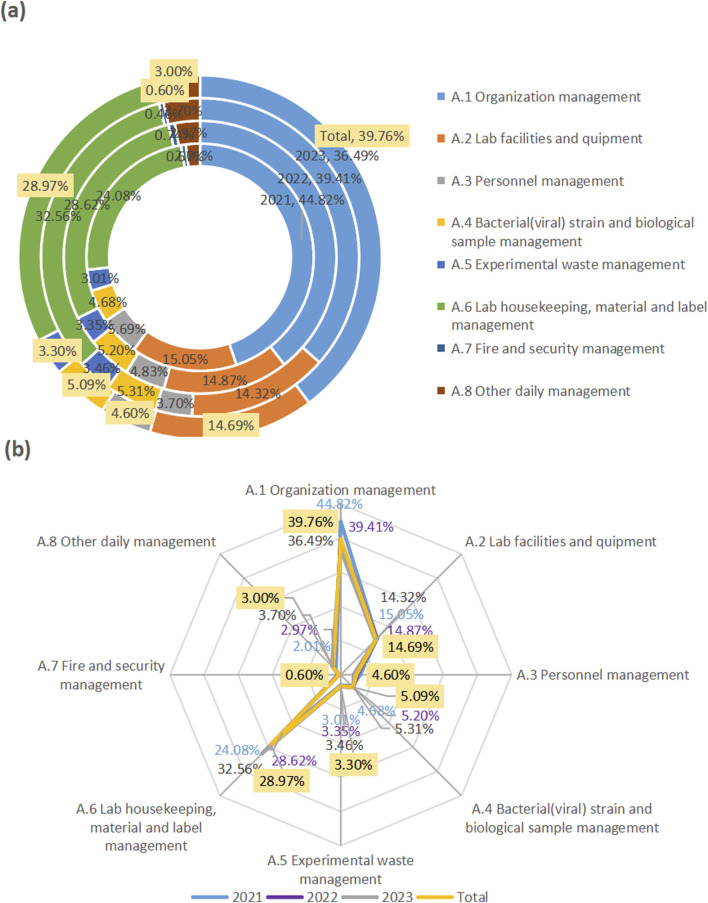
Major problems of biosafety management in Jiaxing in 2021–2023. The rings from inside to outside represent the problems in the years from 2021 to 2023. The “Total”with yellow labels was their respective total over the 3 years. The found problems in the first-level indicators (A1–A8) were displayed in the circular chart showing their proportion **(a)**, and in the radar chart highlighting their contribution degree **(b)**.

In the past 3 years, the major problems of biosafety management found in Jiaxing region came from “organizational management,” “laboratory housekeeping, material and label management,” and “laboratory facility and equipment management,” accounting for 39.76%, 28.97%, and 14.69% respectively (Figure 1).

From data in the three-level indicators, in terms of organizational management, the top three were from biosafety committee (6.49%), laboratory emergency plan (5.19%), risk assessment (5.09%). In terms of “laboratory housekeeping, material and label management,” biosafety label (23.28%), laboratory housekeeping management (3.40%), and disinfection and sterilization management (2.30%) accounted for the top three. In the “laboratory facilities and equipment,” disinfection and sterilization (5.49%), hand-washing, eye-washing and sprinkler devices (4.40%), and biosafety cabinets (1.90%) were among the top three.

### 3.2 Asymptotic analysis at institution level

At the institution level, statistical analysis (Table 1) showed that the problems from organization management had significantly decreased over the past 3 years (*χ* ^*2*^ = 5.007, *P* = 0.025), indicating that organization management had been continuously and significantly improved from 2021 to 2023, and the biosafety management work had been valued and strengthened by lab-setting institutions.

**TABLE 1:** Statistical analysis for found problems in the first-level evaluation indicators.

First-level indicator	2021	2022	2023	χ^2^	*P* ^a^
A.1 Organization management	134	106	158	5.007	0.025
A.2 Lab facilities and equipment	45	40	62	0.080	0.777
A.3 Personnel management	17	13	16	1.635	0.201
A.4 Bacterial (viral) strain and biological sample management	14	14	23	0.137	0.712
A.5 Experimental waste management	9	9	15	0.110	0.740
A.6 Lab housekeeping, material and label management	72	77	141	6.192	0.013
A.7 Fire and security management	2	2	2	0.151	0.697
A.8 Other daily management	6	8	16	1.723	0.189
Total	299	269	433	-	-

^a^ Analysis of Asymptotic Significance (2-sided).

Further analysis (Table 2) showed that data from the second-level evaluation indicator of organizational management, “filing management”, and the third-level indicator “filing requirements,” had significantly decreased, and the statistical values of both were *χ* ^*2*^ = 5.840 (*P* = 0.016). The values were the same because all data of the second-level indicator “filing management” were contributed by those in the third-level indicator “filing requirements,” which were 15, 7, and eight problems found in 2021, 2022, and 2023, respectively. It showed that since 2021, due to the COVID-19, Jiaxing’s administrative departments had significantly strengthened the filing work of biosafety laboratories, and made remarkable achievements in requiring the laboratories to carry out experimental activities in accordance with laws and regulations.

**TABLE 2:** The top 10 of identified risk factors in Jiaxing region.

Top	Risk factor (3rd level indicator)	Classification (1st and 2nd level indicators)	Severity	2021			2022			2023			Total^a^		
				Proportion (%)	Likelihood	Risk level	Proportion (%)	Likelihood	Risk level	Proportion (%)	Likelihood	Risk level	Proportion (%)	Likelihood	Risk level
1	Biosafety label	A.6 Lab housekeeping, material and label management, Lab label management	2	19.06	4	Medium	23.05	4	Medium	26.33	4	Medium	23.28	4	Medium
2	Disinfection and sterilization equipment	A.2 Lab facilities and equipments, Other related equipments	2	6.69	3	Medium	5.58	3	Medium	4.62	2	Low	5.49	3	Medium
3	Lab emergency plan	A.1 Organization management, Emergency treatment	2	5.69	3	Medium	4.09	2	Low	5.54	3	Medium	5.19	3	Medium
4	Risk assessment	A.1 Organization management, Risk assessment and risk control	2	6.02	3	Medium	4.83	2	Low	4.62	2	Low	5.09	3	Medium
5	Lab filing requirements	A.1 Organization management, Lab filing management	2	5.02	3	Medium	2.60	2	Low	1.85	2	Low	3.00	2	Low
6	Biosafety Committee	A.1 Organization management, Organizational structure and responsibilities	1	7.36	3	Low	6.32	3	Low	6.00	3	Low	6.49	3	Low
7	Housekeeping requirements	A.6 Lab housekeeping, material and label management, Lab housekeeping management	2	3.01	2	Low	3.35	2	Low	3.70	2	Low	3.40	2	Low
8	Storing and preservation of bacterial (viral) strains and biological samples	A.4 Bacterial (viral) strain and biological sample management, Use, storage and destruction of bacteria (virus) and biological samples	2	2.34	2	Low	2.60	2	Low	3.00	2	Low	2.70	2	Low
9	Operation of disinfection and sterilization	A.6 Lab housekeeping, material and label management, Disinfection and sterilization management	2	2.01	2	Low	2.23	2	Low	2.54	2	Low	2.30	2	Low
10	Disinfection and sterilization of experimental waste	A.5 Experimental waste management, Experimental waste disposal	2	1.67	2	Low	1.86	2	Low	1.62	2	Low	1.70	2	Low

^a^ Their respective total of identified risk factor in 2021–2023.

In addition, the “other daily management” had no statistically significant difference (*χ* ^*2*^ = 1.723, *P* = 0.189) over the past 3 years, but its third-level evaluation indicators, “internal audit” (*χ* ^*2*^ = 5.164, *P* = 0.023) and “management review” (*χ* ^*2*^ = 3.123, *P* = 0.077), had obvious upward trends in the different significances, which suggested that laboratory-setting institutions should strengthen the internal audit and management review to improve the lab biosafety management work.

### 3.3 Asymptotic analysis at laboratory level

At the laboratory level, it was worth noting that there was a significant increase (Table 1) in the first-level evaluation indicator “laboratory housekeeping, material, and labeling management” in the past 3 years (*χ* ^*2*^ = 6.192, *P* = 0.013). Further analysis showed that its second-level indicator “laboratory label management” (*χ* ^*2*^ = 5.218, *P* = 0.022) and its third-level indicator “biosafety label” (*χ* ^*2*^ = 5.218, *P* = 0.022) had been increasing from 2021 to 2023, with 57, 62, 114 problems found respectively. This indicated that in the past 3 years, the problems in the management of laboratory internal affair, experimental material and safety label, especially the use of biosafety label, had become increasingly prominent and urgently need to be standardized and strengthened.

From the data perspective, there seem to be some increase in the problems related to “bacterial (viral) strain and biological sample management” (*χ* ^*2*^ = 0.137, *P* = 0.712), experimental waste management (*χ* ^*2*^ = 0.110, *P* = 0.740), as well as other daily management (*χ* ^*2*^ = 1.723, *P* = 0.189), though there had been no statistically significant difference over the past 3 years. This suggested that these should be the primary directions for future efforts. Risk identification, correction, prevention, and continuous improvement measures should be strengthened to achieve significant results as soon as possible.

### 3.4 Risk analysis at region level

All risk factors identified or problems discovered in quality control inspections in the past 3 years were determined as “general non-conformities” (according to DB33/T 2540) or level 1 - 2 of consequence severity (according to RB/T 040), due to none of pathogenic microorganism leakage, personnel infection, significant property damage or social impact. The number of medium-level risk factors was 5, 2, 2 from 2021 to 2023 respectively, and the others were at low level (Table 2).

Statistical analysis was conducted on the assessed risk levels of the top 10 identified risk factors (Table 2). The results showed no significant diffrentce in risk levels (*χ* ^*2*^ = 2.071, *P* = 0.150), indicating that the overall biosafety risk levels of laboratories in Jiaxing in the past 3 years, had no significant trend change, and were acceptable and controllable, all being equal to or below the “medium.” So, all laboratory activities were allowed without stopping during inspections or assessments, and the identified risks could be reduced in a timely manner through control measures by laboratories and institutions.

## 4 Discussion

In this study, the local standard DB33/T 2540 of Zhejiang Province was adopted with 67 third-level indicators to identify problems or risks in laboratory biosafety management in Jiaxing, and Chinese industry standard RB/T 040 was adopted for risk assessment. In fact, DB33/T 2540 could also evaluate the found problems from the perspective of compliance with relevant regulations and standards, with a very simple evaluation process, and the possibility and severity of biosafety incidents were comprehensively considered by the evaluation experts. The results were directly provided by the evaluator at the inspection site, including inapplicability, compliance, general non-compliance, and serious non-compliance (Zhejiang Provincial Administration for Market Regulation, 2022b). Generally, a general non-compliance should be rectified (by corrective, preventive, and improvement measures) within a given period of time (usually 1 month). While a serious non-compliance refers to violations of laws, regulations, and technical standards, it means that there is a systematic deficiency in the management system, which can lead to serious biosafety incidents and must be rectified immediately.

It is worth mentioning that the WHO Laboratory Biosafety Manual also uses an assessment method based on a risk matrix (World Health Organization, 2020b; World Health Organization, 2020a), which is similar to China’s RB/T 040 that uses more simplified semi-quantitative results. The probability of an incident and the severity of its consequences are divided into three levels, the former is divided into negligible, moderate and severe, and the latter includes unlikely, possible, and likey, and the final risk level results are very low, low, medium, high and very high. Although the comparisons of methodology and matrix for risk assessment on the three standards mentioned in this study were listed in Table 3, there are no mandatory or rigid requirements for the selection of standards or methods of difficulty and complexity to carry out a biosafety risk assessment.

**TABLE 3:** Comparison of methodology and matrix for risk assessment on 3 standards mentioned in this study.

Standards	DB33/T 2540	WHO laboratory biosafety manual	RB/T 040
Published year	2022	2020 (4th edition)	2020
Purpose and approach	Evaluation of laboratory biosafety management, internal or external	Management of laboratory biosafety, mainly internal	Management of laboratory biosafety risk, internal or external
Assessment methodology (common)	Quality control inspection	Risk matrix	Risk matrix
Quantification of likelihood of risk-induced incident	None	1–3 level: unlikely, possible, likely	1–5 level: unlikely, rare, likely, very likely, and certainly
Quantification of severity of the incident consequence	None	1–3 level: negligible, moderate, severe	1–5 level: no impact, general impact, large impact, significant impact, and particularly significant impact
Determination of risk level	None	1–5 level: very low, low, medium, high, and very high	1–4 level: low, medium, high and extremely high
Result of evaluation item or risk factor	Inapplicability, compliance, general non-compliance and serious non-compliance	Acceptable (controllable) or unacceptable	Acceptable (controllable) or unacceptable
Result application	The “general non-compliance” problems should be rectified within a given period of time (usually 1 month). While a “serious non-compliance” problem can lead to serious biosafety incidents and must be rectified immediately. The results of the evaluation items (as lab biosafety factors) can be indirectly used to control risks	An acceptable risk means that its level is below the allowable level (a predetermined benchmark or criteria), and is considered safe enough to carry out work, otherwise work must be stopped and measures taken to reduce the risk until it falls below that benchmark. Determining the acceptable risk level as a benchmark is essential for risk assessment	An acceptable risk means that its level is below the allowable level (a predetermined benchmark or criteria), and is considered safe enough to carry out work, otherwise work must be stopped and measures taken to reduce the risk until it falls below that benchmark. Determining the acceptable risk level as a benchmark is essential for risk assessment
Benchmark/criteria basis	Compliance (laws, standards and policies)	Compliance (laws, standards and policies), security (fire, theft or loss), socioeconomic impact (environment, agriculture and public health), even the perceptions of relevant stakeholders	Compliance (laws, standards and policies), security (fire, theft or loss), socioeconomic impact (environment, agriculture and public health), even the perceptions of relevant stakeholders
Application effect of risk assessment	Focusing on compliance, it cannot be scientifically and systematically used for biosafety risk assessment, only indirectly for risk control	It can be used scientifically and systematically for risk assessment, and is relatively simple to use compared to RB/T 040	It can be used for risk assessment in a scientific, systematic, and normalized way, but it is a bit cumbersome to use

Usually, risk assessments are carried out for a specific laboratory, and reports for a regional biosafety risk assessment are rarely seen. Moreover, there are no standards and specifications for regional laboratory biosafety risk assessments. In order to systematically understand the overall laboratory biosafety risk status in Jiaxing, this study innovatively attempted to select the relatively complete and complex RB/T 040 to assess the risk status of the whole region, with the whole Jiaxing region regarded as a management body of all laboratories, the problems found in the quality control inspections as the identified risk factors, and the proportion of the problems as the occurrence frequency of risk-related incident in the inspection period. Since the probability of the occurrence of an incident and the severity of its consequences varied depending on the different risk factors, the level results were determined based on the professional experience of the industry reviewers and the actual situation of the laboratory (World Health Organization, 2020b). According to the standard RB/T 040 and from the principle of strict management, this study determined the probability of an incident on a level of 1–5: unlikely, rare, likely, very likely, and certain, with the corresponding proportion of the incident: <1%, 1%–5%, 5%–15%, 15%–30%, and >30% respectively, and determined the severity of incident consequence to 1-5 level: no impact, general impact, large impact, significant impact and particularly significant impact, depending on the degree of the personal infection, property damage and social impact caused by the leakage or release of pathogenic microorganisms. In this study, the severity of the improper use or operational risks easily leading to accidents, such as disinfection and sterilization, medical waste disposal, accident emergency disposal, personal protective equipment, biological safety cabinets and management of biological specimens of bacterial strains, were judged to be level 2, because no reports about pathogen leakage were received in Jiaxing in the past 3 years. Of course, one of the possible situations was that even if a leakage accured, with timely on-site disposal, the scope of the incident was limited into the laboratory and had not caused negative consequences, but such a incident did not need to be reported to superiors, according to Zhejiang Provincial Laboratory Biosafety Emergency Plan (Zhejiang Pathogenic Microbiology Laboratory Biosafety Quality Management Center, 2023).

From the results of this study, the overall risk level of laboratory biosafety in Jiaxing from 2021 to 2023 was at or below the medium level, indicating that the overall situation was controllable. After being standardized and strengthened, the laboratory “filing requirements” during the COVID-2019 epidemic in 2021 (medium level) had ceased to be the most main problem in 2022–2023 (low level), as a major contributor which had led to a significant improvement in organizational management over the past 3 years. While, the highest level (medium) risk factors in 2023 were biosafety label and laboratory emergency plan, and the highest level (medium) risk factors in 2021–2023 were biosafety label, disinfection and sterilization equipment, laboratory emergency plan and risk assessment, suggesting that the biosafety management organizations of Jiaxing region should follow up these risk factors or problems and pay attention to them in the near and long term. In particular, the problem of laboratory “biosafety label” had become the top risk factor, on the one hand, the non-standard use of label was indeed widespread, and on the other hand, it might be related to the subjective focus of inspection experts at different stages, that was, with the obvious improvement of organizational management in the past 3 years, the focus of inspection had shifted from organizational management at the institution level to internal management at the laboratory level.

In fact, whether a laboratory, institution, or regional biosafety management agency (administrative or technical), conducting laboratory biosafety risk assessment and risk control, should choose appropriate standards or approaches according to site-specific situation of the laboratory, the institution or neighborhood. Based on the evaluation, risk control measures, that may include but not limited to corrective, preventive and enhancement, can be implemented in line with the current situation, to minimize risk and maximize sustainability (CNCA, 2020; World Health Organization, 2020b). It is essential for the acceptable risk to determine a benchmark considering actual situations and resources such as the compliance (laws, standards and policies), security (fire, theft or loss), socioeconomic impact (environment, agriculture and public health), even the perceptions of relevant stakeholders (for example, government departments, donors, audit/oversight agencies, general public and local community) (World Health Organization, 2020b; Weng et al., 2024; Bayot and Limaiem, 2023; Bellati et al., 2022). If necessary, appropriate procedures or strategies for risk assessment and control should be developed according to the actual situation and relevant standards or manuals mentioned above, so as to better carry out relevant work (Ruoxi et al., 2018; Haque and Saha, 2020; DiGiandomenico et al., 2020). It is particularly worth mentioning that the importance of personnel training should be emphasized, although its problems did not ranking in the top 10 among all 67 three-level evaluation indicators in Jiaxing, as it plays an critical role in having a comprehensive impact on biosafety management, promoting overall systematic improvement and reducing other risks and hazards (Cornish et al., 2021; Tuncer-Göktuna et al., 2024).

## 5 Conclusion

From 2021 to 2023, just before and after the strict control of the COVID-19 in China, the overall laboratory biosafety level in Jiaxing had been controllable and acceptable, and the organization management had been greatly improved, especially the laboratory filing work. At the same time, new challenges have also emerged. The management of laboratory housekeeping, experimental materials and safety labels, especially the use of biosafety labels, has been becoming increasingly prominent and urgently needs to be standardized and strengthened.

## Data Availability

The original contributions presented in the study are included in the article/Supplementary Material, further inquiries can be directed to the corresponding authors.

## References

[B1] Bayot M. L. Limaiem F. (2023). “Biosafety guidelines,” in StatPearls (Treasure Island, FL: StatPearls Publishing). 30725895

[B2] Bellati M. Russo V. Leone P. A. Zito M. Luperini A. (2022). Biosafety: from a traditional approach to an integrated approach. Front. Public Health 10, 956623. 10.3389/fpubh.2022.956623 35983353 PMC9379254

[B3] Cao C. (2021). China’s evolving biosafety/biosecurity legislations. J. Law Biosci. 8, lsab020. 10.1093/jlb/lsab020 34221436 PMC8245076

[B4] China Reform Newspaper (2022). Building a “1+4” biosafety supervision matrix based on autonomous and controllable IoT key technologies. Available online at: http://www.cfgw.net.cn/epaper/content/202211/04/content_53217.htm (Accessed April 16, 2024).

[B5] CNCA (2020). Guidelines on risk management of biosafety in pathogenic microorganism laboratories. RB/T 040-2020. Beijing, China: Certification and Accreditation Administration of the People’s Republic of China.

[B6] Cornish N. E. Anderson N. L. Arambula D. G. Arduino M. J. Bryan A. Burton N. C. (2021). Clinical laboratory biosafety gaps: lessons learned from past outbreaks reveal a path to a safer future. Clin. Microbiol. Rev. 16 (3), e0012618. 10.1128/CMR.00126-18 PMC8262806 34105993

[B7] Diao C. Tan H. Wen Y. Zhu R. Wu X. Zhang S. (2023). Emotions, COVID-19 related thoughts and satisfaction with life during the critical period from control to relaxation. Front. Psychol. 14, 1211614. 10.3389/fpsyg.2023.1211614 37794904 PMC10546036

[B8] DiGiandomenico K. Dunn E. Sadowski C. Godwin S. Keeler M. Preston F. (2020). Environmental health and biosafety risk assessment guidance for commercial-scale cell and gene therapy manufacturing. Appl. Biosaf. 25, 201–213. 10.1177/1535676020946235 36032393 PMC9134635

[B10] Haque M. S. Saha N. R. (2020). Biosafety measures, socio-economic impacts and challenges of Bt-brinjal cultivation in Bangladesh. Front. Bioeng. Biotechnol. 8, 337. 10.3389/fbioe.2020.00337 32528934 PMC7247817

[B11] Humblet M. F. Saegerman C. (2023). Internal audits as a tool to assess the compliance with biosecurity rules in a veterinary faculty. Front. Vet. Sci. 10, 960051. 10.3389/fvets.2023.960051 36937021 PMC10018162

[B12] Inglesby T. Cicero A. (2017). Protecting the nation from health security threats. Health Secur. 15, 1–5. 10.1089/hs.2016.0122 28092468 PMC5314989

[B13] Li J. Li S. Cao W. Wang Z. Liang Z. Fu W. (2021). Appraisal of China’s response to the outbreak of COVID-19 in comparison with SARS. Front. Public Health 9, 679540. 10.3389/fpubh.2021.679540 34307279 PMC8292770

[B14] Lim W. Ng K. C. Tsang D. N. (2006). Laboratory containment of SARS virus. Ann. Acad. Med. Singap 35, 354–360. 10.47102/annals-acadmedsg.v35n5p354 16830004

[B15] National Standards and Technical Evaluation Center of the State Administration for Market Regulation (2024). Certification and accreditation of standardized information service platform. Available online at: https://hbba.sacinfo.org.cn/stdDetail/0d86937156bafe660c1e1497d4890b8e93e9f9bc6c1657fcefac62436fe91d8c (Accessed April 16, 2024).

[B16] Pavone S. Iscaro C. Giammarioli M. Beato M. S. Righi C. Petrini S. (2024). Biological containment for african swine fever (ASF) laboratories and animal facilities: the Italian challenge in bridging the present regulatory gap and enhancing biosafety and biosecurity measures. Animals 14, 454. 10.3390/ani14030454 38338097 PMC10854939

[B17] Peng H. Bilal M. Iqbal H. M. N. (2018). Improved biosafety and biosecurity measures and/or strategies to tackle laboratory-acquired infections and related risks. Int. J. Environ. Res. Public Health 15 (12), 2697. 10.3390/ijerph15122697 30501091 PMC6313313

[B18] Phyu S. Joseph T. Goulart M. (2022). Strengthening biorisk management in research laboratories with security-sensitive biological agents like SARS-CoV-2. Methods Mol. Biol. 2452, 395–439. 10.1007/978-1-0716-2111-0_23 35554919

[B19] Ruoxi P. Kelsey A. H. Walther M. Pradeep D. U. Benjamin F. (2018). A biocontainment procedure for intravital microscopy of high-risk pathogens. Appl. Biosaf. 23, 211–222. 10.1177/1535676018785177

[B20] State Council Bulletin - China Government Website (2019). Regulations on the management of biological safety in pathogenic microbial laboratories - supplement 1, 2019. Available online at: https://www.gov.cn/gongbao/content/2019/content_5468882.htm (Accessed April 16, 2024).

[B21] Tuncer-Göktuna P. Fontes B. A. Çokçalışkan C. Asar E. Karakaya M. (2024). Implementing an organizational culture of biosafety and biosecurity in the ŞAP Institute. Health Secur. 22 (4), 271–280. 10.1089/hs.2023.0044 38815143

[B22] Wang H. Zhu S. Zhang J. You L. Yin Z. Chu Q. (2022). “Biosafety and the change of world pattern,” in China biosafety: strategies and countermeasures. Editors Wang H. Yan L. Wang H. Lang M. 1st ed. (Beijing, China: Citic Publishing Group), 4–34.

[B23] Wang X. L. (2020). The age of biosecurity:new biotechnology revolution and national biosecurity governance. China Biotechnol. 40 (9), 95–109. 10.14093/j.cnki.cn10-1132/d.2020.04.005 (Chinese).

[B24] Weng S. T. Li Q. W. Gao Y. D. Qiu Y. F. (2024). Biosafety risk control strategies in laboratory animal research. Saf. Health Work 15, 118–122. 10.1016/j.shaw.2023.11.005 38496279 PMC10944157

[B25] World Health Organization (2020a). Laboratory biosafety manual. 4th edition. Available online at: https://www.who.int/publications/i/item/9789240011311 (Accessed April 16, 2024). 24404640

[B26] World Health Organization (2020b). “Risk assessment,” in Laboratory biosafety manual. 3rd edition (Geneva: World Health Organization), 25–45.

[B27] Xiao X. Chen X. (2020). Biosafety Governance under the Overall National Security Concept: Generating Logic, Practical Value, and Path Exploration [J]. Inter. Outlook, 12(5), 21. 10.13851/j.cnki.gjzw.202005007

[B9] Zhao C.H. Su D. D. Li C. Wu Z. Zuo K. Xu Y. (2022). Synthetic biology risks and biosafety strategies in the view of overall national security concept. China Biotech. 42(12), 120–128. 10.13523/j.cb.2206014

[B28] Zhejiang Pathogenic Microbiology Laboratory Biosafety QualityManagement Center (2023). Zhejiang Province pathogenic Microbiology laboratory management health network. Available online at: https://zjsys.wsjkw.zj.gov.cn/xxinfo?uuid=1735497796005023745 (Accessed April 16, 2024).

[B29] Zhejiang Provincial Administration for Market Regulation (Intellectual Property Office) (2022a). Details of local standards_Zhejiang standards online. Available online at: https://zlzx.zjamr.zj.gov.cn/bzzx/public/std/db/view/caa2e31b96e54bafa61b3798de63af8a.html (Accessed April 16, 2024).

[B30] Zhejiang Provincial Administration for Market Regulation (2022b). Specification for the evaluation of biosafety laboratory management. DB33/T 2540-2022. Hangzhou, China: Zhejiang Provincial Administration for Market Regulation.

[B31] Zhou Y. Cen L. S. (2020). Managing acute appendicitis during the COVID-19 pandemic in Jiaxing, China. World J. Clin. Cases 8, 4349–4359. 10.12998/wjcc.v8.i19.4349 33083394 PMC7559659

