# Supplementary material for: Biosafety status analysis and risk assessment of laboratories from 2021 to 2023 in Jiaxing, China
Source: Front Bioeng Biotechnol. 2025 Apr 16;13:1442651. doi: 10.3389/fbioe.2025.1442651 (PMC12040964; doi:10.3389/fbioe.2025.1442651)
Supplement: Supplementary file 1 [file DataSheet1.zip › supplementary/DB33T 2540-2022 《生物安全实验室管理评价规范》 .pdf]

ICS 11.100
CCS C 04

DB33

浙 江 省 地 方 标 准

DB33/T 2540—2022

生物安全实验室管理评价规范

Specification for the evaluation of biosafety laboratory management

2022 - 09 - 30 发布 2022 - 10 - 30 实施

浙江省市场监督管理局 发 布

目　　次

前言 II
1　范围 1
2　规范性引用文件 1
3　术语和定义 1
4　评价方法 2
5　评价内容 2
6　评价程序 2
7　结果判定 2
8　评价结果的应用 3
附录 A（规范性）　生物安全实验室管理评价表 4
附录 B（规范性）　生物安全实验室管理评价结果登记表 46

前 言

本标准按照GB/T 1.1—2020《标准化工作导则　第1部分：标准化文件的结构和起草规则》的规定起草。

请注意本标准的某些内容可能涉及专利。本标准的发布机构不承担识别专利的责任。

本标准由浙江省卫生健康委员会提出、归口并组织实施。

本标准起草单位：浙江省医学科技教育发展中心、中国疾病预防控制中心、浙江省疾病预防控制中心、浙江省产品与工程标准化协会、杭州市疾病预防控制中心、温州市疾病预防控制中心、衢州市疾病预防控制中心、台州市疾病预防控制中心、萧山区第一人民医院、杭州科华实验设备有限公司、浙江省卫康检测科技有限责任公司。

本标准主要起草人：顾华、翁景清、魏强、胡薇薇、李婵、邓铭庭、岑斌、张琪峰、林梅芬、陈卫国、裘丹红、杨振莉、蒋征刚、高颜超、章日春、杨再峰、朱青、包华清、王恩民。

生物安全实验室管理评价规范

1 范围

本标准规定了生物安全实验室管理评价的方法、内容、程序、结果判定和应用。

本标准适用于从事人感染病原微生物研究、教学、检测、诊断实验活动的二级生物安全实验室（bio-safety level 2，以下简称BSL-2）管理的内部或外部评价。一级生物安全实验室（bio-safety level 1，以下简称BSL-1）可参考使用。

2 规范性引用文件

下列文件中的内容通过文中的规范性引用而构成本标准必不可少的条款。其中，注日期的引用文件，仅该日期对应的版本适用于本标准；不注日期的引用文件，其最新版本（包括所有的修改单）适用于本标准文件。

GB 18466 医疗机构水污染物排放标准

GB 19489 实验室 生物安全通用要求

GB 27421 移动式实验室 生物安全要求

GB 50346 生物安全实验室建筑技术规范

WS 233 病原微生物实验室生物安全通用准则

WS 589 病原微生物实验室生物安全标识

国家环境保护总局令第27号 废弃危险化学品污染环境防治办法

中国民用航空局令第216号 危险物品航空安全运输技术细则

卫科教发〔2006〕15号 人间传染的病原微生物名录

3 术语和定义

GB 19489界定的以及下列术语和定义适用于本标准。

3.1

实验室生物安全 laboratory biosafety

实验室的生物安全条件和状态不低于容许水平，可避免实验室人员、来访人员、社区及环境受到不可接受的损害，符合相关法规、标准等对实验室生物安全责任的要求。

[来源：GB 19489—2008，2.13]

3.2

生物安全实验室 biosafety laboratory

通过防护屏障和管理措施，达到生物安全要求的病原微生物实验室。

[来源：WS 233—2017，2.8]

3.3

加强型生物安全二级实验室 enhanced biosafety level 2 laboratory

在普通型生物安全二级实验室的基础上，通过机械通风系统等措施加强实验室生物安全防护要求的实验室，以下简称“加强型BSL-2”。

［来源：WS 233—2017，2.12］

3.4

实验废物　experimental waste

在实验室内产生具有潜在风险的固体、液体和气体类废弃物。

4　评价方法

主要评价方法包括查阅资料、现场观察、询问、操作演示。

5　评价内容

5.1　评价内容应包括下列内容：

a)　组织管理：包括组织架构及职责、管理体系文件、备案管理、风险评估与风险控制、实验活动管理、应急处置、安全检查、个体防护；
b)　实验室设施设备：包括实验室选址和布局、设施设备、防护装备、其他相关设备；
c)　人员管理：包括管理程序、人员准入、培训管理和健康管理；
d)　菌（毒）种和生物样本管理：包括建立相关管理程序、采集及包装、样本运输包装及交接、菌（毒）种和生物样本运输管理、菌（毒）种和生物样本使用、保存、销毁管理；
e)　实验废物管理：包括建立管理程序、人员要求、实验废物管理；
f)　实验室内务及实验材料、标识管理：包括内务管理程序、管理要求、消毒和灭菌管理、实验材料管理、标识管理；
g)　消防安保管理：包括建立管理程序、消防管理、安全保卫、保密管理；
h)　其他日常管理：包括安全计划、内部审核、管理评审、不符合项的识别和控制、纠正措施、预防措施、持续改进。

5.2　具体评价内容应符合附录A的规定。

6　评价程序

评价应按照下列程序进行：

a)　组建评价小组，至少由3名实验室生物安全管理和技术专家组成，设组长1名，并确定评价计划；
b)　召开首次会议；
c)　按照本标准要求开展现场评价；
d)　确定不符合项并提出改进建议；
e)　召开末次会议，反馈评价结果。

7　结果判定

评价结果分为符合、一般不符合、严重不符合及不适用：

a)　符合是指与相关法律、法规、技术标准和管理体系的规定相一致；

b) 一般不符合是指属于个别的、偶然的、孤立的失效现象，一般不会引发严重生物安全事件；

c) 严重不符合是指违反法律、法规、技术标准规定，管理体系存在系统缺陷，会引发严重生物安全事件。

8 评价结果的应用

8.1 评价小组应将评价结果和改进建议反馈给被评价机构，并经被评价机构的法定代表人或经授权的生物安全负责人签字确认。评价结果表应符合附录B的规定。

8.2 评价结果用于被评价机构生物安全管理的改进和风险控制。

8.3 被评价机构应组织做好不符合项整改工作，针对评价结果，采取纠正措施，消除安全隐患。

附 录 A
（规范性）
生物安全实验室管理评价表

A.1 生物安全实验室管理评价表（组织管理）应符合表A.1的要求。

表A.1 生物安全实验室管理评价表（组织管理）

评价指标	评价内容	评价方法	评价结果			
一、组织架构及职责	**（一）法人资格：**<br>实验室设立单位或实验室母体组织应具有明确的法律地位和从事相关实验活动的资格。	**查阅资料**<br>1.查看法人证书。<br>2.查看非独立法人机构是否有母体组织授权的证明材料。	符合	一般不符合	严重不符合	不适用
	**（二）法定代表人职责：**<br>1.对单位实验室生物安全负总责。<br>2.批准成立生物安全委员会。<br>3.负责指定单位层面的生物安全负责人及其他关键职位代理人。<br>4.负责组织建立单位层面的生物安全管理体系。<br>5.落实生物安全管理责任部门和责任人。<br>6.每年至少召开1次生物安全管理工作会议，对实验室生物安全相关的重大事项做出决策。<br>7.批准发布实验室生物安全管理手册和程序文件体系文件。	**查阅资料**<br>1.查看生物安全委员会文件是否由法人签发。<br>2.查看单位层面实验室生物安全管理体系文件是否由法人签发。<br>3.查看体系文件是否包含单位层面的实验室生物安全负责人、实验室负责人、安全监督员等关键岗位代理人。<br>4.查看生物安全管理责任部门和责任人文件。<br>5.查看生物安全管理工作会议和活动记录。<br>**询问**<br>1.询问法定代表人是否熟悉生物安全相关法律、法规要求。<br>2.询问法定代表人是否熟悉单位实验室生物安全管理的总体要求。<br>3.询问法定代表人是否了解自身应承担的管理职责。	符合	一般不符合	严重不符合	不适用

表A.1　生物安全实验室管理评价表（组织管理）（续）

评价指标	评价内容	评价方法	评价结果			
一、组织架构及职责	**（三）生物安全委员会：**<br>1.实验室设立单位应成立生物安全委员会，组成人员应包含单位法定代表人、生物安全负责人、实验室负责人、生物安全管理部门负责人、相关部门负责人及相关领域技术专家。<br>2.生物安全委员会工作职责：<br>（1）负责组织管理和技术专家对实验室设立和运行进行咨询、指导、监督、评估（如风险评估和应急处置）；<br>（2）对单位生物安全管理体系文件进行审核，并督促落实；<br>（3）对新开展的实验活动项目、技术、方法安全性进行评估；<br>（4）对实验室生物安全建设设计、设施设备、个体防护、消毒灭菌进行评估与指导；<br>（5）对生物安全事件的调查、评估、认定、处置进行指导；<br>（6）每年至少开展 1 次实验室生物安全管理体系运行的评价，并提出改进意见。<br>3.应记录相关活动内容并保存归档。	**查阅资料**<br>1.查看实验室设立单位成立单位生物安全委员会的有效文件，人员是否调整更新，职责是否明确。<br>2.查看生物安全委员会是否按照职责要求履行相关责任。<br>3.查看相关活动记录是否完整、规范，信息是否齐全、真实。	符合	一般不符合	严重不符合	不适用
	**（四）生物安全管理责任部门职责：**<br>1.负责生物安全管理体系的设计、实施、维持和改进，并对其运行情况进行监督。<br>2.制定或修订生物安全管理体系文件，每年至少组织 1 次体系文件的评审。<br>3.对实验项目进行审查或风险评估。<br>4.对实验人员的健康监测和管理。<br>5.组织生物安全培训与考核。<br>6.负责单位实验室备案。	**查阅资料**<br>1.查看是否每年至少组织 1 次对生物安全管理体系的评审，是否有开展体系运行监督检查。<br>2.查看是否开展对高致病性病原微生物实验项目的安全性审查。<br>3.查看开展实验活动、设施设备、实验材料、个体防护、菌（毒）种和生物样本、实验废物、信息安全及消防安全监管记录。<br>4.查看相关管理活动的过程性资料和记录是否齐全完整。<br>5.查看体系文件宣贯活动的通知、签到表、课件、记录。	符合	一般不符合	严重不符合	不适用

表A.1 生物安全实验室管理评价表（组织管理）（续）

评价指标	评价内容	评价方法	评价结果			
一、组织架构及职责	7.每年至少组织开展1次内部审核、管理评审和系统性监督检查。<br>8.开展体系文件宣贯。	**询问**<br>1.询问相关部门负责人和管理人员对法律、法规、标准以及单位管理体系文件是否熟悉。<br>2.询问是否明确自己的岗位职责并按照要求开展相关管理活动。	符合	一般不符合	严重不符合	不适用
	**（五）实验室负责人职责：**<br>1.实验室负责人为实验室生物安全的第一责任人，全面负责实验室生物安全工作，做好日常管理。<br>2.负责实验项目计划、方案和操作规程的审查并监督落实。<br>3.决定并授权人员进入实验室，并做好相关人员培训。<br>4.落实实验室备案，负责实验室活动的管理、风险评估及意外事件的处置。<br>5.指定实验室生物安全负责人、项目负责人、内务管理监督员，赋予其监督相关活动的职责和权力。<br>6.负责实验室相关的菌（毒）种和生物样本全过程安全监管。<br>7.督促落实建立实验活动记录档案，按规定保存。<br>8.负责组织从事高致病性病原微生物实验活动人员健康监测和免疫接种。<br>9.组织开展实验室内部安全检查，纠正违规行为并有权做出停止实验的决定。	**查阅资料**<br>1.查看实验室负责人的任命文件。<br>2.查看实验项目计划、方案、操作规程、人员登记有无实验室负责人签字。<br>3.查看实验室培训记录是否齐全。<br>4.查看实验室备案信息是否与管理信息网信息一致。<br>5.查看负责人是否按照要求组织开展对实验人员和辅助人员进行能力评估及培训、实验活动风险评估、感染控制、个体防护及监督管理活动的情况。<br>6.查看菌（毒）种和生物样本出库审批是否由实验室负责人签字。<br>7.查看实验室人员免疫接种计划、健康监护和实施情况是否由实验室负责人签字及记录。<br>8.查看实验室对外合作项目审查，外来人员（包括横向合作、参观、实习进修人员）、辅助人员准入审查记录。<br>9.查看实验室内部安全检查记录。<br>**询问**<br>1.询问实验室负责人对法律、法规、标准及单位管理体系文件是否熟悉，是否了解自身职责。	符合	一般不符合	严重不符合	不适用

表A.1　生物安全实验室管理评价表（组织管理）（续）

评价指标	评价内容	评价方法	评价结果			
一、组织架构及职责		2.询问实验室生物安全监督员、内务管理员、项目负责人是否由实验室负责人指定并按照要求开展工作。	符合	一般不符合	严重不符合	不适用
	**（六）实验人员职责：**<br>1.应充分认识和理解所从事工作的风险。<br>2.应自觉遵守实验室的管理规定和要求。<br>3.在身体状态许可的情况下，应接受实验室的免疫计划和其他的健康管理规定。<br>4.应按规定正确使用实验设施、设备和个体防护装备。<br>5.应主动报告可能不适于从事特定任务的个人状态。<br>6.不应因人事、经济任何压力而违反管理规定。<br>7.有责任和义务避免因个人原因造成生物安全事件或事故。<br>8.应主动识别任何危险和不符合工作，若怀疑个人受到感染，立即报告。	**操作演示**<br>抽查部分实验人员现场操作生物安全柜、个体防护装备、消毒设备。<br>**现场观察**<br>查看实验人员是否遵守相关规定，如个体防护、感染控制。<br>**询问**<br>1.询问实验人员是否清楚个人责任和岗位职责。<br>2.询问实验人员是否清楚和了解自己从事实验活动的风险。	符合	一般不符合	严重不符合	不适用
二、管理体系文件	**（七）体系文件编制：**<br>1.实验室设立单位编制生物安全管理体系文件，包括生物安全管理手册、程序文件、标准操作规程、安全手册、记录表格。<br>2.明确生物安全管理方针和目标。<br>3.明确各部门的职责，包括生物安全委员会、各相关职能部门和实验室的职责。<br>4.明确关键岗位人员的职责，生物安全管理手册中应包括生物安全负责人、生物安全技术负责人、实验室负责人、项目负责人及关键职位的代理人。	**查阅资料**<br>1.查看生物安全管理体系文件是否完整齐全，是否现行有效。<br>2.查看生物安全管理方针和目标是否包括实验室的工作范围，对管理活动和技术活动制定的安全指标是否明确、可考核。<br>3.查看体系文件中各部门职责是否明确。<br>4.查看关键岗位人员职责是否明确。<br>5.查看体系文件是否按规定批准、发布。<br>6.管理体系日常运行是否和体系文件规定相一致。<br>**询问**<br>询问相关人员是否熟悉体系文件具体内容和规定。	符合	一般不符合	严重不符合	不适用

表A.1　生物安全实验室管理评价表（组织管理）（续）

评价指标	评价内容	评价方法	评价结果			
二、管理体系文件	5.体系文件应经批准后发布实施，其中管理手册、程序文件应经法定代表人批准，其它技术文件应由指定人员批准。		符合	一般不符合	严重不符合	不适用
	**（八）体系文件控制：**<br>1.实验室设立单位应建立文件控制程序，对文件的编制、审核、批准、标识、发放、保管、修订、废止、回收、销毁及存档环节进行有效控制。<br>2.相关场所应能够随时获得现行有效的体系文件。	**查阅资料**<br>1.查看体系文件格式是否完整，包括（标题、文件编号、版本号、修订号、页数、生效日期、编制人、审核人、批准人、参考文献、编制依据）。<br>2.查看是否建立文件控制程序，并按照程序要求运行和记录。<br>3.查看相关现场能否随时获得体系文件。	符合	一般不符合	严重不符合	不适用
三、备案管理	**（九）备案要求：**<br>1.新建、改建、扩建的BSL-1、BSL-2（包括固定，以及车载、方舱、气膜移动实验室）应向设区的市级卫生行政主管部门备案。<br>2.实验室应以独立的物理区域单元进行备案。<br>3.应保证备案信息完整、真实并与实际相符，实验室场所、关键设施设备、主要实验人员和实验活动项目发生变更时，应在变更始 1个月内向原备案机构提交实验室备案变更报告，重新进行备案登记。	**查阅资料**<br>查验实验室备案信息与管理信息网是否一致，是否在规定时间内更新。<br>**现场观察**<br>1.观察是否将不同楼层或物理分隔相对独立的实验室备案成同一个实验室。<br>2.观察是否存在应备未备的实验室。	符合	一般不符合	严重不符合	不适用
四、风险评估和风险控制	**（十）风险评估：**<br>1.实验室设立单位应建立风险评估和风险控制程序。<br>2.由单位管理部门组织开展风险评估活动，风险识别的范围应包括：致病性生物因子的已知或未知特性、涉及致病性生物因子的实验活动、涉及遗传修饰生物体时应考虑重组体引起的危害、感染性废物处置过程中的风险、涉及致病性生物因子实验活动的相关人员、实验室设施设备（包括：电气、门禁、水、监控报警等）、已	**查阅资料**<br>1.查看是否制定了风险评估和风险控制管理程序。<br>2.查看风险识别的范围是否覆盖齐全。<br>3.查看风险评估报告内容是否规范完整。<br>4.查看是否每年至少进行 1 次风险评估报告评审。	符合	一般不符合	严重不符合	不适用

表A.1　生物安全实验室管理评价表（组织管理）（续）

评价指标	评价内容	评价方法	评价结果			
四、风险评估和风险控制	发生的实验室感染事件的原因分析，重点识别所保藏的或使用的致病性生物因子被盗、滥用和恶意释放的风险。<br>3.应由具有经验的不同领域的专业人员根据国家法律、法规、标准、规范开展评估。<br>4.风险评估应形成书面报告，至少包括：实验活动（项目计划）简介、评估目的、评估依据、评估方法/程序、评估内容、评估结论，从事高致病性病原微生物实验活动的每种病原应形成单独风险评估报告。<br>5.风险评估报告应经生物安全委员会审核，并经法定代表人批准后发布。<br>6.单位管理部门应每年组织1次风险评估报告的评审；若法律、法规、标准、政策、关键设施设备、人员等发生较大变化，或开展新的实验活动、发生意外事件情况时，应进行风险再评估。<br>7.应依据风险评估结论采取相应的风险控制措施，采取风险控制措施时宜优先考虑控制风险源，再考虑采取其他措施降低风险。	5.查看风险评估报告是否经单位生物安全委员会审核和法定代表人批准。<br>6.查看风险评估报告是否进行及时更新。<br>7.查看风险评估报告和风险控制措施是否结合单位实际情况。<br>8.查看风险控制是否按照风险评估有关要求开展实施。	符合	一般不符合	严重不符合	不适用
五、实验活动管理	**（十一）实验活动：**<br>1.实验室应用计划、申请、批准、实施、监督和评估实验活动，以及针对未知风险材料操作的政策和程序。<br>2.对我国尚未发现或者已经宣布消灭的病原微生物，未经批准不应从事相关实验活动。<br>3.实验室使用新技术、新方法从事高致病性病原微生物相关实验活动的，应符合防止高致病性病原微生物扩散、保证生物安全和操作者人身安全的要求。<br>4.在同一个实验室的同一个独立安全区域内，只能同时从事一种高	**查阅资料**<br>1.查看是否建立实验活动的相关程序。<br>2.核查实验室是否违规开展实验活动（重点检查科学研究项目），查看是否存在超范围开展实验活动，或超出备案实验室防护等级实验活动。可通过抽查实验原始记录，进行反向追溯验证。<br>3.高致病性病原微生物实验活动新技术、新方法是否经过安全性认证。<br>4.查看生物安全信息管理系统中是否有实验活动填报的证明	符合	一般不符合	严重不符合	不适用

表A.1　生物安全实验室管理评价表（组织管理）（续）

评价指标	评价内容	评价方法	评价结果			
五、实验活动管理	致病性病原微生物的相关实验活动。<br>5.从事高致病性病原微生物相关实验活动应有2名以上人员共同进行。<br>6.从事高致病性病原微生物实验活动每年应向省级卫生行政主管部门报告实验活动结果和实验数据情况。<br>7.BSL-1、BSL-2不应从事应在BSL-3及以上生物安全实验室开展的高致病性病原微生物实验活动。<br>8.实验室应建立实验档案，真实完整记录实验室开展实验活动情况，每年归档。高致病性病原微生物相关实验活动档案保存期限不应少于20年。	性材料。<br>5.查看开展的实验活动是否在相应防护级别的生物安全实验室中进行。<br>6.查看是否建立实验档案，体系运行记录和高致病性病原微生物相关活动的实验记录，是否完整并归档保存。<br>**现场观察**<br>1.查看是否在独立区域同时开展2种及以上未经灭活的高致病性病原微生物实验材料操作。<br>2.通过视频采集及现场观察确认是否存在独自1人开展高致病性病原微生物实验活动。	符合	一般不符合	严重不符合	不适用
六、应急处置	**（十二）实验室应急预案：**<br>1.实验室设立单位应制定应急处置管理程序。<br>2.应制定单位层面应急预案，内容应至少包括：生物性、化学性、物理性、放射性意外事故，以及火灾、水灾、冰冻、地震或破坏突发紧急情况。<br>3.应急预案应至少包括：组织机构、应急原则、人员职责、应急通讯、个体防护、应对程序、应急设备、撤离计划和路线、污染源隔离和消毒灭菌、人员隔离和救治、现场隔离和控制、风险沟通。<br>4.应急预案应经生物安全委员会审核后由法定代表人批准，应急预案应向省级卫生行政主管部门备案。<br>5.应急预案应每年进行审查、更新。<br>6.实验室设立单位相关人员应熟悉应急预案，每年至少组织1次培训和应急处置演练。	**查阅资料**<br>1.查看是否制定了单位层面的应急预案、应急处置及报告程序。<br>2.查看应急处置管理程序内容是否完整、流程是否清晰合理和具有可操作性。查看应急预案内容是否完整，是否包括总则、组织体系与职责、实验室感染事故的分类分级、监测与预警、报告、应急响应、现场流行病学调查与处理、后期处理、实验室感染的预防内容，流程是否清晰合理和具有可操作性。<br>3.查看实验室设立单位应急预案是否向省级卫生行政主管部门进行备案。<br>4.查看是否每年审核、更新应急预案。<br>5.查看实验室演练记录并查看是否覆盖全部相关人员。<br>**询问**<br>现场询问相关人员是否了解应急预案主要内容。	符合	一般不符合	严重不符合	不适用

表A.1 生物安全实验室管理评价表（组织管理）（续）

评价指标	评价内容	评价方法	评价结果			
六、应急处置	**（十三）应急物资储备：**<br>1.应急物资的配备应与实验活动相适应，种类齐全（包括：个人防护装备、消杀用品、急救箱、应急照明灯、洗眼器、洒溢和锐器处理器材等），并按使用说明书进行维护。<br>2.实验室应配备消防器材、急救箱及应急处置器材常用物资。	**现场观察**<br>1.查看应急物资储备种类是否齐全有效。。<br>2.查看应急物资的储备情况及清单。	符合	一般不符合	严重不符合	不适用
	**（十四）事件报告及处置：**<br>1.实验室发生意外事件，工作人员应按照应急预案迅速采取控制措施，同时应按制度报告，任何人员不应瞒报、谎报、迟报、漏报。<br>2.事故现场紧急处理后，应记录事件发生过程和现场处置情况。<br>3.实验室负责人应及时对事件作出危害评估并提出下一步对策。对事件经过和事件原因、责任进行调查分析，形成书面报告。报告应包括事件的详细描述、原因分析、影响范围、预防类似事件发生的建议及改进措施。所有事件报告应形成档案文件并存档。<br>4.事件报告应经所在机构管理层、生物安全委员会评估。<br>5.若发生高致病性病原微生物菌（毒）种和样本在运输、储存、使用过程被盗、被抢、丢失及实验人员发生疑似感染情况的，责任单位应在2小时内向主管部门报告，同时通报当地公安机关。	**查阅资料**<br>查看是否按照规定报告和处置，资料是否完整。<br>**询问**<br>询问实验室相关人员是否发生过意外事件或事故。	符合	一般不符合	严重不符合	不适用
七、安全检查	**（十五）安全检查要求：**<br>1.实验室设立单位应组织开展监督检查，每年应至少系统性地检查1次，对关键控制点可根据风险评估报告适当增加检查频率。	**查阅资料**<br>1.查看实验室设立单位有无监督检查工作计划，检查内容是否覆盖全部要素。<br>2.查看是否按计划组织开展监督检查，核查检查记录。<br>3.查看对发现的问题是否组织整改及跟踪验证，并形成记录。	符合	一般不符合	严重不符合	不适用

表A.1　生物安全实验室管理评价表（组织管理）（续）

评价指标	评价内容	评价方法	评价结果			
七、安全检查	2.实验室设立单位应制定监督检查计划，检查风险控制措施的有效性，包括对实验人员的操作、设备的使用、新方法的引入以及大量样本检测等内容，应将高致病性病原微生物菌（毒）种和样本的操作、菌（毒）种及样本保管、实验室操作规范、实验室行为规范、个人防护及感染控制、废物处理等作为监督的重点。<br>3.实验室应每季度组织 1 次开展内部自查。<br>4.监督检查内容包括但不限于本标准规定内容.。<br>5.当发现不符合规定的工作、发生事件或事故时，应立即查找原因并评估后果；必要时，停止工作。在监督检查过程中发现的问题要立即采取纠正措施，并监控所取得的效果，以确保所发现的问题得以有效解决。		符合	一般不符合	严重不符合	不适用
八、个体防护	**（十六）个体防护要求：**<br>1.生物安全防护等级与其拟从事的实验活动相适应。<br>2.个人防护措施应满足开展的实验活动风险控制要求。<br>3.应为实验人员提供如何在风险最小情况下进行工作的详细指导，包括正确选择和使用个体防护装备。<br>4.应配备足够数量的防护装备，如生物安全柜。	**查阅资料**<br>查看相关标准操作规程，是否存在安全风险。<br>**现场观察**<br>1.查看实验防护等级是否能满足该实验活动的风险控制要求。<br>2.查看个体防护是否符合实验活动风险评估要求，如有防护口罩、防护面罩，是否进行过适配性测试。<br>3.查看关键防护装备种类和数量是否足够。	符合	一般不符合	严重不符合	不适用

A.2　生物安全实验室管理评价表（实验室设施设备）应符合表A.2的要求。

表A.2　生物安全实验室管理评价表（实验室设施设备）

评价指标	评价内容	评价方法	评价结果			
			符合	一般不符合	严重不符合	不适用
一、实验室选址和布局	**（十七）实验室设计及基本要求：**<br>1.实验室设计和建造应符合国家和地方环境保护和建设等主管部门的规定和要求。<br>2.如适用，建筑要求应满足GB 50346有关要求。<br>3.实验室的防火和安全通道设置应符合国家的消防规定和要求，防火等级不宜低于2级。<br>4.实验室的建筑材料和设备等应符合国家相关部门对该类产品生产销售和使用的规定和要求。<br>5.实验室的设计保证对生物、化学、辐射和物理等危险源的防护水平控制在经过评估的可接受程度，为关联的办公区和邻近的公共空间提供安全的工作环境，及防止危害环境。<br>6.实验室的走廊和通道应不妨碍人员和物品通过。<br>7.应设计紧急撤离路线，紧急出口应有明显的标识。<br>8.需要时（如正当操作危险材料时），房间的入口处应有警示和进入限制。<br>9.应评估生物材料、样本、药品、化学品和机密资料等被误用、被偷盗和被不正当使用的风险，并采取相应的物理防范措施，高致病性病原微生物菌（毒）种应有独立的存放场所，并落实双人双锁、防盗、视频监控等措施。<br>10.应有专门设计确保接收、存储、转运、收集、处理和处置危险物料的安全。	**查阅资料**<br>1.查看消防设计图看实验室防火等级是否符合要求。<br>2.查看移动式生物安全实验室的说明书、出厂合格证明、使用说明书、性能检测报告相关材料。<br>3.查看实验室环境记录，必要时检测温湿度和检测噪音。<br>**现场观察**<br>1.查看实验室建筑的设计、工作流程是否符合要求。必要时，可查阅实验室设计图纸资料。<br>2.查看实验室防火和安全通道设置是否符合要求。<br>3.查看地面、墙面、台柜是否符合安全设计要求。<br>4.查看实验室走廊是否通畅，是否存在堆放杂物妨碍人员通行或撤离。<br>5.查看实验室门锁安装是否符合要求，是否影响逃生。<br>6.查看是否设置防节肢动物和啮齿动物进入和外逃的物理措施。				

表A.2 生物安全实验室管理评价表（实验室设施设备）（续）

评价指标	评价内容	评价方法	评价结果			
			符合	一般不符合	严重不符合	不适用
一、实验室选址和布局	11.实验室内温度、湿度、照度、噪声和洁净度等室内环境参数应符合工作和卫生等相关要求。<br>12.实验设计应考虑节能环保及舒适性要求，应符合职业卫生和人机工效学要求。<br>13.实验室应有防止节肢动物和啮齿动物进入的措施。<br>14.实验室台（桌）柜和座椅应稳固和坚固，边角应圆滑。实验台面应防水，能耐受中等程度的热、有机溶剂、酸碱、消毒剂及其他化学剂。<br>15.实验室台柜等和其摆放应便于清洁，实验台面应防水、耐腐蚀、耐热和坚固。<br>16.BSL-1 温度宜为 18℃～28℃，BSL-2 核心工作间温度宜为 18 ℃～26 ℃，相对湿度宜为 30%～70%，噪音应低于 68 dB。<br>17.加强型 BSL-2 实验室设计还应符合 WS 233 的要求。<br>18.移动式生物安全实验室应按照 GB 27421 执行。	7.观察实验台柜是否牢固，边角是否圆滑。<br>8.观察实验室地面台面是否耐腐蚀。<br>9.查看移动式生物安全实验室的内部布局和工作流程是否符合，设备设施是否满足工作需求，固定装置是否可靠，实验室是否与工作地区的道路和自然条件相适应。				
	**（十八）平面布局：**<br>1.实验室宜相对独立，BSL-2 应与办公及其它公共区域有物理隔离。<br>2.BSL-2 实验室平面布局一般采用辅助区（准备间）和核心工作间布局方式。<br>3.加强型 BSL-2 实验室一般应采用辅助区（准备间）、缓冲区和核心工作间布局方式。<br>4.实验室应有足够的空间摆放实验室设备和物品。	**现场观察**<br>1.查看实验区域是否相对独立，分区是否合理。<br>2.查看实验室区域是否有足够的空间开展实验活动。<br>3.查看实验室内是否有足够的物品存放区域。<br>4.查看人员和货物进出通道有效分流。<br>5.查看层高是否满足要求。				

表A.2 生物安全实验室管理评价表（实验室设施设备）（续）

评价指标	评价内容	评价方法	评价结果			
一、实验室选址和布局	5.在实验室门口处应设存衣或挂衣装置，可将个人服装与实验室工作服分开放置，BSL-2 实验室工作区域外应有存放备用物品的条件。<br>6.实验室应将人流和货物通道分流，应设计紧急逃生路线。<br>7.应根据工作性质和流程合理摆放实验室设备、台柜、物品等，避免相互干扰、交叉污染，并应不妨碍逃生和急救。<br>8.实验室内净高不宜低于 2.6 米。<br>9.移动式生物安全实验室的高度应满足设备安装要求，应有摆放、操作、维护和清洁空间。		符合	一般不符合	严重不符合	不适用
	**（十九）围护结构：**<br>1.实验室的门应有可视窗并可锁闭，BSL-2 实验室主入口及核心操作间门应能自动关闭，主入口门应有出入控制措施，门锁及门的开启方向应不妨碍室内人员逃生，负压实验室门宜向外开。<br>2.实验室墙壁、顶板和地板应光滑、易清洁、不渗水、防渗漏并耐化学品和消毒剂腐蚀。地面应平整、防滑，不应在实验室内铺设地毯。<br>3.负压及加强型 BSL-2 实验室及移动式实验室的围护结构应密闭。应有措施防止产生对人员健康产生的异常压力，围护结构应能承受送风机或排风机异常时导致的空气压力载荷。	**现场观察**<br>1.观察实验室门是否有可视窗能否观察到实验操作区域及可锁闭。2.查看 BSL-2 实验室主入口及核心操作间门能否自动关闭，查看负压实验室门是否向外开。<br>3.观察天花板是否光滑，不积尘，观察地面是否防滑、平整、易清洁，有无铺设地毯问题。<br>4.查看实验室围护结构是否足够牢固，能承受异常压力的载荷。<br>观察负压或加强型 BSL-2 实验室围护结构是否密闭，是否存在气体泄漏风险。	符合	一般不符合	严重不符合	不适用

表A.2 生物安全实验室管理评价表（实验室设施设备）（续）

评价指标	评价内容	评价方法	评价结果			
			符合	一般不符合	严重不符合	不适用
二、实验室设施设备	**（二十）通风空调系统：**<br>1.自然通风：实验室可以利用自然通风。如果有可开启的窗户，应安装可防蚊虫的纱窗。<br>2.机械通风：<br>（1）如果采用机械通风，应避免交叉污染。机械通风空调净化系统应有利于实验室消毒灭菌、自动控制系统的设置和节能运行。<br>（2）实验室可采用有循环风的空调系统；风机应选用风压变化较大时风量变化较小的类型。<br>（3)BSL-2 中操作非经空气传播生物因子的实验室和可有效利用安全隔离装置进行操作的实验室可采用带循环风的空调系统。不能有效利用安全隔离装置进行操作的实验室宜采用全排风系统，开展高致病性病原微生物实验活动应加装高效过滤器装置。<br>（4）生物安全实验室的室内排风口处应设高效过滤器。送排风系统中高效过滤器不应重复使用，送排风管道应隐蔽安装，其咬口缝均应用胶密封。<br>（5）送风口和排风口应采取防雨、防风、防杂物、防昆虫及其他动物的措施，送风口应远离污染源和排风口。<br>（6）新风口采取有效的防雨措施，应有防鼠、防昆虫、阻挡绒毛的防护网，便于拆卸，高度应高于室外地面 2.5 米以上，同时远离污染源或实验室的排风口。<br>（7）排风气密阀应设在排风高效过滤器和排风机之间，排风机外侧的排风管上室外排风口处应安装保护网和防雨罩。<br>（8）实验室的排风应与送风连锁，排风先于送风开启，后于送风关闭。	**现场观察**<br>1.自然通风：<br>（1）查看自然通风是否安装防蚊虫纱窗。<br>（2）查看实验过程中门窗是否处于关闭状态。<br>2.机械通风：<br>（1）查看空调通风系统是否存在交叉污染或人员感染风险和隐患。<br>（2）查看有循环风的空调系统排风气密阀的设置位置，排风口末端处是否有保护网和防雨罩。<br>（3）查看送、排风管道及咬口缝情况看是否严密。<br>（4）查看是否存在循环使用防护区的空气现象。<br>（5）查看高效过滤器的材质是否为一次性使用。<br>（6）查看新风口是否远离排风口，避免交叉污染或引起人员感染。<br>（7）查看排风口是否安装了高效过滤器。<br>（8）查看送、排风、新风口是否都有防雨、防虫措施。<br>（9）查看核心工作间内送、排风口的布置位置是否合理，排风口应远离新风口并处在新风口的下风向。				

表A.2　生物安全实验室管理评价表（实验室设施设备）（续）

评价指标	评价内容	评价方法	评价结果			
二、实验室设施设备	3.气流组织：<br>（1）核心工作间内送风口和排风口的布置应符合定向气流的原则，利于减少房间内的涡流和气流死角，利于可能被污染空气的排出。<br>（2）生物安全实验室宜采用上送下排方式。<br>（3）在生物安全柜操作面或其他气溶胶产生地点的上方附近不应设送风口。<br>（4）高效过滤器排风口应设在室内被污染风险最高区域，不应有障碍。<br>（5）气流组织上送下排时，高效过滤器排风口下边沿离地面低于0.1米或高于0.15米，或上边沿超过地面0.6米;排风口风速大于1米/秒。<br>4.其他：<br>（1）操作有毒、有害、溶媒、强刺激性、强致敏性材料的BSL-2实验室，应配备通风橱或二级B型生物安全柜。<br>（2）加强型BSL-2通风空调系统核心工作间气压相对于相邻区域应为负压，压差宜不低于10Pa。在核心操作区入口的显著位置，应安装显示房间负压状况的压力显示装置。<br>（3）加强型BSL-2实验室防护区换气次数不少于12次/小时。洁净度应达到七到八级，空气过滤系统应设置三级过滤，末端设高效过滤器。<br>（4）移动式生物安全实验室通风空调系统应按照GB 27421执行。	3.气流组织：<br>（1）机械通风需查看送风口和排风口设置位置是否符合气流组织要求（从洁净区域向防护区域单向流动），是否上送下排。<br>（2）查看实验室空调或送风口气流是否干扰生物安全柜前窗的气幕屏障。<br>（3）查看相邻区域压差是否能保持在10Pa及以上。<br>（4）查看负压实验室是否会形成乱流或涡流及气流死角。<br>4.其他：<br>（1）查看操作现场是否涉及有毒、有害、溶媒材料操作，是否按照要求配备生物安全柜或通风橱或采用全新风系统。<br>（2）查看加强型BSL-2是否有操作间的压差显示，并符合要求。<br>（3）加强型BSL-2，查看送、排风机是否联锁，实现排风先起，后于送风关闭。<br>（4）查看加强型BSL-2实验室、移动式BSL-2实验室的核心实验间入口处是否有压力显示装置，压力值是否与设定的范围一致，是否有压力过载报警功能。<br>（5）加强型BSL-2换气次数及洁净度是否符合要求。	符合	一般不符合	严重不符合	不适用

表A.2　生物安全实验室管理评价表（实验室设施设备）（续）

评价指标	评价内容	评价方法	评价结果			
二、实验室设施设备	**（二十一）供水与供气系统：**<br>1.供水：<br>（1）应满足实验室所需用水。<br>（2）给（排）水管道应设置防倒流装置或其他有效的防止回流污染的装置，并且这些装置应设置在辅助工作区；给排水系统下水应有防回流设计。<br>（3）给水排水管道系统宜采用不锈钢管、铜管或无毒塑料管材料，应不渗漏、耐压、耐温、耐腐蚀，管道应可靠连接。<br>（4）实验室排水系统上的通气管口应单独设置，不应接入空调通风系统的排风管道。<br>（5）给水排水管道穿越生物安全实验室防护区围护结构处应设可靠的密封装置，密封装置的严密性应能满足所在区域的严密性要求。<br>（6）移动式生物安全实验室宜预留市政供水接口，可设置下水收集装置。如下水外排，应以风险评估为依据。<br>2.供气：<br>（1）供气（液）罐，应放在实验室防护区外易更换和维护的位置，安装牢固，不应将不相容的气体或液体放在一起。<br>（2）若使用高压气体和可燃气体，应有安全措施，应符合国家、地方的相关规定和要求。（3）气体管道系统应不渗漏、耐压、耐温、耐腐蚀。	**现场观察**<br>1.供水：<br>（1）查看实验室是否设有给、排水。<br>（2）查看给、排水管道是否设防回流装置，排水装置是否有存水弯。<br>（3）查看水管的材质是否符合要求，连接是否牢固，有无渗漏现象。<br>（4）查看是否将排水系统上的通气关口接入空调通风管道。<br>（5）查看穿过防护区的水管或气管及线管是否可靠密封。<br>（6）查看移动式生物安全实验室如有用水直接下排的，排水口是否设置下水收集装置。<br>2.供气：<br>（1）查看供气（液）罐是否有固定装置，安装是否牢固，是否存在不相容气体混放现象。<br>（2）查看高压气体和可燃气体使用是否符合相关规定。<br>（3）查看气体管道系统是否不渗漏、耐压、耐温、耐腐蚀。	符合	一般不符合	严重不符合	不适用

表A.2　生物安全实验室管理评价表（实验室设施设备）（续）

评价指标	评价内容	评价方法	评价结果			
二、实验室设施设备	**（二十二）门禁管理系统：**<br>1.实验室主入口的门应有门禁系统。<br>2.BSL-2出入口的门、放置生物安全柜实验间的门应可自动关闭。<br>3.加强型BSL-2应在实验操作区和辅助区之间设置缓冲间，缓冲间的门宜能互锁。<br>4.移动式BSL-2核心实验间入口宜设置缓冲间，缓冲间可兼作防护服更换间，门宜能互锁。如果使用互锁门，应在互锁门的附近设置紧急手动解除互锁开关。	**现场观察**<br>1.查看实验室主出、入口有无门禁设施，门禁是否处于正常状态。<br>2.查看是否设有有效的人员出入控制措施。<br>3.查看BSL-2出入口的门、放置生物安全柜实验间的门是否可自动关闭。<br>4.查看缓冲间的两道门是否有互锁和紧急解锁装置。	符合	一般不符合	严重不符合	不适用
	**（二十三）电力供应与照明：**<br>1.应有可靠和足够的电力供应，必要时重要设备（如培养箱、生物安全柜、冰箱）应配置备用电源或不间断电源（UPS）。<br>2.应设专用配电箱，用电负荷不宜低于2级。<br>3.应配备足够的固定电源插座，避免多台设备使用共同的电源插座。应有可靠的接地系统，应在关键节点安装漏电保护装置或监测报警装置。<br>4.应保证实验室内有足够的照明，照度不小于300Lx，避免不必要的强反光和闪光。<br>5.应设应急照明装置，实验室所有疏散出口都应有消防应急照明，同时考虑合适的安装位置，以保证人员安全离开实验室。	**现场观察**<br>1.现场查看电力供应情况，询问有无UPS或备用电源或双路供电。<br>2.查看是否设有专用配电箱。<br>3.查看是否存在多台设备共用插线板或多个接线板串联供电和私拉电线问题。<br>4.查看照明灯的设置位置和光照度是否符合要求。必要时可现场检测光照度。<br>5.查看有无应急照明装置，是否可正常使用，查看应急照明维持时间能否达到30分钟。	符合	一般不符合	严重不符合	不适用

表A.2　生物安全实验室管理评价表（实验室设施设备）（续）

评价指标	评价内容	评价方法	评价结果			
二、实验室设施设备	**（二十四）洗手、洗眼及喷淋装置：**<br>1.实验室应设洗手池，宜设置在靠近出口处，水龙头开关宜为非手动式。若实验室不具备供水条件，应设非手动手消毒装置。<br>2.实验室涉及刺激性或腐蚀性物质的操作，BSL-1 应在 30 米内设洗眼装置；BSL-2（含移动式生物安全实验室）应在实验室操作区配备洗眼装置。<br>3.实验室（含移动式生物安全实验室）若大量使用刺激或腐蚀性物质，必要时应在 30 米内设紧急喷淋装置。	**现场观察**<br>1.查看有无洗手装置，是否为非手动式，位置是否合理。<br>2.查看是否配备洗眼装置，是否每周维护 1 次，是否洁净、不受污染，是否可正常使用。<br>3.查看洗眼装置是否安装在规定范围内。<br>4.查看实验室是否需要配备喷淋装置。如安装了喷淋装置，是否维护，并能正常使用。喷淋装置的设置位置是否符合要求。	符合	一般不符合	严重不符合	不适用
二、实验室设施设备	**（二十五）自控及通讯系统：**<br>1.自控系统：<br>（1）实验室如有自控系统应具备自动控制和手动控制的功能。<br>（2）空调净化自控系统应保证各房间之间定向流方向的正确及压差的稳定。<br>（3）加强型 BSL-2 应通过自动控制措施保证实验室压力及压力梯度的稳定性，并可对异常情况报警。<br>2.通讯系统：<br>（1）实验室核心工作间应配备适用的通讯设备。<br>（2）实验室内宜设数据传输网络端口，用于实验数据的传输。<br>（3）移动式生物安全实验室应安装行车定位系统和行车记录系统，应保持通讯联络畅通。	**现场观察**<br>1.自控系统：<br>（1）查看自控系统是否具备自动和手动控制功能。<br>（2）查看自控系统是否能保证定向气流和压差。<br>（3）查看自控系统是否稳定可靠运行，各参数显示是否灵敏、稳定。如气压、压差、送排风机切换，异常报警。<br>2.通讯系统：<br>（1）查看实验室核心工作间是否配备通讯设备和设施。<br>（2）查看是否在设有数据传输设备。如内部网络端口。<br>（3）移动式生物安全实验室查看车载定位系统是否正常运行并有行车记录。	符合	一般不符合	严重不符合	不适用

表A.2 生物安全实验室管理评价表（实验室设施设备）（续）

评价指标	评价内容	评价方法	评价结果			
三、防护装备	**（二十六）生物安全柜：**<br>1.BSL-2应在操作病原微生物及样本的实验区内（移动式BSL-2为核心实验间内）配备二级生物安全柜。<br>2.应按产品的设计、使用说明书的要求安装和使用生物安全柜。<br>3.生物安全柜放置的位置应合理。在安全柜的背后和周边宜留有30厘米的空间，背面最少不少于3.8厘米，两侧不少于8厘米，用于清洁维护。<br>4. A1和A2型可直接排在房间或通过套管连接排到室外。顶端排气口和天花板之间的间距最少应有8厘米。A2型用于进行以微量挥发性有毒化学品和痕量放射性核素为辅助剂的微生物实验室时，应连接功能合适的排气罩。<br>5.涉及挥发性有毒化学品和放射性核素为辅助剂的实验材料操作时，宜选用B1和B2型生物安全柜。B1和B2型生物安全柜应有专用的排气系统，排气系统应包括防漏管道、管道内靠近安全柜的调节气阀、外排气风扇。安全柜与管道密闭连接，气体排入专用管道，管道内风机或建筑物系统联锁，应有报警系统提示安全柜排气流量的损失。<br>6.安全柜用的屋顶排气系统应有竖直向上的排气管道直接延伸到屋顶面3米以上。<br>7.生物安全柜应由具备相应资质的机构按照相应的检测规程进行检测，每年至少检测1次。<br>8.如果生物安全柜的排风在室内循环，室内应具备通风换气的条件；如果使用需要管道排风的生物安全柜，应通过独立于建筑物其他公共通风系统的管道排出。	**查阅资料**<br>查看年度检测报告和标识是否处于有效期内。<br>**现场观察**<br>1.查看操作区是否配备合适的生物安全柜，查看型号是否与实验活动相匹配。<br>2.查看是否有操作规程并按规程操作，必要时现场演示，有使用及维护记录。<br>3.查看摆放位置是否合理，空调等气流是否会对生物安全柜前窗气流屏障产生干扰。<br>4.查看后侧和顶部是否留有检测、维护通道。<br>5.查看外部外排风管的排风口位置和高度是否符合要求，是否存在对周围环境影响风险。<br>6.查看生物安全柜和实验室排风管道的连接方式是否正确，材料是否符合要求。<br>7.查看外排风管是否向上，管道材料是否符合要求，是否存在泄露、脱落、破裂、老化风险。<br>8.如果使用管道排风的生物安全柜，查看是否通过独立于建筑物其他公共通风系统的管道排出，排风量是否与实验室设计匹配。	符合	一般不符合	严重不符合	不适用

表A.2 生物安全实验室管理评价表（实验室设施设备）（续）

评价指标	评价内容	评价方法	评价结果			
四、其他相关设备	**（二十七）消毒和灭菌设备：**<br>1.应在实验室或其所在的建筑内配备压力蒸汽灭菌器或其他适当的消毒、灭菌设备，所配备和存放的消毒、灭菌设备应以风险评估为依据。<br>2.压力灭菌器应按照说明书进行检定，压力表每半年检定 1 次，安全阀每年检定 1 次。<br>3.开展压力蒸汽灭菌的效果监测与验证，包括生物监测、化学监测、物理监测，每一次灭菌周期均应进行化学监测，医疗机构至少每月开展 1 次生物学监测。<br>4.应根据实验室使用情况对防护区进行消毒。<br>5.如安装紫外灯，应监测紫外灯的辐射强度（用辐照计或强度照度指示卡进行监测），至少每季度开展 1 次，如用以使用寿命时长指示照度的紫外灯应对使用时长进行记录。<br>6.移动式 BSL-2 应配备适宜的消毒灭菌装置，需要时，应配备高压蒸汽灭菌器。	**查阅资料**<br>1.查看实验室设立单位是否有实验室消毒管理程序。<br>2.查看压力灭菌器消毒设备检定报告（包括压力表、安全阀的检定）。<br>3.查看是否按照要求开展实验室消毒。<br>4.查看实验室消毒灭菌、效果监测、评价和维护记录，消毒灭菌效果监测评价频次是否满足要求。<br>**现场观察**<br>1.查看是否按照规定要求配备消毒灭菌设备。<br>2.查看灭菌器类型、数量是否符合要求。	符合	一般不符合	严重不符合	不适用
	**（二十八）设施设备档案:**<br>1.应按照 GB 19489 的要求建立设施设备档案。<br>2.实验室应有对设施设备（包括个体防护装备）管理的政策和运行维护保养程序。<br>3.设施设备维护、修理、报废需移出实验室时，移出前应先进行消毒去污染。<br>4.应依据制造商的建议和使用说明书进行使用、维护实验室设施设备，说明书应便于有关人员查阅。	**查阅资料**<br>1.查看是否建立设备档案，档案资料是否完整、规范<br>2.查看是否有设备管理程序，包括设施设备性能指标的监控、日常巡检、安全检查、校准和检定、维护保养，编写设备操作规程。<br>3.查看设备是否有维护记录。<br>4.查看是否有设备使用说明或操作规程。<br>5.查看是否有设备状态标签。	符合	一般不符合	严重不符合	不适用

表A.2 生物安全实验室管理评价表（实验室设施设备）（续）

评价指标	评价内容	评价方法	评价结果			
四、其他相关设备	5.实验室大型精密设备、仪器，未经实验室负责人许可不应擅自移动。<br>6.实验室设备应按照要求粘贴运行状态标识。	**现场观察**<br>1.查看存在被污染的设备是否进行去污染处理。<br>2.查看设备是否正常运行，必要时可要求操作演示。<br>3.查看关键设备是否被随意移动。	符合	一般不符合	严重不符合	不适用

A.3 生物安全实验室管理评价表（人员管理）应符合表A.3的要求。

表A.3 生物安全实验室管理评价表（人员管理）

评价指标	评价内容	评价方法	评价结果			
一、管理程序	**（二十九）实验室人员管理程序：**<br>1.实验室设立单位应建立人员管理程序。<br>2.配备的人力资源应满足实验活动和生物安全管理体系有效运行的要求。	**查阅资料**<br>查看是否制定人员管理程序。<br>**询问**<br>询问管理人员或其他相关人员，单位人力资源是否能满足实验活动和生物安全管理体系运行的基本要求。	符合	一般不符合	严重不符合	不适用
二、人员准入	**（三十）实验室人员准入管理：**<br>1.实验人员和管理人员应有相关的教育背景或工作经历，具有开展相关岗位工作的能力。<br>2.实验室应规定人员岗位准入资格。<br>3.实验室管理部门和实验室负责人应对进入实验区域的相关人员（包括参观访问、进修、实习、设备维修、外单位合作、后勤保障外来人员）进行准入审核和批准。	**查阅资料**<br>1.查看实验室人员的上岗准入资格要求，并抽查检验人员或管理人员是否满足规定要求。<br>2.查看实验室准入审核、批准登记记录。<br>**询问**<br>询问实验人员专业知识和生物安全知识，需要时现场观察其操作能力。	符合	一般不符合	严重不符合	不适用
三、人员培训管理	**（三十一）实验室人员培训：**	**查阅资料**	符合	一般不符合	严重不符合	不适用

表A.3 生物安全实验室管理评价表（人员管理）（续）

评价指标	评价内容	评价方法	评价结果			
三、人员培训管理	1.实验室设立单位应对实验人员（包括实验辅助人员、外来人员）进行设施设备、个体防护、消毒技术、专业技术基础知识和规范操作等内容的培训，每年至少组织1次。<br>2.实验室设立单位应对新进人员进行岗前专业技术和生物安全培训，经考核合格后才允许上岗。<br>3.对从事高致病性病原微生物实验活动的人员应每半年进行1次培训，并记录培训及考核情况，每年进行1次能力评价。<br>4.从事病原微生物实验活动的相关人员应取得岗位培训证书，经考核合格后持颁发的“浙江省病原微生物实验室生物安全培训合格证书”取得上岗资格。生物安全管理及实验人员每3年至少参加1次省级及以上继续教育培训。	1.查看实验室设立单位的培训记录，接受培训的人员是否全覆盖（包括相关职能部门责任人、实验人员、管理人员、辅助人员）。培训的资料是否齐全，是否包括培训通知、培训课件/资料、签到表、考核表和培训效果评价相关材料。<br>2.查看新进人员培训和考核记录，到岗1年内是否参加了生物安全培训，并取得培训合格证。<br>3.查看从事高致病性病原微生物实验活动人员是否每半年至少参加1次的培训记录。<br>4.查看实验及相关人员是否持有培训合格证及继续教育培训证。	符合	一般不符合	严重不符合	不适用
四、健康管理	**（三十二）实验室人员健康管理：**<br>1.实验室设立单位应组织实验人员及相关人员至少每年参加1次健康体检，并进行健康评估，必要时应进行预防接种。<br>2.在开展高致病性病原微生物实验活动时，应对实验人员进行健康监护。<br>3.实验室设立单位应建立实验室人员健康档案。	**查阅资料**<br>1.查看相关人员是否有健康体检相关记录，查看是否有相关免疫接种计划及记录，查看从事高致病性病原微生物实验活动人员是否有健康监护记录。<br>2.查看高致病性病原微生物实验活动相关岗位人员是否采集、留取了本底血清。<br>3.查看实验室人员是否有健康档案，是否包括：岗位风险说明及知情同意书（必要时）、本底血清样本或特定病原的免疫功能相关记录、预防免疫记录、健康体检报告、职业感染和职业禁忌症资料、与实验室安全相关的意外事件、事故报告。<br>**询问**<br>询问是否存在实验室工作人员在身体状况不适时进入实验室的现象。	符合	一般不符合	严重不符合	不适用

A.4　生物安全实验室管理评价表（菌(毒)种和生物样本管理）应符合表A.4的要求。

表A.4　生物安全实验室管理评价表（菌(毒)种和生物样本管理）

评价指标	评价内容	评价方法	评价结果			
一、管理程序	**（三十三）菌(毒)种和生物样本管理程序：**<br>实验室菌(毒)种或生物样本保存、使用管理，制定选择、购买、采集、包装、转运、接收、查验、使用、销毁、处置和保藏的政策和程序。	**查阅资料**<br>查看实验室设立单位是否建立了菌(毒)种和生物样本管理程序，内容是否齐全。	符合	一般不符合	严重不符合	不适用
二、采集及包装	**（三十四）菌(毒)种和生物样本采集及包装：**<br>1.对各类病原微生物生物样本的采样制定标准操作规程，并包含基于生物安全考虑的因素。<br>2.具有与采集病原微生物生物样本所需要的生物安全防护水平相适应的设备和装备。<br>3.工作人员具有掌握相关专业知识和操作技能。<br>4.采集病原微生物生物样本的工作人员在采集过程中应防止病原微生物扩散和感染。<br>5.详细记录样本的来源、采集过程和方法。<br>6.具有能有效防止病原微生物扩散和感染的措施，能保证病原微生物生物样本质量的技术方法和手段。<br>7.包装材料应符合国家有关要求。	**查阅资料**<br>1.查看单位是否制定各类病原采样的标准操作规程。<br>2.查看采样人员采样培训和能力考核记录。<br>3.查看采样记录，是否符合要求。<br>4.查看各类生物样本是否选择合适的包装容器，是否符合防渗漏、防破碎要求。<br>5.查看是否贴有风险识别的标签，附有相关样本基本信息，包括样品名称、来源、采样时间。<br>**现场观察**<br>观察采样人员个人防护（工作服、隔离衣、戴工作帽、医用防护口罩和手消）和采集设施设备是否与所采集生物样本的风险防护等级相适应。<br>**询问**<br>现场询问采样人员是否熟知采样要求和风险，了解如何防止病原微生物扩散和感染的措施。	符合	一般不符合	严重不符合	不适用

表A.4 生物安全实验室管理评价表（菌(毒)种和生物样本管理）（续）

评价指标	评价内容	评价方法	评价结果			
三、样本运输包装及交接	**（三十五）菌(毒)种和生物样本运输包装及交接：**<br>1.外部运输应按照《人间传染的病原微生物名录》选用合适的运输箱，使用三层包装系统，并应有规范的生物危险标签、标识、警告用语和提示用语。<br>2.运输高致病性病原微生物菌（毒）种或生物样本的容器或包装材料应达到国际民航组织《危险物品航空安全运输技术细则》（Doc9284 包装说明 PI602）规定的 A 类包装标准，符合防水、防破损、防外泄、耐高温、耐高压的要求，宜安装远程追踪定位系统，并应印有国家卫生行政主管部门规定的生物危险标签、标识、运输登记表、警告用语和提示用语。<br>3.高致病性病原微生物菌（毒）种或生物样本在运输之前的包装以及送达后包装的开启，应在符合生物安全规定的场所和设施设备条件下进行。运输前后均应检查包装的完整性，并核对菌(毒)种或生物样本的数量。<br>4.菌(毒)种或生物样本运输交接文件至少包括病原微生物名称、性质、数量、交接时包装的状态、交接人、收发交接时间和地点。<br>5.单位内部转运感染性及潜在感染性物质应置于被证实和批准的具有防渗漏、防溢洒的容器中运输。	**现场观察**<br>1.查看机构外部转运容器是否采用符合生物安全要求的专用转运工具。<br>2.查看高致病性病原微生物菌（毒）或生物样本的包装容器是否符合要求。<br>3.必要时查看是否具备高致病性病原微生物菌(毒)种或生物样本接收的场所和设施设备条件。<br>**询问**<br>1.询问管理人员和操作人员是否清楚外部转运的包装和标识要求。<br>2.询问工作人员应在哪里开启包装，并查交接记录或接收证明（接收时包装情况，时间，送达人员、接收样本数量信息）。	符合	一般不符合	严重不符合	不适用
四、菌(毒)种和生物样本运输管理	**（三十六）菌(毒)种和生物样本运输申请：**<br>1.高致病性病原微生物菌（毒）种或生物样本（包括非高致病性病原微生物运输包装分类为 A 类的病原微生物菌（毒）种或样本，以及疑似高致病性病原微生物菌（毒）种或样本）的运输应进行审批。	**查阅资料**<br>1.查看是否持有《可感染人类的高致病性病原微生物菌（毒）种或样本准运证书》（以下简称《准运证》），运输项目和区域是否相符合。<br>2.查看跨市转运是否有运输申请、审批手续。	符合	一般不符合	严重不符合	不适用

表A.4 生物安全实验室管理评价表（菌(毒)种和生物样本管理）（续）

评价指标	评价内容	评价方法	评价结果			
四、菌(毒)种和生物样本运输管理	2.在省内运输的，应向所在地市级卫生行政主管部门申请；需要跨省、自治区、直辖市运输或者运往国外的，由出发地的省级卫生行政主管部门初审后，报国家卫生行政主管部门批准。<br>3.对于为控制传染病暴发、流行或者突发公共卫生事件应急处理的高致病性病原微生物菌（毒）种或生物样本的运输申请，可以通过传真的方式进行上报和审批；需要提交有关材料原件的，应于事后尽快补齐；需要向中国疾病预防控制中心运送高致病性病原微生物菌（毒）种或生物样本的，向中国疾病预防控制中心直接提出申请，由中国疾病预防控制中心审批。<br>4.在固定的申请单位和接收单位之间多次运输相同品种高致病性病原微生物菌（毒）种或样本的，可以申请多次运输。多次运输的有效期为6个月；期满后需要继续运输的，应重新提出申请。<br>5.在运输结束后，申请单位应将运输情况向原批准部门书面报告。	3.查看运输申请表的出发地、目的地是否和《准运证》一致。<br>4.属于突发事件的应急样本，查看是否按要求补办了相关审批。<br>5.查看是否有多次运输的审批件，《准运证》是否在有效期内。<br>6.查看运输高致病性病原微生物菌（毒）种和生物样本是否有运输后的报告资料。	符合	一般不符合	严重不符合	不适用
四、菌(毒)种和生物样本运输管理	**（三十七）菌(毒)种和生物样本承运单位要求：**<br>1.承运单位（包括实验室设立单位、第三方承运机构）应具有相应的承运资质、安全保障能力和资源保障。严禁委托给不具备感染性生物材料运输资质的部门进行运输。<br>2.承运单位应严格执行分类包装、专用交通工具、冷链专车运输安全保障措施。<br>3.高致病性病原微生物样本运输应落实全程实时监控和可追溯的风险管控措施。<br>4.承运单位应开展运输相关的风险评估，落实风险控制措施，并制定样本转运规范和应急预案。	**查阅资料**<br>1.查看和询问承运单位（包括第三方承运机构）的资质（准运证）和安全保障能力证明材料。<br>2.查看承运单位的运输工具和硬件保障措施。<br>3.查看高致病性病原微生物运输是否能够全程可追溯。<br>4.查看承运单位的应急预案。<br>5.查看是否按照合法路径运输。<br>6.查看委托单位和承运单位是否有委托合同并明确安全责任。<br>7.查看承运单位落实有关安全管控及交接记录资料。<br>8.查看承运人员的培训记录和准入资质，有航空运输的，查看运输人员资质《中国民用航空危险品运输训练合格证》。	符合	一般不符合	严重不符合	不适用

表A.4 生物安全实验室管理评价表（菌(毒)种和生物样本管理）（续）

评价指标	评价内容	评价方法	评价结果			
四、菌(毒)种和生物样本运输管理	5.承运单位应严格按照国家的要求通过合法的路径进行运输。运输高致病性病原微生物菌（毒）种或者样本，应通过陆路运输；没有陆路通道，必须经水路运输的，可通过水路运输；紧急情况下或者需要将高致病性病原微生物菌（毒）种或者样本运往国外的，可以通过民用航空运输。<br>6.承运单位不应把承运业务分包给不具备安全保障能力的机构或个人进行运输。<br>7.运输过程中一旦发生病原微生物菌（毒）种和生物样本丢失、泄露、溢洒或被抢、被盗，应按照规定程序向有关部门报告，并采取有效措施，防止事故影响范围的扩大，降低危害程度和后果。<br>8.不应通过公共交通工具、城市铁路进行运输，不应通过邮寄方式进行运输。<br>9.承运单位应建立运输的相关记录档案，高致病性应至少保存 20年。<br>10.承运单位应对参与运输的人员组织开展生物安全相关的专项培训与能力考核，持证上岗，通过航空运输的人员应有《中国民用航空危险品运输训练合格证》。<br>11.高致病性病原微生物菌（毒）种或生物样本运输应有专人护送，护送人员不应少于 2 人。<br>12.菌(毒)种或生物样本运输应防止人员感染及环境污染，必要时，在运输过程中有个体防护设备及有效消毒剂。	**询问**<br>1.询问是否存在通过公共交通工具、城市铁路、邮寄进行运输的现象。<br>2.查看运输是否有专人护送，高致病性病原微生物菌（毒）种或生物样本护送人员是否至少 2 人。<br>3.询问运送人员在运输时是否配备个体防护设施和消毒剂。	符合	一般不符合	严重不符合	不适用
五、菌(毒)种和生物样本使用保存、销毁管理	**（三十八）菌（毒）种和生物样本使用：**<br>1.菌(毒)种或生物样本在使用过程中应有专人负责，高致病性病原微生物双人双锁管理，入库、出库及销毁应记录并存档。	**查阅资料**<br>1.查看菌（毒）种或生物样本的出、入库记录和销毁记录是否符合要求，是否可追溯。	符合	一般不符合	严重不符合	不适用

表A.4 生物安全实验室管理评价表（菌(毒)种和生物样本管理）（续）

评价指标	评价内容	评价方法	评价结果			
五、菌(毒)种和生物样本使用保存、销毁管理	2.高致病性菌（毒）种和生物样本的使用应该得到实验室设立单位管理层批准。<br>3.保存的菌（毒）种应进行检查，符合要求后投入使用，并记录。<br>4.对领用的高致病性菌（毒）种和生物样本使用进行全程管控。<br>5.未经批准任何单位和实验室不应接收高致病性病原微生物菌（毒）种或生物样本。<br>6.未经批准不应进行菌（毒）种和生物样本的交流、交换或赠予。	2.查看是否有菌（毒）种和生物样本的使用审批记录。<br>3.查看是否有菌（毒）种检查的符合性记录。<br>4.查看是否有菌（毒）种的领用、使用记录。<br>5.查看高致病性病原微生物菌（毒）种或生物样本是否在种类和数量上和接收记录一致。<br>**询问**<br>询问是否存在未经批准进行菌（毒）种和生物样本的接收、交流、交换或赠予情况。	符合	一般不符合	严重不符合	不适用
	**（三十九）菌（毒）种和生物样本保存及保藏：**<br>1.实验室应至少有 2 名工作人员负责菌（毒）种及感染性样本的管理。<br>2.应对所有危险材料建立清单，包括来源、接收、使用、处置、存放、使用权限、事件和数量内容。<br>3.保存菌（毒）种及感染性样本容器的材质、质量应符合安全要求，不易破碎、爆裂、泄露。<br>4.保存容器上应有牢固的标签或标识，标明菌（毒）种及感染性样本的编号、日期信息。<br>5.实验室应将在研究、教学、检测、诊断、生产实验活动中获得的有保存价值的各类菌（毒）种或感染性样本送交保藏机构进行鉴定和保藏。<br>6.高致病性病原微生物相关实验活动结束后，应在 6 个月内将菌（毒）种或感染性样本就地销毁或者送交保藏机构保藏。	**查阅资料**<br>1.查看和询问是否建立菌（毒）种目录，是否落实 2 名工作人员管理。<br>2.查看菌（毒）种目录、传代记录、出入库记录、使用记录、销毁记录是否符合要求。<br>3.查看菌（毒）种保存容器是否符合要求。<br>4.查看各类菌（毒）种或感染性样本是否有交接记录。<br>5.查看保藏清单台账，是否和实物相符。<br>6.查看是否在 6 个月内将高致病性病原微生物菌（毒）种和感染性样本就地销毁或送交保藏机构，是否有销毁、交接记录。<br>**现场观察**<br>随机抽查数个菌（毒）种，查看相关信息。	符合	一般不符合	严重不符合	不适用

表A.4　生物安全实验室管理评价表（菌(毒)种和生物样本管理）（续）

<table>
<tr><th>评价指标</th><th>评价内容</th><th>评价方法</th><th colspan="4">评价结果</th></tr>
<tr><td rowspan="3">五、菌(毒)种和生物样本使用保存、销毁管理</td><td>（四十）保存的设施设备：
1.实验室应具备菌（毒）种及感染性样本适宜的保存区域，区域内配置相应的保存设备。高致病性病原微生物菌（毒）种和感染性样本设立专库专柜，单独储存。
2.保存区域应有菌（毒）种及感染性样本检查、交接、包装的场所，具有生物安全柜设备。
3.应有可靠的物理措施和管理程序。
4.菌（毒）种库应配备稳定的电力供应，并有断电报警。</td><td>现场观察
1.查看是否具有保存菌（毒）种或生物样本的专用场所、空间，高致病性病原微生物菌（毒）种和感染性样本是否设立专库专柜，单独储存。
2.查看是否具有超低温冰箱、离心机基本保存设备。
3.查看是否具有检查、交接、包装的专用场所（接收区或发放区），并具有生物安全柜样本接收和发放的基本设备。
4.查看通风散热、平面布局、电力供应异常报警是否符合条件。</td><td>符合</td><td>一般不符合</td><td>严重不符合</td><td>不适用</td></tr>
<tr><td>（四十一）菌（毒）种和生物样本销毁：
1.销毁病原微生物菌（毒）种或感染性样本时应采用国家标准、技术规范、指南规定的方法。
2.高致病性病原微生物菌（毒）种或感染性样本的销毁应办理销毁审批手续。
3.销毁高致病性病原微生物菌（毒）种工作应在与拟销毁菌（毒）种相适应的生物安全实验室内进行，由2人共同操作，并对销毁过程进行监督和记录。</td><td>查阅资料
查看销毁记录，是否选用合适的销毁方式。
询问
1.询问高致病性病原微生物菌（毒）种或感染性样本的销毁是否办理销毁审批手续。
2.询问高致病性病原微生物菌（毒）种的销毁是否有相关部门的监督人员在场，是否有监督销毁的证明记录。</td><td>符合</td><td>一般不符合</td><td>严重不符合</td><td>不适用</td></tr>
<tr><td>（四十二）菌（毒）种和生物样本资料归档：
1.高致病性病原微生物菌(毒)种和生物样本采样、转运、保存（保藏）、使用、销毁相关记录，应建立档案。
2.高致病性菌（毒）种和生物样本相关记录安全保存，保存期限不少于20年。</td><td>查阅资料
1.查看是否在程序文件中规定菌（毒）种相关记录保存期限。
2.查看是否建立菌（毒）种或生物样本明细台账。</td><td>符合</td><td>一般不符合</td><td>严重不符合</td><td>不适用</td></tr>
</table>

A.5 生物安全实验室管理评价表（实验废物管理）应符合表A.5的要求。

表A.5 生物安全实验室管理评价表（实验废物管理）

评价指标	评价内容	评价方法	评价结果			
一、管理程序	**（四十三）实验废物管理程序：**<br>实验室设立单位应制定实验废物的安全处置程序，包括：各部门岗位责任、废物的集中分类处置原则、处置方式、排放标准及监测的规定。	**查阅资料**<br>1.查看是否建立实验废物管理程序和制度。<br>2.实验废物处置程序是否具体可操作性。	符合	一般不符合	严重不符合	不适用
二、人员要求	**（四十四）实验废物处置人员：**<br>1.应指定专门的部门和人员承担医疗废物处置工作。<br>2.应对从事实验废物收集、运送、贮存、处置的人员，进行相关法律、法规、标准、安全防护以及紧急处置技术与知识的培训。<br>3.实验室设立单位，应采取有效的职业卫生防护措施，为从事实验废物收集、运送、贮存、处置工作的人员配备必要的防护用品，每年至少进行1次健康检查。<br>4.压力容器操作人员应获得相应资质。	**现场观察**<br>1.查看是否明确指定专门的部门和人员负责处置工作。<br>2.查看是否对各类废物处置人员进行针对性的培训。<br>3.查看各类废物处置时人员是否采取适当的个体防护。<br>4.查看废物处置人员健康检查资料。<br>5.查看压力容器操作人员是否具有相应资质。<br>**询问**<br>询问处置人员是否了解相关制度及处置要求。	符合	一般不符合	严重不符合	不适用
三、实验废物管理	**（四十五）实验废物包装：**<br>1.使用合格的包装材料，包装袋为淡黄色，并有盛装实验废物类型的文字说明及警示标识，感染性废物、病理性废物、损伤性废物、药物性废物及化学性废物不能混合收集，应在包装袋上加注“感染性废物”，“病理性废物”，“药物性废物”字样，必要时加套防渗袋，少量的药物性废物可以混入感染性废物，但应在标签上注明。<br>2.锐器（包括针头、小刀、金属和玻璃）应直接弃置于防穿刺、硬质的合格利器盒内，盒体侧面注明“警告！损伤性废物”，外侧印有警示标识。	**现场观察**<br>1.查看是否是采用专用包装，并具有生物危害标识。<br>2.查看利器盒的材质（不应为PVC）及产品是否有合格证，盒体侧面是否具有生物危害标识。<br>3.查看废物周转箱，是否符合有盖、密封、不渗漏要求。	符合	一般不符合	严重不符合	不适用

表A.5 生物安全实验室管理评价表（实验废物管理）（续）

评价指标	评价内容	评价方法	评价结果			
三、实验废物管理	3.所有的成包（盒）的废物应放置在印有警示标识的周转箱中。周转箱（桶）整体为淡黄色，应具有防滑功能，应防液体渗漏，便于清洗和消毒。<br>4.危险废物应弃置于专门设计的、专用的和有标识的用于处置危险废物的容器内，装量不能超过建议的装载容量，盛装的废物达到包装物或者容器的3/4时，应使用有效的封口方式，使包装物或者容器的封口紧实、严密。	4.现场查看容器内的废物是否存在满溢、洒落；封口方式是否有效。<br>5.查看各类废物是否有混放现象。	符合	一般不符合	严重不符合	不适用
	**（四十六）实验废水排放：**<br>1.实验室污水应经过消毒，并采取合适的消毒工艺流程，不应排放不符合要求的污水。<br>2.不应将固体传染性废物、各种化学废液弃置和倾倒排入下水道。<br>3.各种特殊污水应单独收集并处理后，再排入污水处理站。<br>4.含未经灭活病原微生物废水处理设备宜设在最低处，便于污水收集和检修。	**查阅资料**<br>1.查看排放监测报告，是否均符合相关排放标准。<br>2.查看各类特殊污水（放射性、含重金属）是否按照GB 18466的要求进行排放。<br>3.查看是否有污水消毒记录，是否符合要求。<br>**现场观察**<br>1.查看含未经灭活病原微生物废水处理设备设置位置是否合理。<br>2.查看污水是否分流。固体传染性废物、各种化学废液是否单独收集。	符合	一般不符合	严重不符合	不适用
	**（四十七）实验废物消毒和灭菌：**<br>1.实验废物中病原体的培养基、标本和菌种、毒种保存液高危险废物及含活性高致病性生物因子的废物，在交医疗废物集中处置单位处置前应在产生地点进行灭菌或消毒。	**查阅资料**<br>1.查看是否有消毒与灭菌效果监测记录。<br>2.查看是否有消毒液配置记录。	符合	一般不符合	严重不符合	不适用

表A.5　生物安全实验室管理评价表（实验废物管理）（续）

评价指标	评价内容	评价方法	评价结果			
三、实验废物管理	2.实验室根据菌（毒）种、生物样本及其他感染性材料和污染物，可选用压力蒸汽灭菌方法或有效的化学消毒剂处理。实验室按规定要求做好消毒与灭菌效果监测。<br>3.消毒液应监测其浓度，应标注配制日期、有效期及配制人。	**现场观察**<br>1.查看感染性废物是否选用适当的方法就地消毒或灭菌，是否有相关记录。<br>2.查看压力蒸汽灭菌器或其他消毒设施是否满足消毒和灭菌要求。<br>3.查看灭菌记录、消毒记录是否与被处理的物品相匹配。	符合	一般不符合	严重不符合	不适用
	**（四十八）实验废物存放：**<br>1.废物产生地点应有废物分类收集方法的示意图或者文字说明。<br>2.不应积存垃圾和实验室废物。在消毒灭菌或最终处置之前，应存放在指定的安全地方。<br>3.应建立实验废物暂时贮存设施、设备，不应露天存放；实验废物暂时贮存的时间不应超过2天。<br>4.实验废物暂时贮存设施应远离医疗区、食品加工区、人员活动区和生活垃圾存放场所，方便实验废物运送人员及运送工具、车辆的出入；有严密的封闭措施，设专（兼）职人员管理，防止非工作人员接触实验废物；有防鼠、防蚊蝇、防蟑螂的安全措施；防止渗漏和雨水冲刷；易于清洁和消毒；避免阳光直射；设有明显的警示标识。<br>5.暂时贮存病理性废物，应具备低温贮存或者防腐条件。<br>6.应对实验废物暂时贮存地点，设施进行清洁和消毒处理。	**现场观察**<br>1.查看是否有废物分类收集说明。<br>2.查看实验室内有无废物长时间积存，未定时转运情况。<br>3.查看有无暂时贮存点，看暂时贮存点位置设立是否合理，是否做好防虫、防鼠、防蝇措施。<br>4.查看废物暂时贮存有无专人管理，是否避免阳光直射，是否有警示标识。<br>5.查看暂时贮存病理性或动物尸体生物废物是否具备低温贮存或者防腐条件。<br>6.查看是否对储存点进行清洁与消毒，是否保持清洁。	符合	一般不符合	严重不符合	不适用
	**（四十九）实验废物转运、交接与记录：**<br>1.运送人员从实验废物产生地点将分类包装的实验废物按照规定的时间和路线运送至指定的暂时贮存地点。运送实验废物应使用防渗漏、防溢洒、无锐利边角、易于装卸和清洁的专用运送工具。	**现场观察**<br>1.查看转运路线、时间、转运工具、防护措施是否符合要求。<br>2.查看暂时贮存地点内废物是否有标识和标签，封口是否符合要求。	符合	一般不符合	严重不符合	不适用

表A.5　生物安全实验室管理评价表（实验废物管理）（续）

评价指标	评价内容	评价方法	评价结果			
三、实验废物管理	2.运送人员在运送实验废物前，应检查包装物或者容器的标识、标签及封口，不符合要求的实验废物运送不应至暂时贮存地点。<br>3.运送人员在运送实验废物时，应防止造成包装物或容器破损和实验废物的流失、泄漏和扩散，并防止实验废物直接接触身体。<br>4.生物感染性废物的最终处置应交由经当地环保部门资质认定的医疗废物处理单位集中处置，按照危险废物转移联单制度填写和保存转移联单。<br>5.应做好内部运送及外部转运的交接记录。包括实验废物的来源、种类、重量或者数量、交接时间、最终去向以及经办人签名项目。登记资料至少保存 3 年。	3.查看暂时贮存地点内是否有废物容器，是否有破损、泄露、洒落现象。<br>4.查看废物处理单位资质文件是否符合要求。<br>5.查看内部转运交接手续、外部交接记录和危险废物转移联单信息是否齐全。<br>6.查看记录是否可追溯，是否归档。	符合	一般不符合	严重不符合	不适用
	**（五十）化学性废物处置：**<br>1.危险化学品的处置应按照《废弃危险化学品污染环境防治办法》规定执行。<br>2.应根据化学废物的不同特性和危险程度分类处置。<br>3.盛装废弃危险化学品的容器和受废弃危险化学品污染的包装物，应按照危险废物进行管理。<br>4.危险化学品包装物、容器的材质以及危险化学品包装的型式、规格、方法和单件质量（重量），应与所包装的危险化学品的性质和用途相适应。<br>5.过期的化学试剂以及相应的溶液应考虑集中后由有资质的企业上门收取后进行统一处理。<br>6.转移危险化学品废物的，应填写危险废物转移联单。<br>7.应对化学废物的来源、处置方式、数量进行记录，以便溯源。	**查阅资料**<br>1.查看化学废物是否分类存放和处置。<br>2.查看是否选择正确的处理方法。<br>3.查看是否有处理记录。<br>4.查看是否应与有资质的处置机构签约处置化学废物的协议。<br>5.查看危险废物转移联单填写的信息是否齐全。<br>6.查看化学废物处置、交接记录是否可追溯。<br>**现场观察**<br>1.查看是否配备存放各类化学废物的容器，是否对化学废物进行分类收集，包装是否贴有明确的标签。<br>2.查看化学废物包装是否严密，是否送单位中转站或收集点；是否清运化学废物，是否有大量堆放现象。<br>3.查看是否存在化学废物和其他（生活）垃圾混放，是否向下水道倾倒化学废液。	符合	一般不符合	严重不符合	不适用

A.6 生物安全实验室管理评价表（实验室内务及实验材料、标识管理）应符合表A.6的要求。

表A.6 生物安全实验室管理评价表（实验室内务及实验材料、标识管理）

评价指标	评价内容	评价方法	评价结果			
一、管理程序	**（五十一）内务管理程序：**<br>实验室设立单位应制定实验室内务管理工作程序。	**查阅资料**<br>查看实验室设立单位是否制定内务管理程序内容是否包括消毒程序、选择、购买、采集、接收、查验、使用、处置和存储实验室材料（包括外部服务）的政策和程序、生物安全标识管理程序。	符合	一般不符合	严重不符合	不适用
二、管理要求	**（五十二）内务管理：**<br>1.实验室应指定专人负责内务工作。<br>2.实验室应保持工作区整洁有序，工作台面、实验室地面、设备表面应保持清洁，规范个人行为，不应从事与实验无关的活动，不应存放与实验活动无关的物品。<br>3.不应混用不同核心工作间的工作程序和个人防护装备，防止人员感染和交叉污染。<br>4.冷藏设备内物品分类存放，避免交叉污染。<br>5.应做好实验室内部消毒，确保消毒液的有效使用，监测其浓度，标注配制日期、有效期及配制人。<br>6.移动式生物安全实验室内外部应整洁有序，符合生物安全要求。	**查阅资料**<br>查看是否指定专人负责。<br>**现场观察**<br>1.查看实验工作区域是否整洁，是否存在和实验无关的活动和物品。<br>2.查看不同核心工作间的个人防护装备是否混用。<br>3.查看冷藏设备内物品存放是否整洁，试剂耗材与样本是否存放在同一区域。<br>4.查看实验室消毒剂配制、实施人员。<br>5.查看人员是否配备适用的个体防护装备。<br>6.查看移动式实验室内外部是否整洁有序。	符合	一般不符合	严重不符合	不适用
三、消毒和灭菌管理	**（五十三）消毒和灭菌：**<br>1.实验室应根据操作的病原微生物种类、污染的对象和污染程度选择适宜的消毒和灭菌方法，选用的消毒剂、消毒器械应符合规定。<br>2.实验室应按照内务管理程序要求进行清洁和消毒，包括手消毒、工作台面消毒、实验空间消毒、设备的去污染。<br>3.实验使用过的防护服、一次性口罩、手套应选用压力蒸汽灭菌方法处理后再按相关实验室废物处置方法处理。	**现场观察**<br>1.查看实验室消毒方法是否与所开展的实验活动相适应，查看选用的消毒剂、消毒器械是否符合规定。<br>2.查看是否按照规定实施清洁和消毒。<br>3.查看防护服、一次性口罩等防护用品消毒是否符合要求	符合	一般不符合	严重不符合	不适用

表A.6　生物安全实验室管理评价表（实验室内务及实验材料、标识管理）（续）

评价指标	评价内容	评价方法	评价结果			
三、消毒和灭菌管理	4.实验仪器设备污染后可用消毒液擦拭消毒。生物安全柜、工作台面在每次实验前后可用消毒液擦拭消毒，必要时，可用环氧乙烷、过氧化氢等消毒。<br>5.污染地面可用消毒剂喷洒或擦拭消毒处理。<br>6.感染性物质溢洒后，应立即使用有效消毒剂处理。<br>7.实验人员需要进行手消毒时，应使用消毒剂擦拭或浸泡消毒，再用洗手液洗手、流水冲洗。<br>8.实施消毒的工作人员应穿戴个体防护装备。	4.查看实验仪器、生物安全柜、工作台面是否按照规定进行消毒。<br>5.查看实验人员手消设施是否完备、齐全。<br>6.查看消毒液是否标注配制日期、有效期及配制人信息。<br>7.查看实施消毒的工作人员是否佩戴个体防护装备。	符合	一般不符合	严重不符合	不适用
四、实验材料管理	**（五十四）实验材料管理：**<br>1.应对实验材料供应商进行评价，并保存评价记录和允许使用的供应商名单。<br>2.应确保实验材料经过性能验证后投入使用，保存相关活动记录。	**查阅资料**<br>1.查看是否建立或编制合格供应商名录，是否对供应商进行评价，并保存记录。<br>2.查看试剂、耗材实验材料的性能验证记录。	符合	一般不符合	严重不符合	不适用
五、标识管理	**（五十五）生物安全标识：**<br>1.实验区域内使用的标识应分成禁止、警告、指令、提示、专用五类，应清楚标示出具体危险材料、危险区域。<br>2.实验室用于标示危险区、警示、指示、证明的图文标识是管理体系文件的一部分，包括用于特殊情况下的临时标识，如“污染”、“消毒中”、“已消毒”、“未消毒”、“设备检修”。<br>3.实验室管理层应每年至少对实验室标识进行1次检查并记录。<br>4.标识应张贴在与安全有关的醒目位置，不应设在可移动的门、窗、架物体上。<br>5.实验室入口处标识应采用生物危险专用标识。标识应明确说明生物防护级别、操作的致病性生物因子、实验室负责人姓名、紧急联络方式和国际通用的生物危险符号。	**查阅资料**<br>查看实验室是否正确使用标识系统，查看所用标识是否在体系文件中有所描述。<br>**现场观察**<br>1.现场查看是否有实验室标识系统的评审记录，是否维护、更新，是否每年检查1次。<br>2.查看标识张贴位置是否合适、醒目，是否设在可移动的门、窗、架物体上。<br>3.查看实验室入口处是否张贴生物安全专用标识，标识的信息是否完整。	符合	一般不符合	严重不符合	不适用

表A.6　生物安全实验室管理评价表（实验室内务及实验材料、标识管理）（续）

评价指标	评价内容	评价方法	评价结果			
五、标识管理	6.实验室出口标识实验室应设计紧急撤离路线，紧急（疏散）出口处应有明显的标识，紧急（疏散）出口和紧急撤离路线标识应在无照明的情况下也可清楚识别。<br>7.实验状态提示标识：必要时，房间的入口处应有警示和进入限制（如正在操作危险材料时），适用时，实验室工作过程中提示工作状态时应使用“工作中”等状态提示标识。<br>8.设备标识：应在设备显著部位标示其唯一编号、校准或验证日期、下次校准或验证日期、正常使用、暂停使用、停止使用状态。<br>9.实验废物标识：废物暂存处、盛装废物的包装物、容器外表面应有废物警示标识，在每个包装物、容器上应有中文标签，中文标签的内容应包括：废物产生单位和部门、产生日期、类别及需要的特别说明，并可追溯。<br>10.危险化学品废物标识：危险废物的容器和包装物以及收集、贮存、运输、处置危险废物的设施、场所，应设置危险废物警告标识。危险废物标签应包括：主要成分、化学名称、危险情况、安全措施、危险废物类别图标和废物产生单位。<br>11.实验室的所有管道和线路应有明确、醒目和易区分的标识，关键设施设备的操作开关如有被误操作可能的，宜明确功能指示标识。<br>12.移动式实验室实验期间，核心实验间入口处的显著位置应有国际通用的生物危害警告标识和相关信息，标识可以采用非固定的方式设置，如挂牌。<br>13.生物安全标识规格设置及材质应符合WS 589要求。	4.查看实验室出口处紧急（疏散）标识能否正常使用，并清晰识别。<br>5.查看实验室有无工作状态提示标识，如在进行终末消毒时是否设置禁止进入警示标识。<br>6.查看仪器设备尤其是生物安全柜、高压蒸汽灭菌器关键防护设备编号、使用状态、生物安全、检定、检测，是否正确标识，且信息完整。<br>7.查看废物包装、容器外表面是否有标识和标签，并有可追溯的信息。<br>8.查看化学废物是否配置统一的标签，信息是否齐全，中转站或收集点是否张贴规范的危险废物警告标识。<br>9.查看各类管道和线路是否有标识。<br>10.查看移动式实验室是否按照规定张贴标识。	符合	一般不符合	严重不符合	不适用

A.7　生物安全实验室管理评价表（消防安保管理）应符合表A.7的要求。

表A.7　生物安全实验室管理评价表（消防安保管理）

评价指标	评价内容	评价方法	评价结果			
一、管理程序	**（五十六）管理程序要求：**<br>1.实验室设立单位应有消防相关的制度和程序，并使所有人员理解。<br>2.实验室设立单位应建立安全保卫的相关程序，确保生物安全实验室的安全。<br>3.实验室设立单位应建立保密程序，以确保患者信息、样本信息、相关检测数据结果不泄漏。	**查阅资料**<br>查看实验室设立单位是否制定消防安全管理程序、安全保卫程序和保密程序。	符合	一般不符合	严重不符合	不适用
二、消防管理	**（五十七）消防培训和演练：**<br>1.应配备适用的应急器材，如消防器材、意外事故处理器材、急救器材。需要时，实验室应使用防爆设施和设备。需要时配备用于扑灭可控制的火情及帮助人员从火场撤离。<br>2.应依据实验室可能失火的类型配置适当的灭火器材并每月维护。<br>3.单位应制定年度消防计划，内容至少包括（不限于）：对实验室人员的消防指导和培训，内容至少包括火险的识别和判断、减少火险的良好操作规程、失火时应采取的全部行动。<br>4.单位每年至少对实验室消防设施设备和报警系统状态的检查进行 1 次检查。<br>5.单位应按照要求进行消防知识培训，每年至少 1 次组织演练。<br>6.如果发生火警，应立即寻求消防部门的援助，并告知实验室内存在的危险。	**查阅资料**<br>1.查看消防器材配备情况是否满足需要，特定场所是否安装防爆设施和设备。查看是否有消防装备维护和巡查记录。<br>2.查看灭火器材是否符合实验室可能失火的类型，并对其进行每月 1 次维护。<br>3.查看年度消防计划，内容是否齐全。<br>4.是否按照规定期限组织检查。<br>5.查看培训和演练签到表及记录，是否覆盖所有相关人员。<br>6.查看是否具有必要的指示标识和火警电话，应急预案是否有相关规定或相关记录。	符合	一般不符合	严重不符合	不适用

表A.7　生物安全实验室管理评价表（消防安保管理）（续）

评价指标	评价内容	评价方法	评价结果			
二、消防管理	**（五十八）高压气体、可燃气体和液体的管理：**<br>1.在实验室内应尽量减少可燃气体和液体的存放量。贮存量应符合国家相关的规定和标准。可燃气体或液体应存放在经批准的贮藏柜或库中，必要时配备气体泄露报警装置。<br>2.应在适用的排风罩或排风柜中操作可燃气体或液体。<br>3.应将可燃气体或液体放置在远离热源或打火源之处，避免阳光直射。<br>4.输送可燃气体或液体的管道应安装紧急关闭阀。<br>5.应配备控制可燃物少量泄漏工具包，如发生明显泄漏，应立即寻求消防部门的援助。<br>6.需要冷藏的可燃液体应存放在防爆（无火花）的冰箱中。<br>7.供气（液）罐应放在实验室防护区外易更换和维护的位置，安装牢固，不应将不相容的气体或液体放在一起。<br>8.真空装置应有防止真空装置的内部被污染的措施；不应将真空装置安装在实验场所之外。	**现场观察**<br>1.查看易燃易爆气体是否配备气体泄露报警装置、防爆柜、排气扇等。<br>2.查看实验室内可燃气体和液体的存放是否过量，存放位置是否安全。可燃气体或液体有无存放在经批准的贮藏柜或库中，必要时使用防爆柜，是否配备气体泄露报警装置。<br>3.查看是否在排风罩或排风柜中操作可燃气体或液体。<br>4.可燃气体或液体是否远离热源或打火源之处，避免阳光直射。<br>5.查看输送可燃气体或液体管道有无安装紧急关闭阀。<br>6.查看是否配备控制可燃物少量泄漏的工具包。<br>7.查看需要冷藏的可燃液体是否存放在防爆（无火花）的冰箱中。<br>8.查看管道是否漏气，牢固，破损。是否有截止阀、防回流装置或 HEPA 过滤器。<br>9.查看供气（液）罐放置区域，是否牢固，是否有混放。<br>10.真空干燥装置是否放在实验场所内。	符合	一般不符合	严重不符合	不适用
三、安全保卫	**（五十九）实验室安全保卫：**<br>1.实验室设立单位应落实安全责任部门和专人实验室安全保卫进行管理，并落实安全保卫措施。<br>2.实验室应采取相应的物理防范措施，避免生物材料、样本、药品、化学品和机密资料被误用、被偷盗和被不正当使用，必要时，应设门禁系统。<br>3.实验室设立单位应做好各类人员准入的授权管理工作。	**查阅资料**<br>1.查看单位管理部门及安保人员措施是否落实到位。<br>2.查看相关管理记录，人员准入授权记录。<br>**现场观察**<br>1.查看实验室是否具有安全保卫措施，实验室主入口和重点部位有无监控。<br>2.查看是否采取足够的物理措施避免危险材料被误用、偷盗和	符合	一般不符合	严重不符合	不适用

表A.7 生物安全实验室管理评价表（消防安保管理）（续）

评价指标	评价内容	评价方法	评价结果			
三、安全保卫	4.根据保存的危险材料风险高低和实验室周围情况安装必要的安全防护和监控设施。<br>5.应加强对高致病性病原微生物菌（毒）种和生物样本的安全管理与控制，实行双人双锁，有人员进出监控、报警措施。应对从事高致病性病原微生物活动的实验室的核心工作区域加装视频监控，并接入监控室。监控设备应清晰可见（建议1080p）和影像存储容量，存储时间不少于3个月。高致病性病原微生物菌(毒)种或生物样本运输应确保安全，应有防止样本丢失、被偷被抢后的安全防护措施。<br>6.若使用放射性和危化品等物质应配备相应的安全设施、设备和个体防护装备，应符合国家、地方的相关规定和要求。<br>7.菌(毒)种或生物样本保存区域应有消防、防盗、监控、报警、通风和温湿度监测与控制设施；保存设备应有防盗和温度监测与安全控制设施条件。<br>8.对可能导致甲类传染病传播的以及国务院卫生行政主管部门规定的菌种、毒种和传染病检测样本，确需采集、保藏、携带、运输和使用的，应经省级以上人民政府卫生行政主管部门批准。	不正当使用，查看实验室是否设置门禁系统物理控制措施。<br>3.查看实验室的周围区域是否具有安全防护措施和监控设施。<br>4.查看高致病性病原微生物菌（毒）种和生物样本的管理是否执行“双人双锁”。查看从事高致病性病原微生物活动的实验室的核心工作区是否具有监控，并接入监控室。查看监控设备是否具有足够的分辨率和储存容量，监控系统是否备份相关数据。<br>5.查看菌(毒)种或生物样本保存区域的消防、防盗、监控、报警、通风和温湿度监测与控制设施能否满足要求。<br>6.查看实验室设立单位是否落实专门的部门和人员具体负责菌（毒）种和生物样本管理。<br>7.查看甲类传染病传播鼠疫、霍乱和参照甲类管理的病原菌采集、保藏、携带、运输和使用是否经过批准。<br>**询问**<br>询问运送人员在运输高致病性病原微生物菌(毒)种或生物样本时是否采取相应的安全措施，观察转运箱是否有锁。	符合	一般不符合	严重不符合	不适用
四、保密管理	**（六十）信息保密管理：**<br>1.实验室设立单位应建立实验室生物安全保密制度，落实专门部门和人员负责保密管理，重点落实高致病性病原微生物菌毒种和生物样本信息（包括菌（毒）种和生物样本信息、患者信息、检测结果、测序结果）保密措施，防止信息泄露。<br>2.实验室设立单位管理层应掌握本单位的菌（毒）种和生物样本的全部信息（包括运输、使用、保存、销毁）。	**现场观察**<br>1.查看实验室设立单位是否建立信息保密制度，是否落实专门部门人员负责，有效措施确保安全。<br>2.查看实验室设立单位是否建立本单位菌（毒）种和生物样本的清单，并能确保信息安全。<br>3.查看设立单位管理部门是否采取有效的监督管理。	符合	一般不符合	严重不符合	不适用

表A.7　生物安全实验室管理评价表（消防安保管理）（续）

评价指标	评价内容	评价方法	评价结果			
四、保密管理	3.实验室设立单位任何个人不应泄露本单位与实验室生物安全相关的保密信息。	4.查看高致病性病原微生物菌（毒）种或感染性样本的信息保存是否存在安全风险。	符合	一般不符合	严重不符合	不适用
四、保密管理	**（六十）信息保密管理：**<br>1.实验室设立单位应建立实验室生物安全保密制度，落实专门部门和人员负责保密管理，重点落实高致病性病原微生物菌毒种和生物样本信息（包括菌（毒）种和生物样本信息、患者信息、检测结果、测序结果）保密措施，防止信息泄露。<br>2.实验室设立单位管理层应掌握本单位的菌（毒）种和生物样本的全部信息（包括运输、使用、保存、销毁）。<br>3.实验室设立单位任何个人不应泄露本单位与实验室生物安全相关的保密信息。<br>4.实验室设立单位管理部门应对保密管理采取有效的监督管理。<br>5.高致病性病原微生物菌（毒）种或感染性样本的保存、运输应符合保密要求。	**现场观察**<br>1.查看实验室设立单位是否建立信息保密制度，是否落实专门部门人员负责，有效措施确保安全。<br>2.查看实验室设立单位是否建立本单位菌（毒）种和生物样本的清单，并能确保信息安全。<br>3.查看设立单位管理部门是否采取有效的监督管理。<br>4.查看高致病性病原微生物菌（毒）种或感染性样本的信息保存是否存在安全风险。<br>**询问**<br>1.询问实验室设立单位如何开展病原微生物相关信息的保密管理。<br>2.询问是否有信息泄露的情况。	符合	一般不符合	严重不符合	不适用

A.8　生物安全实验室管理评价表（其他日常管理）应符合表A.8的要求。

表A.8　生物安全实验室管理评价表（其他日常管理）

评价指标	评价内容	评价方法	评价结果			
一、安全计划	**（六十一）安全计划要求：**<br>1.实验室设立单位应按照 GB 19489 要求在年初制定生物安全年度工作计划，并经管理层（法定代表人或单位生物安全负责人）批准后发布。	**查阅资料**<br>1.查看是否制定生物安全年度工作计划。<br>2.查看生物安全年度工作计划是否明确工作任务和责任部门。	符合	一般不符合	严重不符合	不适用

表A.8 生物安全实验室管理评价表（其他日常管理）（续）

评价指标	评价内容	评价方法	评价结果			
一、安全计划	2.生物安全年度工作计划应明确年度工作任务、责任部门、完成时间和目标。<br>3.实验室和相关部门应按时完成年度工作任务，并保存相关记录。	3.查看生物安全年度工作计划是否按时完成和记录。	符合	一般不符合	严重不符合	不适用
二、内部审核	**（六十二）内部审核要求：**<br>1.实验室设立单位应建立内部审核程序。<br>2.单位生物安全负责人应按不大于 12 个月的周期组织、策划管理体系内部审核。<br>3.单位生物安全管理部门应制定详细的内审计划，包括内审员、分工、时间安排、内审依据、内审范围，并由单位生物安全负责人签发。<br>4.应按照内审计划实施现场内部审核。在实施现场审核前内审员应编制详细的内审核查表。<br>5.内审应发现不符合项，不符合项应有被审核部门签字确认并落实纠正措施，对纠正措施实施情况应验证。<br>6.内部审核应形成内审报告，并发放至相关部门。	**查阅资料**<br>1.查看程序文件是否规定对所有管理要素和技术要素进行内部审核。<br>2.查看最近两次内审间隔时间是否在 12 个月内。<br>3.查看内部审核计划内容是否符合要求并经单位生物安全负责人签发。<br>4.查看内审核查表，内审表内容是否详细，包括被审部门、审核内容、审核方法、审核结果。<br>5.查看内审是否发现不符合项工作；对内审中发现的不符合项是否提出了明确的纠正措施，是否规定了完成期限。<br>6.查看是否在规定的时间内完成不符合项的整改，是否验证纠正措施的有效性。<br>7.查看内部审核报告内容是否包括发现的不符合项和整改要求；内部审核报告是否发放至相关部门。	符合	一般不符合	严重不符合	不适用
三、管理评审	**（六十三）管理评审要求：**<br>1.实验室设立单位应建立管理评审程序。<br>2.应按不大于 12 个月的周期对管理体系进行管理评审。<br>3.管理评审应由管理层（一般是法定代表人）负责组织并实施（管理评审一般是会议形式举行）。<br>4.单位生物安全管理部门应制定管理评审实施计划。	**查阅资料**<br>1.查看管理体系是否包含管理评审程序。<br>2.查看最近 2 次管理评审时间是否在 12 个月内。<br>3.查看单位生物安全管理部门是否下达管理评审通知和计划，是否明确了参加的具体部门和汇报的材料。<br>4.查看管理评审是否对全部生物安全实验室及相关活动进行评审。	符合	一般不符合	严重不符合	不适用

表A.8　生物安全实验室管理评价表（其他日常管理）（续）

评价指标	评价内容	评价方法	评价结果			
三、管理评审	5.应对实验室安全管理体系及其全部活动进行评审，包括设施设备的状态、人员状态、实验室相关的活动、发生的变更和安全事故等全部要素。<br>6.单位生物安全管理部门应记录管理评审的发现及提出的改进建议。<br>7.实验室管理层应明确改进决议并确保所提出的改进决议在规定的时间内完成。<br>8.应编制管理评审报告。	5.查看管理评审报告，是否对生物安全管理体系的适用性和有效性进行评价；管理评审是否明确成改进决议，<br>6.查看管理评审中提出的改进建议是否明确了责任部门和完成期限，或作为来年的工作目标进行落实。<br>7.查看是否在规定的时间内落实和完成改进决议。	符合	一般不符合	严重不符合	不适用
四、不符合项的识别和控制	**（六十四）不符合项的识别和控制要求：**<br>1.单位应建立不符合项的识别和控制机制。<br>2.当发现有任何不符合生物安全安全管理体系要求或其他不符合项工作时，实验室管理层应采取以下措施：<br>（1）将解决问题的责任落实到个人；<br>（2）明确规定应采取的措施；<br>（3）只要发现很有可能造成感染事件或其他损害，立即终止实验室活动并报告；<br>（4）立即评估危害并采取应急措施；<br>（5）分析产生不符合项的原因和影响范围，并采取补救措施；<br>（6）进行风险评估；<br>（7）采取纠正措施并验证有效；<br>（8）明确规定恢复工作的授权人及责任；<br>（9）记录每一不符合项及其处理过程并形成文件。<br>3.实验室管理层应按规定的周期评审不符合项报告，进行趋势分析和风险分析并采取预防措施。	**查阅资料**<br>1.查看管理体系文件，是否有不符合项的识别和控制要求，是否具有可操作性。<br>2.是否对发现的不符合项进行评估并采取相应的控制措施。<br>3.查看对所有发现的不符合项是否进行评审，是否制定了必要的预防措施。	符合	一般不符合	严重不符合	不适用

表A.8 生物安全实验室管理评价表（其他日常管理）（续）

评价指标	评价内容	评价方法	评价结果			
五、纠正措施	**（六十五）纠正措施要求：**<br>1.实验室设立单位应明确纠正措施要求。<br>2.纠正措施应包括识别问题发生的根本原因的调查要求。<br>3.纠正措施应与问题的严重性及风险的程度相适应。<br>4.实验室管理层应将因纠正措施所致的管理体系的任何改变文件化并实施。<br>5.实验室管理层应负责监督和检查所采取纠正措施的效果。	**查阅资料**<br>1.查看管理体系文件，是否规定了纠正措施要求。<br>2.查看纠正措施是否从发生问题的根本原因分析开始进行调查。<br>3.查看采取的纠正措施是否与问题的严重性及风险的程度相适应。<br>4.若采取的纠正措施需要修订或增加管理体系相关文件，查看是否已经按照要求完成并发放。<br>5.查看纠正措施是否在规定的时间内完成，并进行实施效果验证。	符合	一般不符合	严重不符合	不适用
六、预防措施	**（六十六）预防措施要求：**<br>1.实验室设立单位应明确预防措施要求。<br>2.应识别无论是技术还是管理体系方面的不符合项来源和所需的改进。<br>3.单位管理层应每年至少进行 1 次不符合项风险分析，包括对外部评价的分析。<br>4.预防措施，应制定行动计划、监督和检查实施效果。	**查阅资料**<br>1.查看管理体系文件，是否规定预防措施实施要求。<br>2.查看是否每年至少进行 1 次进行风险分析，并采取必要的预防措施，包括技术和管理体系方面所需的改进。<br>3.查看预防措施是否制定计划。<br>4.查看预防措施是否在规定的时间内完成，并对预防措施实施效果进行评价。	符合	一般不符合	严重不符合	不适用
七、持续改进	**（六十七）持续改进要求：**<br>1.实验室设立单位应建立持续改机制。<br>2.实验室设立单位应每年至少进行 1 次系统地评审管理体系，以识别所有潜在的不符合项来源、识别对管理体系或技术的改进机会。<br>3.应改进识别出的需改进之处，应制定改进方案，如果采取改进措施，应评价其效果。	**查阅资料**<br>1.查看单位管理体系文件中是否明确相关要求。<br>2.查看实验室管理层是否每年至少进行 1 次系统地评审管理体系，是否识别出潜在的不符合项来源，并提出具体的管理体系或技术改进要求或建议。<br>3.查看改进措施是否制定了改进方案或实施要求，是否落实了责任部门，明确完成期限，并对实施效果进行验和评价。	符合	一般不符合	严重不符合	不适用

表A.8　生物安全实验室管理评价表（其他日常管理）（续）

评价指标	评价内容	评价方法	评价结果			
七、持续改进	4.实验室设立单位应保证所有员工参加改进活动，并提供相关的教育和培训机会。	3.查看改进措施是否制定了改进方案或实施要求，是否落实了责任部门，明确完成期限，并对实施效果进行验和评价。<br>4.查看员工提出的合理的改进方案是否落实。	符合	一般不符合	严重不符合	不适用

附 录 B
（规范性）
生物安全实验室管理评价结果登记表

生物安全实验室管理评价结果登记表应符合表B.1的规定。

表B.1 生物安全实验室管理评价结果登记表

<table>
<tr><td>被评价机构名称</td><td colspan="3"></td></tr>
<tr><td>法定代表人姓名</td><td></td><td>联系电话</td><td></td></tr>
<tr><td>单位联系人姓名</td><td></td><td>联系电话</td><td></td></tr>
<tr><td>单位地址</td><td colspan="3"></td></tr>
<tr><td>评价方式</td><td colspan="3">（☐自评 ☐他评）</td></tr>
<tr><td colspan="4">严重不符合项：

不符合项：</td></tr>
<tr><td colspan="4">整改要求：</td></tr>
<tr><td colspan="4">评价组签名：

日期：</td></tr>
<tr><td colspan="4">单位法定代表人（或授权人）签名：

日期：</td></tr>
</table>
