# Supplementary material for: Biosafety status analysis and risk assessment of laboratories from 2021 to 2023 in Jiaxing, China
Source: Front Bioeng Biotechnol. 2025 Apr 16;13:1442651. doi: 10.3389/fbioe.2025.1442651 (PMC12040964; doi:10.3389/fbioe.2025.1442651)
Supplement: Supplementary file 1 [file DataSheet1.zip › supplementary/RBT 040-2020《病原微生物实验室生物安全风险管理指南》.pdf]

ICS 03.120.20
A 00

中华人民共和国认证认可行业标准

RB/T 040—2020

病原微生物实验室生物安全风险管理指南

Guidelines on risk management of biosafety in pathogenic microorganism laboratories

2020-08-26 发布 2020-12-01 实施

国家认证认可监督管理委员会 发布

目　　次

前言 ………………………………………………………………………………………… Ⅲ
1　范围 ……………………………………………………………………………………… 1
2　规范性引用文件 ………………………………………………………………………… 1
3　术语和定义 ……………………………………………………………………………… 1
4　原则 ……………………………………………………………………………………… 3
5　实施过程 ………………………………………………………………………………… 4
5.1　概述 …………………………………………………………………………………… 4
5.2　任务来源 ……………………………………………………………………………… 4
5.3　实施准备 ……………………………………………………………………………… 5
5.4　风险管理实施 ………………………………………………………………………… 6
5.5　再评估 ………………………………………………………………………………… 10
附录A（资料性附录）　实验室生物安全风险评估的常用方法 ………………………… 11
附录B（资料性附录）　实验室生物安全风险评估矩阵 ………………………………… 13
附录C（资料性附录）　病原微生物实验活动风险评估实施参考示例 ………………… 15
参考文献 …………………………………………………………………………………… 24

前　言

本标准按照 GB/T 1.1—2009 给出的规则起草。

本标准由国家认证认可监督管理委员会提出并归口。

本标准起草单位：中国合格评定国家认可中心、中国动物卫生与流行病学中心、中国建筑科学研究院有限公司、军事科学院军事医学研究院、中国疾病预防控制中心、中国科学院武汉病毒研究所、中国农业科学院哈尔滨兽医研究所、中国农业科学院兰州兽医研究所、中国海关科学技术研究中心、中国认证认可协会。

本标准主要起草人：王荣、王君玮、曹国庆、翟培军、周永运、陆兵、赵赤鸿、宋冬林、傅斌友、吴东来、唐江山、谷强、李沐洋、付岳。

病原微生物实验室生物安全风险管理指南

1 范围

本标准给出了病原微生物实验室(以下简称“实验室”)开展生物安全风险管理原则和实施过程的通用指南。

本标准适用于病原微生物实验室开展风险管理工作,也可用于监督管理部门对实验室生物安全风险管理工作的评价和考核。

2 规范性引用文件

下列文件对于本文件的应用是必不可少的。凡是注日期的引用文件,仅注日期的版本适用于本文件。凡是不注日期的引用文件,其最新版本(包括所有的修改单)适用于本文件。

GB 19489—2008 实验室 生物安全通用要求

GB/T 23694—2013 风险管理 术语

WS 233—2017 病原微生物实验室生物安全通用准则

3 术语和定义

GB/T 23694—2013 界定的以及下列术语和定义适用于本文件。为了便于使用,以下重复列出了GB/T 23694—2013 中的某些术语和定义。

3.1

生物因子 biological agents

微生物和生物活性物质。

[GB 19489—2008,定义 2.4]

3.2

风险 risk

不确定性对目标的影响。

注 1:通常用事件后果(包括情形的变化)和事件发生可能性的组合来表示风险。

注 2:改写 GB/T 23694—2013,定义 2.1。

3.3

生物风险 biorisk

与生物因子相关的不确定性对目标的影响。

3.4

风险管理 risk management

在风险方面,指导和控制组织的协调活动。

[GB/T 23694—2013,定义 3.1]

3.5

风险源 risk source

可能单独或共同引发风险的内在要素。

注:风险源可以是有形的,也可以是无形的。

[GB/T 23694—2013,定义 4.5.1.2]

3.6

风险评估　risk assessment

包括风险识别、风险分析和风险评价的全过程。

[GB/T 23694—2013，定义 4.4.1]

3.7

风险识别　risk identification

发现、确认和描述风险的过程。

注 1：风险识别包括对风险源、事件及其原因和潜在后果的识别。

注 2：风险识别可能涉及历史数据、理论分析、专家意见以及利益相关方的需求。

注 3：改写 GB/T 23694—2013，定义 4.5.1。

3.8

风险分析　risk analysis

理解风险性质、确定风险等级的过程。

注 1：风险分析是风险评价和风险应对决策的基础。

注 2：风险分析包括风险估计。

[GB/T 23694—2013，定义 4.6.1]

3.9

风险评价　risk evaluation

对比风险分析结果和风险准则，以确定风险和/或其大小是否可以接受或容忍的过程。

注：风险评价有助于风险应对决策。

[GB/T 23694—2013，定义 4.7.1]

3.10

风险准则　risk criteria

评价风险重要性的依据。

注 1：风险准则的确定需要基于组织的目标、外部环境和内部环境。

注 2：风险准则可以源自标准、法律、政策和其他要求。

[GB/T 23694—2013，定义 4.3.1.3]

3.11

风险等级　level of risk

单一风险或组合风险的大小，以后果和可能性的组合来表达。

[GB/T 23694—2013，定义 4.6.1.8]

3.12

风险应对　risk treatment

处理风险的过程。

注 1：通常指基于风险评估结果，为降低风险而采取的综合性措施。其最终目标是降低事故发生的频率和/或事故的严重程度，使剩余风险可接受。

注 2：改写 GB/T 23694—2013，定义 4.8.1。

3.13

剩余风险　residual risk

风险应对之后仍然存在的风险。

注 1：剩余风险可包括未识别的风险。

注 2：剩余风险还被称为“留存的风险”。

[GB/T 23694—2013，定义 4.8.1.6]

3.14

风险接受 risk acceptance

接受某一特定风险的决定。

注 1：风险接受可以不经风险应对，还可以在风险应对过程中发生。

注 2：接受的风险要受到监督和评审。

[GB/T 23694—2013，定义 4.7.1.6]

3.15

控制 control

处理风险的措施。

注 1：控制包括处理风险的任何流程、策略、设施、操作或其他行动。

注 2：控制并非总能取得预期效果。

[GB/T 23694—2013，定义 4.8.1.1]

3.16

利益相关方 stakeholder

可以影响、被影响或自认为会被某一决策或行动影响的个人或组织。

注 1：决策者可以是利益相关方。

注 2：改写 GB/T 23694—2013，定义 4.2.1.1。

4 原则

风险管理原则是实验室实施风险管理工作的基础，可用于管理生物风险对实验室安全管理目标的影响。实验室应在风险管理规划和实施过程中予以充分考虑。

实验室生物安全风险管理的最终目标是创造和保护价值，即鼓励创新、提高实验室性能和确保实验室安全有序运行。

实验室的风险管理一般应包含 8 个原则。每个原则之间既相互独立，又互为补充，共同为实现实验室风险管理目标提供支撑：

——融合性原则：风险管理是病原微生物实验室开展实验活动不可或缺的组成部分，应融合到实验室管理体系中。

——模式化原则：风险管理宜模式化，以便系统全面地管理风险源，并有助于结果的一致性和可比性。

——个性化原则：应根据实验室特点和实验室内外部环境信息，制定个性化风险管理方案并记录实施过程。

——包容性原则：实验室应与利益相关方及时充分地沟通交流，并将其知识、观点和看法融入风险管理。

——动态性原则：随着内外部环境信息变化，实验室风险也处于动态变化中，表现为新风险的出现、风险等级的变化或消失。实验室应通过风险管理活动及时地预测、识别、确认并应对这些风险。

——信息依赖性原则：实施风险管理应充分认识到实验室当前状况，并利用已有相关信息和实验室未来运行计划。相关信息应能及时、清晰地与利益相关方交流。

——人文因素原则：应考虑个人行为、文化背景等人文因素对实验室不同运行阶段风险管理的影响。

——持续改进原则：实验室应通过内外部审核、安全检查等措施定期评价风险应对的适宜性、充分性和有效性，持续改进风险管理。

5 实施过程

5.1 概述

风险管理的实施过程一般可分为任务来源、实施准备和风险管理实施三个阶段,如图1所示。

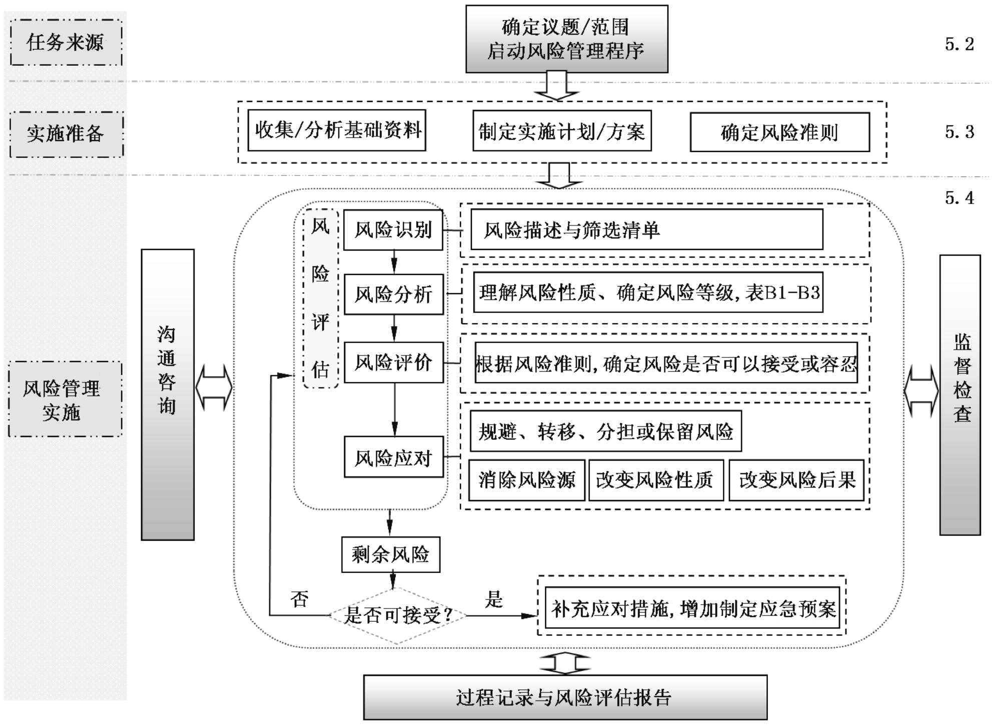

图1 风险管理的基本实施过程

5.2 任务来源

5.2.1 概述

实验室的风险管理应与实验室的运行阶段相适应。风险管理工作应明确实验室风险管理的任务来源,了解风险管理的目标与承诺,充分考虑实验室资源状况,包括但不限于设施设备状态、管理体系状况、实验活动范围和人员配备情况等。

5.2.2 确定范围

实验室应设定风险管理范围,包括但不限于生物因子、实验活动、涉及区域、设施设备、组织机构、人员配置、个人防护等内容。

5.2.3 明确目标和承诺

实验室管理层应通过发布政策、声明或其他形式,明确表达实验室风险管理的目标和对风险管理结果的承诺,包括但不限于:

——风险管理的目标;

——风险管理目标和实验室总体管理目标、承诺和其他政策之间的关系;

——将风险管理纳入实验室文化的一部分；

——将风险管理纳入核心工作范围；

——风险管理涉及的权限、职责和义务；

——提供必要的资源；

——处理冲突的方式；

——实验室绩效指标的评价与应用；

——持续改进。

适用时，应在实验室内部和利益相关方之间对风险管理的目标和承诺进行充分沟通。

5.3 实施准备

5.3.1 基础资料及环境信息

5.3.1.1 基础资料收集

实验室宜收集开展风险管理对象相关的资料，并对其进行分析、梳理，融入管理体系。这些资料包括但不限于：

——国内相关法律法规、部门规章（或部令公告）和标准规范；

——国际组织或行业权威机构发布的指南、预案；

——实验室环境及设施设备等相关信息。

5.3.1.2 明确内外部环境信息

实验室宜充分考虑内外部利益相关方的活动目标和核心关注点，厘清实验室内外部环境信息。

外部环境信息宜包括但不限于：

——国际、国家、区域等不同层面关于实验室生物安全管理的状况；

——影响本实验室风险管理目标和承诺的主要外部因素和趋势；

——外部利益相关方（实验室设计单位、承建单位、运行维护单位、设备供应商以及相关管理部门等）的需求和相互关系；

——与相关方的合同关系和承诺；

——明确实验室周边人群居住或/和动物养殖状况信息，包括易感人群或/和易感动物养殖数量、与实验室的距离等。

内部环境信息宜包括但不限于：

——实验室的愿景、发展目标；

——实验室（独立法人组织）或其母体组织（非独立法人实验室）的要求；

——组织机构（如生物安全管理委员会的设置），实验室与母体组织内部其他相关部门的关系（如：管理交叉、协同等）；

——实验室内部工作的分工（如实验活动操作人员、生物安全管理人员、设施设备运行维护人员等）、职责和权限；

——实验室的文化建设；

——风险管理拟采用的标准、准则和模式；

——实验室资源状况，如：经费来源、人员和团队状况、认证认可体系、技术能力等；

——实验室信息系统、网络资源等；

——内部利益相关方（如实验室基建人员、后勤保障人员、管理层、实验操作人员等）之间的关系，包括理念、价值观、认识水平等；

——合同关系和承诺。

5.3.2 制定实施方案

宜根据5.2.2实验室设定的风险管理范围制定风险管理方案。风险管理方案应涵盖人员分工和职责、时间安排以及监督考核等内容，规定适用的风险评估方法参照附录A、应保存的记录以及与其他项目、过程和活动的关联等。风险管理方案应得到实验室管理层的批准。必要时，还应得到主管部门的批准。

5.3.3 确定风险准则

实验室开展风险评估前应根据生物因子危害程度、后果预期制定风险准则。制定风险准则时，应充分考虑生物因子的危害特性、在国家或地区的流行状况、实验室的可接受程度等要素，对危害程度分级标准、事件发生的可能性大小、后果严重程度判定标准作出定性或定量描述。

风险准则应与实验室风险管理的目标、承诺和政策相一致，充分考虑实验室应承担的风险管理义务以及利益相关方的观点。

风险准则是动态的，可以根据实验室操作生物因子变化、实验活动内容改变以及实验室对生物安全管理的目标和承诺进行调整。必要时，应对实验室确定的风险准则进行持续审查和适时修改。制定风险准则时，应考虑但不限于以下因素：

——影响结果和目标的不确定性的性质和类型，如生物因子已知或未知的危害程度；

——时间相关因素；

——地域相关因素；

——风险发生的可能性和发生后果严重性分级；

——风险等级的划分原则；

——多重风险的叠加和相互影响；

——实验室的能力水平。

5.4 风险管理实施

5.4.1 沟通咨询

5.4.1.1 实验室应建立良好的沟通和咨询机制，确保沟通和咨询贯穿于风险管理的全过程。沟通包括与利益相关方分享信息，就可能存在的分歧达成一致。咨询除了化解或消除疑惑外，还包括对风险预期的反馈，以支持决策或实施进一步风险管理活动。沟通和咨询的方式、方法和内容应能充分反映利益相关方的预期。

5.4.1.2 在明确信息过程中，实验室应与利益相关方进行充分沟通，充分获得相关信息。实验室风险评估完成后，还应针对风险评估结果与政策制定者、决策者以及涉及实验室生物安全的管理部门等机构进行充分交流，以便有效实施风险控制。

5.4.1.3 沟通和咨询应及时、有效，保证相关信息的收集、整理、分析和共享，并提供及时反馈，必要时对沟通、咨询方式方法做出调整和改进。

5.4.2 风险评估

5.4.2.1 风险识别

风险识别应对实验活动中涉及的风险源进行逐一识别，并对其特性进行定性描述，生成风险清单或风险列表。

风险识别应考虑但不限于以下要素：

——本项实验活动涉及生物因子的已知或未知特性，包括危害程度分类、生物学特性、在环境中的

稳定性、传播途径、易感性和致病性、宿主范围、最低感染剂量、潜伏期、临床表现以及治疗和预防措施等。此外，还应考虑该生物因子与其他生物体和环境的相互作用，相关实验数据和流行病学资料；

——常规实验活动，如：样品处理、病原（病毒、细菌、真菌等）分离培养与鉴定、实验操作（如：离心、研磨、振荡、匀浆、超声破碎、冷冻干燥等）、器具（如：玻璃器皿、剪刀、针头、移液器等）的使用，等等；

——非常规实验活动，如：操作超常规样品数量的检测工作、超常规量的大量病毒或细菌培养，或者进行新的实验活动；设施设备维修维护活动；外来人员进入实验室的活动；

——实验活动涉及遗传修饰生物体时，新的重组体可能引起的危害；

——涉及致病性生物因子的动物饲养与动物实验活动；

——感染性废物处置过程中的风险；

——实验活动管理带来的次生风险，如：采取风险控制措施后的残余风险或带来的新风险，操作规程不符合要求产生的潜在风险等；

——涉及致病性生物因子实验活动的人员相关的风险，如：专业知识背景、操作熟练程度、生物安全意识或对风险的认知、接受的培训程度、健康状况、心理素质以及可能影响工作的压力等；

——设施设备相关的风险；

——实验室生物安保制度和安保措施，如：因安保措施不当导致的致病性生物因子被盗、恶意使用带来的风险；

——国内外已发生的实验室感染事件原因分析；

——必要时，化学、物理、电气、火灾、水灾、自然灾害等的风险。

5.4.2.2 风险分析

实验室应对风险涉及事件发生的可能性及其后果的严重性进行分析，并据此确定风险等级。实验室应采用适当方法参见附录 A，描述事件发生的可能性和后果严重性参见附录 B。实验室的风险等级可以根据事件发生的可能性和后果的严重性综合判定，一般分为低、中、高、极高四个级别，可参照表B.3确定。

5.4.2.3 风险评价

实验室应根据风险分析结果，对照风险准则，根据自身实际情况判定风险是否可接受。当风险可接受时，应保持已有的安全措施；当风险不可接受时，应采取风险应对措施以消除、降低或控制风险。

对于新识别的风险，实验室应及时修订补充相应的风险准则，以便在风险评估中适时做出风险评价。

5.4.3 风险应对

5.4.3.1 概述

风险应对是选择并执行一种或多种改变风险的措施，包括改变风险事件发生的可能性或后果的措施。对于风险应对措施，应评估其剩余风险是否可以承受。如果剩余风险不可承受，应调整或制定新的风险应对措施，并评估新的风险应对措施的效果，直到剩余风险可以承受。执行风险应对措施会引起实验室风险的改变。实验室应跟踪、监督、评价风险应对的效果，并对变化的风险进行及时评估。必要时，重新制定风险应对措施。

GB 19489—2008 和 WS 233—2017 中有关风险控制的要求，适用于本标准风险应对措施范畴。实验室的风险应对措施，一般包括但不限于：

——停止具有风险的实验活动，以规避风险；
——消除具有负面影响的风险源；
——降低风险事件发生的可能性及其分布；
——改变风险事件发生后可能导致的后果严重程度；
——将风险转移到其他区域或范围；
——保留并承担风险。

5.4.3.2 选择风险应对措施

选择风险应对措施时，应考虑的因素包括但不限于：
——法律法规、标准规范方面的要求；
——风险应对措施的实施成本与预期效果；
——选择几种应对措施，将其单独或组合使用；
——利益相关方的诉求、对风险的认知和承受度，以及对某些风险应对措施的偏好。

风险应对措施宜遵循的基本原则包括但不限于：
——全过程控制原则：全面的风险控制一般需要多个输入、输出过程的有机集合，最终达到预期目标，其中信息反馈和控制措施则是保证输出结果的重要环节；
——动态控制原则：实验室由于实验活动或运行阶段的不同，风险也会随之发生变化。应充分考虑风险的动态变化特性，根据实验室运行和实验活动变化的实际情况，随时识别风险并确定风险关键控制点，以便适时、正确地实施风险控制；
——分级控制原则：根据实验室的组织结构和风险本身的规律，采取分级控制的原则，使得目标分解、责任分明，最终实现完整控制；
——分层控制原则：根据实验室的特点和风险特征，可以通过根本的预防性控制、补充性控制、防止事故扩大的预防性控制、维护性能的控制、经常性控制以及紧急性控制等不同层次，来提高控制效率并增加风险控制的可靠程度。

可采取 5.4.3.1 叙述的风险消除、降低、控制或转移等方法，保证风险控制措施有效。风险应对措施在实施过程中可能无法满足所有风险的控制要求，应把监督和检查作为风险应对措施计划的有机组成部分，保证应对措施持续有效。

实验室管理层和其他利益相关方应清楚在采取风险应对措施后剩余风险的性质和程度。

5.4.3.3 制定风险应对计划

实验室应根据选择的风险应对措施制定相应的风险应对计划，一般应包括但不限于：
——实施风险应对措施的人员安排，明确责任人和职责；
——风险应对措施涉及的区域、实验室和实验活动；
——选择多种风险应对措施时，实施风险应对措施的优先次序；
——对报告和监督检查的要求；
——与利益相关方的沟通安排；
——资源需求，包括应急机制等；
——执行时间表。

5.4.4 监督检查和持续改进

5.4.4.1 实验室应建立风险管理活动的监督检查和持续改进的工作机制，以确保相关要求得到及时有效地实施。

5.4.4.2 风险管理的监督和检查计划应列入实验室年度安全计划中。

5.4.4.3 实验室应指定熟悉开展实验活动的专门机构或者人员承担风险管理工作，定期检查相关法律法规、规章制度的实施落实情况。

5.4.4.4 实验室管理层应持续监督和检查所采取风险应对措施的效果，以确保这些措施能有效降低识别出的风险。持续监测（监控）和调整风险管理状况，以应对内外部环境变化，提高实验室安全管理能力。

5.4.4.5 实验室应结合日常监督检查、内外部审核和管理评审，对实施的风险管理工作质量和效果进行定期审核和评价。对识别出的问题，及时组织人员进行原因分析，制定纠正措施，以便持续改进。

5.4.5 过程记录与风险评估报告

5.4.5.1 记录

实验室应有记录风险管理全过程的机制。风险管理全过程的记录应满足 GB 19489—2008 中 3.1.4，7.4.6 的要求。风险管理的记录应包括但不限于：

——风险评估时间以及参加人员的识别信息；

——风险评估的依据，如法律、法规、权威资料、数据等；

——风险评估方法、程序；

——必要时，因评估需要进行的验证信息；

——风险评估报告的编写、审核、批准信息。

风险管理记录的保存期应满足相关法律法规和实验活动可追溯的要求。从事高致病性病原微生物生物安全风险评估的记录保存期一般不少于 20 年。

5.4.5.2 风险评估报告

实验室风险管理完整的实施过程示例参见附录 C，应以编制风险评估报告作为收尾。实验室应充分认识到拟开展的实验活动和程序，并在与利益相关方充分沟通、交流和咨询的基础上编制风险评估报告。风险评估报告的内容应至少包括：

——风险评估报告名称；

——评估参加人员；

——评估范围；

——评估目的；

——评估依据；

——评估方法和程序；

——评估内容；

——讨论过程；

——评估结论。

风险评估报告应满足以下要求，包括但不限于：

——适合自身实验室风险控制的需要；

——能回答实验室相关方，包括主管机构、周边居民、实验室管理人员、实验人员以及来访人员等共同关心的问题；

——系统、科学、实用，必要时采用统计表、图形等直观的方法表示；

——有风险等级表述以及风险是否可控的依据和结论；

——明确风险评估报告是实验室采取风险管理措施、建立安全管理体系文件、制定安全操作规程等过程中的依据，并可考核。

5.5 再评估

实验室应根据活动的进程或风险特征的变化适时启动风险再评估工作。正常情况下，实验活动进行中每年应对风险评估报告进行一次再评估(或称复评审)，以便持续识别新的风险或发生的风险改变。

再评估的要求和程序与初次进行风险评估时相同。但根据病原体特性、实验活动类型、设施设备和人员等评估对象的变更情况不同可以适当简化，有所侧重。

实验室出现变化时，应重新进行风险评估或对风险评估报告进行再评估。这些变化包括但不限于：

——致病性生物因子的生物学特性发生改变时；

——实验室运行相关的关键设施或设备发生变化时；

——人员，尤其机构法人代表、项目负责人等关键岗位人员发生变化时；

——实验活动内容，包括实验方法、操作程序、实验动物种类等发生改变时；

——较大幅度增加病原操作量时，包括操作样品数量、单个样品的体积等；

——实验室自身发生事件、事故，或实验室工作与自身实验室类似的国内外相关实验室发生重大事故时；

——相关法律、法规或标准发生变化，或者行业主管部门发布新的相关管理通知或公告时；

——对该致病性生物因子引起的疾病防控策略发生变化时；

——管理层从风险控制的需要，认为应该再评估时。

附　录　A
（资料性附录）
实验室生物安全风险评估的常用方法

实验室生物安全风险评估常用方法参见表 A.1。

表 A.1　实验室生物安全风险评估的常用方法

序号	风险评估技术	说　　明	适用阶段或范围
1	头脑风暴法及结构化访谈	一种收集各种观点及评价并将其在团队内进行评级的方法。头脑风暴法可由提示、一对一以及一对多的访谈技术所激发	风险评估的各阶段
2	德尔菲法	一种综合各类专家观点并促其一致的方法，这些观点有利于支持风险源及影响的识别、可能性与后果分析以及风险评价，需要独立分析和专家投票	风险评估的各阶段
3	情景分析	在想象和推测的基础上，对可能发生的未来情景加以描述。可以通过正式或非正式的、定性或定量的手段进行情景分析	风险评估的各阶段
4	检查表	一种简单的风险识别技术，提供了一系列典型的需要考虑的不确定性因素。使用者可参照以前的风险清单、规定或标准	风险评估的各阶段
5	预先危险分析（Primary Hazard Analysis，简称 PHA）	一种简单的归纳分析方法。其目标是识别风险以及可能危害特定活动、设备或系统的危险性情况及事项	多用于病原微生物实验室设计和建设的初期，适用于风险识别的各阶段
6	失效模式和效应分析（Failure Mode and Effect Analysis，简称 FMEA）	一种识别失效模式、机制及其影响的技术。多用于实体系统中的组件故障	多用于实验室操作活动、单一设备、简单系统的风险评估
7	危险与可操作性分析（Hazard and Operability studies，HAZOP）	一种综合性的风险识别过程，用于明确可能偏离预期绩效的偏差，并可评估偏离的危害度。它使用一种基于引导词的系统	适用于实验室设施设备的风险评估，适用于风险识别的各阶段
8	危险分析与关键控制点（Hazard Analysis and Critical Control Points，简称 HACCP）	一种系统的、前瞻性及预防性的技术，通过测量并监控那些应处于规定限值内的具体特征来确保产品质量、可靠性以及过程的安全性	适用于实验室设施设备的风险评估，适用于风险识别的各阶段

表 A.1（续）

序号	风险评估技术	说　　明	适用阶段或范围
9	结构化假设分析（Structure“What if”，简称 SWIFT）	一种激发团队识别风险的技术，通常在引导式研讨班上使用，并可用于风险分析及评价	适用于风险识别的各阶段
10	风险矩阵	一种将后果分级与风险可能性相结合的方式	适用于风险识别的各阶段
11	人因可靠性分析	主要关注系统绩效中人为因素的作用，可用于评价人为错误对系统的影响	多用于生物因子、实验活动等风险评估，适用于风险识别的各阶段
12	以可靠性为中心的维修	一种基于可靠性分析方法实现维修策略优化的技术，其目标是在满足安全性、环境技术要求和使用工作要求的同时，获得产品的最小维修资源消耗	多用于简单设备风险评估，适用于风险识别的各阶段
13	压力测试	在极端情境下（最不利的情形下），评估系统运行的有效性，发现问题，制定改进措施的方法	多用于实验室测试验证阶段，适用于风险识别的各阶段
14	保护层分析法	也被称作障碍分析，它可以对控制及其效果进行评价	多用于实验室设计和建设初期，适用于风险识别的各阶段
15	故障树分析	始于不良事项的分析并确定该事件可能发生的所有方式，并以逻辑树形图的形式进行展示。在建立起故障树后，就应考虑如何减轻或消除潜在的风险源。适用于风险评估的各阶段	适用于风险识别的各阶段
16	事件树分析	运用归纳推理方法将各类初始事件的可能性转化成可能发生的结果。除风险评价外，适用于风险评估的其他阶段	适用于风险识别的各阶段
17	因果分析	综合运用故障树分析和事件树分析，并允许时间延误。初始事件的原因和后果都要予以考虑。适用于风险评估的各阶段	适用于风险识别的各阶段

附　录　B
（资料性附录）
实验室生物安全风险评估矩阵

B.1　事件发生可能性的确定参见表 B.1。

表 B.1　事件发生的可能性

级别	说　明	描　　述
Ⅰ	基本不可能发生	评估范围内未发生过，类似区域/行业也极少发生
Ⅱ	较不可能发生	评估范围内未发生过，类似区域/行业偶有发生
Ⅲ	可能发生	评估范围内发生过，类似区域/行业也偶有发生；评估范围未发生过，但类似区域/行业发生频率较高
Ⅳ	很可能发生	评估范围内发生频率较高
Ⅴ	肯定发生	评估范围内发生频率极高

B.2　后果严重性评估参见表 B.2。

表 B.2　事件导致后果的严重性

级别	说　明	描　　述
1	影响很小	基本没有影响，不会造成不良的社会影响
2	影响一般	发生病原微生物泄漏，现场处理（第一时间救助）可以立刻缓解事故，中度财产损失，有较小的社会影响
3	影响较大	发生病原微生物泄漏、实验室人员感染，需要外部援救才能缓解，引起较大财产损失或赔偿支付，在一定范围内造成不良的影响
4	影响重大	发生病原微生物泄漏、实验室外少量人员感染，造成严重财产损失，造成恶劣的社会影响
5	影响特别重大	病原微生物外泄至周围环境，造成大量社会人员感染伤亡、巨大财产损失，造成极其恶劣的社会影响
注 1：该表用于设施设备风险评估时，是指因设施设备故障或功能缺陷（如：生物安全柜过滤器泄漏，高压灭菌器压力表指示正常但压力达不到相应要求）而导致病原微生物没有得到有效控制，而造成的泄露、感染或其他损失。<br>注 2：后果导致的严重性分级时，可以与 GB 19489 或 GB 50346 实验室分级依据相结合考虑。		

B.3 根据事件发生的可能性和后果严重性的组合,可将风险等级划分为低、中、高、极高四个级别。可按照表B.3确定。

表B.3 风险等级矩阵

		后果严重性				
		1	2	3	4	5
事件发生的可能性	Ⅰ	低	低	低	中	中
	Ⅱ	低	低	中	中	高
	Ⅲ	低	中	中	高	高
	Ⅳ	中	中	高	高	极高
	Ⅴ	中	高	高	极高	极高
图例:□低风险 □中风险 □高风险 □极高风险						

附　录　C
（资料性附录）
病原微生物实验活动风险评估实施参考示例

C.1　任务来源

说明该项工作任务由哪个部门下达，有明确的任务来源和要求。

C.2　评估目的

说明此时开展该项评估工作的目的，如：为实验室初次认可评估、变更活动范围评估，或者是按照实验室制定的年度计划对实验室正在使用的风险评估报告进行再评估或定期复评审。

C.3　评估准备

接收到评估任务后，实验室指定风险评估负责人，成立风险评估小组（或其他称谓），进行工作分工，界定人员、职责和权限，制定风险评估实施方案。在收集信息资料、初步分析风险预期基础上，明确内外部环境信息，制定风险准则。

C.4　评估实施

C.4.1　沟通、交流与咨询

就实验室拟开展的病原微生物实验活动与利益相关方进行充分的沟通交流，并就关键环节和不明确要素咨询有关专家。

C.4.2　风险识别

对拟开展的实验活动进行确定，并对实验活动中涉及的风险（包括生物安全风险和生物安保风险）逐一识别，形成风险列表。比如：对拟开展高致病性禽流感病毒实验活动的动物生物安全三级实验室，实验活动包括：疑似高致病性禽流感病毒感染样品的接收、PCR或荧光PCR检测，通过鸡胚接种分离培养禽流感病毒。根据主管部门规定，该实验室对分离到的高致病性禽流感病毒不进行保存，直接送参考实验室保存。因此，该实验室涉及的生物因子状况和风险列表应包括但不限于：

——生物因子特性描述：

高致病性禽流感病毒的生物学特性、传播途径，易感动物、易感人群，抵抗力，引起的疾病，发病率、死亡率，公共卫生影响等。

——实验室周边环境状况描述：

描述实验室周边的环境，包括是否有家禽养殖场，与养殖场、居民区的距离，是否有野禽、飞鸟栖息或寄居等，以便分析病原从实验室泄露后的影响。

——实验室活动中的风险：

a）样品的接收与处理：

1）样品接收过程中可能的风险，如：样品包装出现泄漏；

2） 样本处理过程中可能的风险，如：打开包装、转移样品、匀浆、研磨、离心等。

b） 检测过程：

1） 检测过程中的风险，如：阳性样品存放遗失，因标识不清与其他样品混合不清等；

2） 设备使用中的风险，如：匀浆器、研磨器、离心机等设备的使用；

3） 分离培养的病毒培养液运输至参考实验室可能的风险，如：包装泄漏，路上遗失或被盗走；

4） 其他。

c） 阳性样品或分离培养的病毒的转运、运输和保存：

1） 实验室内的转运；

2） 向参考实验室的运送；

3） 在本实验室内的暂时保存；

4） 其他。

d） 适用时，实验室本身或者相关实验室已发生的事故分析。

——非常规实验室活动风险：

a） 非实验室人员进入实验室监督、审核或者学习交流等；

b） 运行维护人员进入实验室进行设施设备维修更换等；

c） 其他。

——设施设备风险：

a） 围护结构、暖通空调、电气自控、给水排水等；

b） 安全防护设备，如生物安全柜、高压灭菌器等；

c） 研究检测设备，如核酸提取仪、酶标仪等。

——实验动物：

本案例涉及孵化的鸡胚，不涉及其他实验动物。

——人员：

a） 人员身体状况和心理状况；

b） 人员能力；

c） 各种压力对人员影响；

d） 团队状况等。

——生物安保：

a） 被误用和恶意使用；

b） 样本被偷盗；

c） 阳性培养物或样本运输至参考实验室；

d） 其他。

——涉及的化学、物理、辐射、电气、水灾、火灾、自然灾害等风险。

C.4.3 风险分析、评价与应对

考虑拟操作病原微生物的特性，针对 C.4.2 识别出来的风险列表，根据风险准则进行风险分析，做出风险评价，制定出相应的风险应对措施。

进行风险分析、评价，制定应对措施时，可以采取段落叙述方式，也可以采取列表形式。采用段落叙述时，宜清楚描述风险分析、风险评价和风险应对的全部过程。

——风险分析

根据风险识别出的不同风险要素，对风险涉及事件在本实验室发生的可能性及其后果的严重性进行充分分析，并据此确定风险等级，可以描述为低、中、高或极高等四个级别。具体可参照附录 B 中的

相关表格内容确定。

——风险评价

实验室可以根据上述每个识别出的风险的分析结果，对照风险准则内容，根据拟采取的应对措施状况以及自身实际情况，作出风险是否可接受的判定。

——风险应对

根据风险评价结果，当风险可接受时，可以通过制定相应的预防控制措施或保持已有的安全措施，防止事故的发生；当风险不可接受时，实验室可以根据风险不可接受的程度和风险的特征采取相应的风险应对措施，以便消除、降低或控制风险。

本附录采用列表方式对风险评估要素进行说明，在风险评估基础上制定管理措施、设施设备控制措施和个人防护装备措施。示例参见表C.1。

表 C.1　某动物生物安全三级实验室病原微生物实验活动风险评估与应对措施表（示例）

风险评估			风险应对			
风险识别	风险分析	风险评价	管理要求	管理措施	设施设备控制	个人防护装备控制
1.健康风险						
(1)是否对人或者动物有害	具体分析人或动物感染后的状况，发生的频率及其后果严重程度。描述发生事件的可能性和后果严重性，并根据事件发生的可能性和后果的严重性，对风险等级进行综合判定，确定低、中、高或极高级别	对照风险准则，确定风险是否可接受	根据能否导致人或/和动物感染，提出针对性的管理要求	制定相应的标准操作规范（SOP），SOP 要覆盖实验室生物安全和安保的全部范围	要求相对隔离，强调与其它实验活动的分开，实验室设施的安全保障	针对是否能对人的感染，提出穿戴个人防护装备的措施要求
(2)样品的接收与处理环节存在风险	分析样品的来源、种类，通过了解样品背景评估样品为阳性样品的几率，包装破损等可能的风险。分析样品接收和处理过程中在该实验室内发生风险的可能性以及一旦发生泄露后的影响程度，根据表 B.3 风险等级矩阵，对样品接收处理过程中发生病原泄露事件的风险作出等级判定	根据实验室对样品接收和处理环节可能面临风险的状况分析结果，对照准则，作出是否可以接受风险的判定	提出管理要求，如细化制定样品接收 SOP，明确风险关键环节，强调对样品接收人员进行上岗培训等	根据管理要求提出可行的管理措施。例如，由经过授权的人员承担样品接收任务；要求接样人员在样品接收时填写样品接收单，并在样品传递至核心工作间时方可打开包装、核对样品数量和状态	提出设施设备控制的要求，如进行验证样品质量时，须将盛放样品的箱子转移至实验室核心工作间才能打开外包装。必要时，要求在生物安全柜内打开包装	根据对人或动物的危害程度、操作样品的数量（样品数、样品体积）等因素，对需要穿戴的个人防护用品（PPE）做出规定。例如：经提前与送样方沟通，了解得知样品箱内含有较大液体量包装时，为防止喷溅要求戴防喷溅头套，或者护目镜

表 C.1（续）

风险评估			风险应对			
风险识别	风险分析	风险评价	管理要求	管理措施	设施设备控制	个人防护装备控制
(3)实验室内开展工作中的风险	对实验室内开展的活动进行分析,例如:疑似高致病性禽流感病毒感染样品经 PCR 检测阳性后,尚需要进行鸡胚接种培养活动,进一步还需要进行静脉接种致病指数(IVPI)测定病毒毒力。这些实验操作产生意外风险的可能性。根据存在泄露可能性以及泄漏后后果的严重程度分析结果,作出等级评价	根据实验室内开展工作的管理状况和风险分析结果,对照预先制定的判定准则,作出风险是否可以接受的判定	实验室内工作的相应要求,例如:制定发现阳性病例报告的计划	制定针对风险点控制的管理措施,例如:规定发现阳性样品后的措施;实验过程中动物组织、用过的滴头、注射器等所有潜在污染的废弃物处理措施	需要的设施设备控制要求,例如:鸡胚接种要求在生物安全柜内操作;鸡只静脉注射时在禽隔离器内进行;废弃物处理需要高压灭菌器。生物安全柜、高压灭菌器应经过检定、验证符合要求等	进行人兽共患病原的实验室内操作,应使用 N95 口罩、头罩和连体防护服等措施
2.环境风险						
病原环境中比较稳定(如:非洲猪瘟病毒、炭疽芽孢杆菌),或者环境中相对不稳定[如:艾滋病病毒(human immunodeficiency virus,HIV)]	分析病毒在外界的存活能力,对酸碱、温度等的抵抗力。例如:禽流感病毒在粪便中可存活 1 周等。结合病原对环境污染的可能性大小和一旦污染后的严重程度,做出等级评定	根据实验室的设施设备条件、环境条件,配备的高压灭菌器、消毒剂等资源,确定是否可以接受	提出灭活该种病毒的措施要求	针对不同实验活动环节,制定消毒、去污染的措施。包括:使用的消毒剂或灭菌方法,浓度、作用时间等	不适用	明确使用消毒剂应进行的个体防护措施和个人防护装备(PPE),包括防止病毒或细菌对操作人员的暴露需要穿戴的 PPE、防止消毒剂对操作人员的化学伤害等

表 C.1（续）

风险评估			风险应对			
风险识别	风险分析	风险评价	管理要求	管理措施	设施设备控制	个人防护装备控制
3.设施设备风险						
(1)通风空调系统	分析通风空调系统发生故障的表现形式、风险因素,例如:排风高效过滤器泄漏(可能因为未及时检修和更换等)。对发生故障的可能性、发生后的严重程度分别进行分析,并综合判定风险等级,例如:影响大、特别巨大等	根据通风空调故障的具体表现和实验室应对措施、资源,作出是否可接受的判定	提出对通风空调系统的管理要求	针对通风空调系统可能出现的问题,例如:未设置备用送风机、未设置备用排风机、气流组织不符合要求、生物密闭阀密封措施失效等,制定具体的管理措施	配备相应的检测设备、监测设备,或与第三方检测机构合作进行必要的设备配置	提出维修、去污染过程中需要穿戴的个人防护装备
(2)生物安全柜	分析生物安全柜可能出现的故障风险,比如:表现为排风高效过滤器泄漏、工作窗口气流反向、工作窗口风速偏低。对这些故障发生的可能性和发生后后果的严重程度进行判定。通过对生物安全柜发生某类故障的可能性,发生这种故障后给实验室生物安全带来的危害严重程度进行比较分析,做出风险等级的评价	根据安全柜可能发生的故障类型及风险分析结果,作出是否可接受的判断	将生物安全柜检定列入年度安全计划。通过安全检查、内部审核等措施,及时识别生物安全柜故障。例如:制定生物安全柜定期检定计划	实施实验室设施设备维护和期间核查方案。比如:按照实验室制定的设备检定计划安排,按时进行高效过滤器检修、更换;对安全柜排风、风速等性能,委托专业单位进行检修	更换生物安全柜高效过滤器期间,避免未经防护的人员接近	提出维修、期间核查过程中,工作人员需要穿戴PPE操作,尤其进行高效过滤器更换时

表 C.1（续）

风险评估			风险应对			
风险识别	风险分析	风险评价	管理要求	管理措施	设施设备控制	个人防护装备控制
(3)高压灭菌器	分析高压灭菌器可能的故障来源和表现形式,比如:消毒灭菌效果验证不合格、压力表/压力传感器失真、温度表/温度传感器失真等,并对自身实验室管理状态下发生故障的可能性以及后果严重性进行分析。结合故障发生的可能性和故障发生带来的消毒不彻底后果严重程度进行风险等级评价	根据高压灭菌器可能发生的故障类型及风险分析结果,作出是否可接受的判断,以便后续采取应对措施	将高压灭菌器检定列入年度安全计划。通过安全检查、内部审核、期间核查等措施,及时识别高压灭菌器故障	安排专人按时对高压灭菌器消毒灭菌效果进行验证,委托计量检定机构按时对压力表/压力传感器、温度表/温度传感器进行检定,确保符合工作要求	对设备使用进行管理控制。例如:对设备进行标识。在没有经过检定和灭菌效果验证前,不得用于废弃物品的高压灭菌消毒	使用过程中做好个人防护。比如:某些情况下操作高压灭菌器时,需要配戴棉手套操作,防止烫伤
4.安保风险						
(1)短期保存阳性样品<br>(2)短期保存接种疑似禽流感病毒阳性的鸡胚尿囊液,或者接种了非洲猪瘟病毒的细胞培养液	分析实验室采取的安保措施能否满足要求,可能导致的被盗、丢失、误用的可能性,以及发生该类事件后的影响。针对实验室短期保存阳性样品和阳性培养物(如:鸡胚尿囊液、非洲猪瘟病毒等病原)发生误用、被盗的可能性和严重程度,做出风险等级评价	根据实验室采取的安保措施以及所保存的对象危害程度等的综合分析结果,作出是否可以接受的判定	提出安全保障政策程序,例如:要求对来访人员进行登记管理;阳性病毒培养尿囊液、病毒细胞培养液采用双人双锁管理	制定管理措施,例如:(1)对来访人员进行事先登记管理,发放来宾识别卡。(2)在来宾参观实验室过程中有实验室专人陪同,避免来访人员与阳性样品、接种样品收获的尿囊液、病毒细胞培养液直接接触。(3)病毒培养阳性的尿囊液或细胞培养液采取双人双锁管理,使用前须经过审批	对设施设备进行检查和完善。如:(1)在存放阳性样品或病毒培养液的冰箱或冰柜加锁管理。(2)调整摄像头,对存放区域实施无死角的 24 小时实时监控	不适用

表 C.1（续）

风险评估			风险应对			
风险识别	风险分析	风险评价	管理要求	管理措施	设施设备控制	个人防护装备控制
5.经济风险(适用时)						
事件发生后引起经济损失	说明是否会引起疾病的暴发或大流行，发生后导致的因人的感染或者动物死亡、贸易受影响等的经济损失。例如：因暴发高致病性禽流感，禁止禽类产品出口或跨区域调运；再比如，暴发非洲猪瘟后禁止生猪和猪产品运出，分析对贸易影响，综合判定评估范围内发生事件后的经济风险等级	根据实验室对风险识别和采取措施的状况、风险分析结果，作出是否可接受的判定	提出相应的病原操作管理要求，必要时建议政府出台管理规定	兽医或卫生主管部门要求病原分离培养必须严格在 BSL-3/ABSL-3 或 BSL-4/ABSL-4 实验室条件下进行	按照实验室规定，进行疑似病原感染样品操作和病毒/细菌培养必须在 BSL-3/ABSL-3 或者 BSL-4/ABSL-4 实验室进行，对废弃物、剩余的菌毒液等要求经过高压灭菌处理后方可出实验室	不适用

注 1：本附录仅为实验室开展包含样品检测在内的实验活动风险评估列举的示例，不代表病原微生物实验室生物安全风险评估的全部。各级各类实验室应根据自身实验室状况逐一识别出潜在的生物安全风险，并就风险进行评估分析和评价，提出有效的应对和控制措施。

注 2：对高致病性禽流感、非洲猪瘟、口蹄疫等可引起动物大量发病、疫情大流行，并因动物大量死亡、贸易限制等因素带来巨大经济损失的病原微生物，在进行风险评估时应涵盖病原体实验室泄露后的经济风险评估。

C.4.4　风险交流

风险评估完成后，针对风险评估结果与相关部门和人员等利益相关方就实验室活动、可能的风险、实验室拟采取的生物安全保障措施等内容进行充分交流。

与相关方进行交流时，应明确(但不限于)：

——本实验室涉及的可能生物危害，可能操作的病原，例如：非洲猪瘟病毒、高致病性禽流感病毒或炭疽芽孢杆菌等；

——实验室开展的工作对相关方的意义；

——开展工作前对可能涉及的风险进行了风险评估；

——根据风险评估结果，实验室制定了相应的生物安全措施及安保措施；

——实验室的各项生物安全管理职责得到了充分的落实；

——对可能存在的不足和发现的问题制定了纠正措施，并可以持续的改进和完善。

C.5　记录开展风险评估的全过程

对开展风险评估过程中的相关信息按照5.4.5.1的要求进行详细记录并归档保存，以便于持续改进和追溯。

参 考 文 献

［1］ ISO 31000:2018 Risk management—Guidelines
［2］ GB/T 27921—2011 风险管理 风险评估技术
［3］ GB/T 24353—2009 风险管理 原则与实施指南
［4］ GB 50346—2011 生物安全实验室建筑技术规范
［5］ RB/T 199—2015 实验室设备生物安全性能评价技术规范
［6］ 病原微生物实验室生物安全管理条例(国务院令第424号)
［7］ Manual of Diagnostic Tests and Vaccines for Terrestrial Animals. 8th Edition，2018. https://www.oie.int

RB/T 040—2020

中 华 人 民 共 和 国 认 证 认 可
行 业 标 准
病原微生物实验室生物安全风险管理指南
RB/T 040—2020
*
中 国 标 准 出 版 社 出 版 发 行
北京市朝阳区和平里西街甲 2 号(100029)
北京市西城区三里河北街 16 号(100045)
网址:www.spc.org.cn
服务热线:400-168-0010
2020 年 11 月第一版
*
书号:155066・2-35697

版权专有　侵权必究

RB/T 040-2020

码上扫一扫　正版服务到
