# Supplementary material for: Biosafety status analysis and risk assessment of laboratories from 2021 to 2023 in Jiaxing, China
Source: Front Bioeng Biotechnol. 2025 Apr 16;13:1442651. doi: 10.3389/fbioe.2025.1442651 (PMC12040964; doi:10.3389/fbioe.2025.1442651)
Supplement: Supplementary file 1 [file DataSheet1.zip › supplementary/ref 23.浙江省卫生健康委办公室关于印发浙江省病原微生物实验室生物安全应急预案的通知.pdf]

浙江省卫生健康委员会办公室文件

浙卫办〔2022〕33号

浙江省卫生健康委办公室关于印发浙江省病原微生物实验室生物安全应急预案的通知

各市卫生健康委，省级医疗卫生单位：

现将《浙江省病原微生物实验室生物安全应急预案》印发给你们，请结合当地和单位实际，认真贯彻执行。

浙江省卫生健康委办公室

2022年10月18日

浙江省病原微生物实验室生物安全应急预案

一、总则

（一）编制目的。

为加强我省病原微生物实验室生物安全管理工作，有效预防和控制病原微生物实验室生物安全事件，保障人民群众身体健康和生命安全，维护社会稳定。

（二）编制依据。

《中华人民共和国生物安全法》《中华人民共和国传染病防治法》《中华人民共和国突发事件应对法》《中华人民共和国国家安全法》《突发公共卫生事件应急条例》《病原微生物实验室生物安全管理条例》《浙江省病原微生物实验室生物安全管理办法（试行）》等。

（三）工作原则。

1.统一领导，分级管理。在浙江省卫生健康委统一领导和指挥下，根据实验室生物安全事件的范围、性质和危害程度实行分类分级管理和处置，按照“谁主办，谁负责；谁主管，谁负责；谁使用，谁负责”的原则，各有关部门和单位在各自职责范围内履行实验室生物安全意外事件应急处置的职责。

2.预防为主，科学防范。在日常工作中落实好预防病原微生物实验室生物安全事件发生的各项措施，做好风险排查、物资储

备和应急演练，提高防范意识，及时消除安全隐患。

3.依法管理，联防联控。遵循相关法律、法规，建立联防联控屏障，统一调控，协同联动，信息共享，加强信息报告，力争早发现、早报告、早控制。

（四）适用范围。

本预案适用于浙江省范围内发生的人间传染的病原微生物实验室生物安全相关事件的应急处置工作。

二、事件分级

实验室生物安全事件是指病原微生物菌（毒）种或生物样本在采集、运输、使用、保存（保藏）、销毁，以及研究、教学、检测、诊断等活动过程中，因自然灾害、操作不规范造成人员感染或暴露，或丢失、被盗、被抢等意外事件。

根据实验室生物安全事件的性质、危害程度和涉及范围，将实验室生物安全事件划分为特别重大、重大、较大、一般四个级别。

（一）特别重大实验室生物安全事件。

1.实验室工作人员确诊或疑似感染所从事实验活动涉及的以下病原微生物，且引起重症感染或者人员死亡的;

（1）高致病性病原微生物;

（2）我国尚未发现或已经宣布消灭的病原微生物;

（3）未列入《人间传染的病原微生物名录》的高致病性病

原微生物或疑似高致病性病原微生物；

2.从事前款所述高致病性病原微生物实验活动造成实验室相关工作人员感染且引起实验室以外人员感染的；

3.发生腺鼠疫、埃博拉等重大传染病的病原微生物菌（毒）种或生物样本被盗、被抢、丢失等事件；

4.省级卫生健康行政部门认定的其它特别重大实验室生物安全事件。

（二）重大实验室生物安全事件。

1.实验室工作人员确诊或疑似感染所从事实验活动涉及的高致病性病原微生物；

2.实验室工作人员感染所从事实验活动涉及的《人间传染的病原微生物名录》中非高致病性病原微生物，引起重症感染或人员死亡的；

3.其它高致病性病原微生物菌（毒）种或生物样本丢失、被盗、被抢；

4.省级卫生健康行政部门认定的其它重大实验室生物安全事件。

（三）较大实验室生物安全事件。

1.实验室工作人员确诊或疑似感染所从事实验活动涉及的《人间传染的病原微生物名录》中非高致病性病原微生物；

2.实验室工作人员从事高致病性病原微生物实验活动过程

中发生职业暴露，且需要预防或者阻断措施的；

3.实验室发生高致病性病原微生物菌（毒）种或生物样本溢洒、泄露；

4.实验室发生非高致病性病原微生物菌（毒）种或生物样本丢失、被盗、被抢等事件；

5.省级卫生健康行政部门认定的其它较大实验室生物安全事件。

（四）一般实验室生物安全事件。

1.实验室发生《人间传染的病原微生物名录》中非高致病性病原微生物菌（毒）种或生物样本溢洒、泄露；

2.实验室工作人员从事《人间传染的病原微生物名录》中非高致病性病原微生物实活动过程中发生职业暴露，且需要预防或者阻断措施的；

3.省级卫生健康行政部门认定的其它一般实验室生物安全事件。

三、组织机构与职责

（一）省级组织机构与职责。

1.领导机构。浙江省病原微生物实验室生物安全领导小组（以下简称领导小组）是全省实验室生物安全事件应急处置的指挥机构，统一领导和指挥全省实验室生物安全事件的应急处置工作。领导小组成员单位按照职责范围规定开展有关应急处置工作。

2.日常办事机构。浙江省病原微生物实验室生物安全领导小组办公室（以下简称办公室）设在省卫生健康委科教处。办公室负责组建省级实验室生物安全应急处置专家组，下设三个专业组（包括处置组、检测组、医疗救治组），各专业组按照工作职责组织开展实验室生物安全事件处置，并及时向领导小组报告。办公室负责做好日常管理及相关信息的收集、汇总和分析。

浙江省病原微生物实验室生物安全领导协调小组办公室负责应急演练方案的制定和组织实施。

（1）处置组。省卫生健康委疾病预防控制与职业健康处、浙江省疾病预防控制中心负责开展突发事件现场流行病学调查，对应急处置工作中涉及场所封闭、病人隔离治疗、现场消毒、密切接触者医学观察等提供专家咨询和技术指导。对突发事件相关信息进行分析研判和风险评估，提出应对措施意见建议。承担办公室交办的其他工作。

（2）检测组。浙江省疾病预防控制中心负责检测组的组建、人员召集和相关准备和任务的实施。具体负责病原分离、检测、鉴定，以及事件发生所在实验室微生物相关数据收集、分析，查找事件原因，为制定现场处置和抢救方案提供依据。

（3）医疗救治组。浙江大学医学院附属第一医院负责医疗救治组的组建、人员召集和相关准备及任务的实施。具体负责组织开展实验室生物安全事件感染者和医学观察人员的临床救治

及业务指导。

（二）市级组织机构与职责。

各市应成立相应的生物安全应急处置工作领导小组，按照属地化管理的原则，负责辖区内的实验室生物安全事件处置的管理与指导、协调。

各市要制定当地实验室生物安全意外事件应急处置预案，成立处置、检测、医疗救治工作组，并明确职责和工作流程，落实各项防控措施，责任到人。当发生特别重大及重大生物安全事件时，在迅速采取措施控制事件发展的同时应立即向省级卫生健康行政部门报告。

各市应指定专门的医疗机构负责较大和一般病原微生物实验室生物安全事件感染者的收治和医学观察人员的隔离观察，必要时报由省办公室统一协调解决。

（三）县（市、区）级组织机构与职责。

各县（市、区）应成立相应的生物安全应急处置工作领导小组，按照属地化管理的原则，在市级主管部门指导下负责辖区内的实验室生物安全事件处置管理。

各县（市、区）要制定当地实验室生物安全意外事件应急处置预案，必要时成立应急处置工作组，并明确职责和工作流程，落实各项防控措施，责任到人。当发生特别重大及重大生物安全事件时，在迅速采取措施控制事件发展的同时应立即向省、市级

卫生健康行政部门报告。

四、监测

（一）监测网络。

各级卫生健康行政部门及实验室设立单位要建立完善实验室生物安全事件监测网络，开展实验室工作人员健康监测、症状监测、舆情监测等。

（二）日常监测。

做好实验室生物安全事件日常监测工作，定期收集相关信息资料，认真核实分析。

（三）评估。

根据各类监测数据，组织专家开展日常风险评估和专题风险评估，及时发现事件风险隐患。

（四）动态调整。

充分利用数字化、人工智能等先进技术提升监测的效率和质量，动态调整监测方式和策略。

五、报告

（一）报告要求。

任何单位和个人都有权向各级卫生健康行政部门报告实验室生物安全事件或重大安全隐患，也有权向上级卫生健康行政部门举报不履行或不按规定履行实验室生物安全事件应急处置职责的部门、单位及个人。

任何单位和个人对实验室生物安全事件不得瞒报、迟报、谎报或授意他人瞒报、迟报、谎报，不得阻碍他人报告。

（二）责任报告单位。

各实验室设立单位为实验室生物安全事件的责任报告单位。

（三）报告时限要求。

1.发生特别重大、重大、较大实验室生物安全事件时，责任报告单位应在 2 小时内向所在地县（市、区）级卫生健康行政部门报告。接到信息报告的卫生健康行政部门应当立即组织进行现场调查确认，初步判断事件级别，并将初步判断结果立即向上级卫生健康行政部门和同级人民政府报告，同时组织现场处置组、专家组赶赴现场，开展现场调查评估、医学救援、隔离观察、洗消防护等应急处置工作。如发生高致病性病原微生物菌（毒）种及生物样本被盗、被抢、丢失还应向所在地县（市、区）公安部门报告。

2.特别重大和重大实验室生物安全事件还应向办公室报告，如已造成突发公共卫生事件，应按事件级别启动应急响应，并按《浙江省突发公共卫生事件应急预案》要求由县（市、区）卫生健康行政部门在 2 小时内上报省委、省政府及国家卫生健康委。

3.一般实验室生物安全事件由实验室设立单位负责处置，结束后应将事件发生及处置情况书面报所在地卫生健康行政部门。

4.相关卫生健康行政部门应视情况及时互相通报信息。

（四）初次报告。

报告内容包括实验室设立单位名称、实验室名称、事件发生地点、发生日期和时间、涉及病原体名称、涉及的地域范围、感染或暴露人数、发病人数、死亡人数、密切接触者人数、发病者主要症状与体征、原因、已采取的措施、初步判定的事件级别、事件的发展趋势、下一步应对措施、报告单位、报告人员及通讯方式等。初次报告强调及时性，暂时未获得的信息可在进程报告和结案报告中补充完善。

（五）进程报告。

报告事件的发展与变化、处置进程、势态评估、控制措施等内容。同时，对初次报告内容进行补充和修正。

特别重大和重大实验室生物安全事件每日进行进程报告。

（六）结案报告。

事件处置结束后，应进行结案信息报告。在卫生健康行政部门确认事件终止后 2 周内，对事件的发生和处理情况进行总结，分析其原因和影响因素，并提出今后对类似事件的防范和处置建议。

六、应急处置

（一）I级响应。

发生特别重大实验室生物安全事件后，经省级卫生健康行政部门评估确定启动I级响应，并采取以下措施。

1.现场调查与处置。

（1）实验室设立单位应立即启动本单位应急预案，关闭发生事件的实验室；对周围环境进行隔离、封闭和现场消毒，防止污染扩大；并在2小时内向所在地县（市、区）卫生健康行政部门报告。

核实在相应潜伏期时间段内进入实验室人员及密切接触感染者人员的名单，并进行医学观察、必要时进行隔离；被感染人员尽快安全转运至定点救治医院；配合卫生健康行政部门做好感染者救治及现场调查和处置工作，提供实验室布局、设施、设备、实验人员等信息资料。

发生腺鼠疫、埃博拉等重大传染病的病原微生物菌（毒）种或生物样本被盗、被抢、丢失等事件时，应配合当地公安部门开展调查处置。

（2）市级卫生健康行政部门应组织应急处置人员组成现场处置组，封闭现场；了解核实事件信息，初步认定事件等级；进行现场采样、流行病学调查；对现场采取必要隔离、封闭、消毒措施；对感染人员及疑似感染人员进行隔离、救治；对在相应潜伏期时间段内进入实验室人员及密切接触感染者的人员进行隔离医学观察。立即上报省级卫生健康行政部门和本级人民政府并协助控制事件发展。必要时要做好心理疏导工作。县（市、区）卫生健康行政部门协助做好有关应急处置工作。

（3）省级卫生健康行政部门接到报告后应立即报省委省政府相关部门，并组织专家赶赴现场进行指导，对事件发生原因及存在的生物安全隐患进行分析，认定事件等级，提出指导和评估意见，制定防控和医疗救治方案；及时向省委省政府相关部门提交相关处置进展报告。

2.事件结束。

组织省级专家组进行评估，确认受污染区域得到有效消毒、生物安全事件造成的感染者已妥善治疗和安置、在最长的潜伏期内未出现新的感染者后，由省级卫生健康行政部门宣布应急处置工作结束。省级卫生健康行政部门将事件发生及处理情况书面报送省委省政府及国家卫生健康委。

3.信息发布。

事件信息由省级卫生健康行政部门负责沟通及对外发布（包括上级部门、相关委办厅（局）和新闻媒体等）。

（二）II级响应。

发生重大实验室生物安全事件后，经省级卫生健康行政部门评估确定启动II级响应，并采取以下措施。

1.现场处置与调查。

（1）实验室设立单位应立即启动本单位应急预案，关闭发生事件的实验室；对周围环境进行隔离、封闭和现场消毒，防止污染扩大；并在2小时内向所在地县（市、区）卫生健康行政部

门报告。

核实在相应潜伏期时间段内进入实验室人员及密切接触感染者人员的名单，并进行医学观察、必要时进行隔离；被感染人员尽快安全转运至定点救治医院；配合卫生健康行政部门做好感染者救治及现场调查和处置工作，提供实验室布局、设施、设备、实验人员等信息资料。

发生其它高致病性病原微生物菌（毒）种或生物样本丢失、被盗、被抢等事件时，应配合当地公安部门开展调查处置。

（2）市级卫生健康行政部门应组织应急处置人员组成现场处置组，封闭现场；了解核实事件信息，初步认定事件等级；进行现场采样、流行病学调查；对现场采取必要隔离、封闭、消毒措施；对感染人员及疑似感染人员进行隔离、救治；对在相应潜伏期时间段内进入实验室人员及密切接触感染者的人员进行隔离医学观察。立即上报省级卫生健康行政部门和本级人民政府并协助控制事件发展。必要时要做好心理疏导工作。县（市、区）卫生健康行政部门协助做好有关应急处置工作。

（3）省级卫生健康行政部门接到报告后应立即报省委省政府相关部门，并组织专家赶赴现场进行指导，对事件发生原因及存在的生物安全隐患进行分析，认定事件等级，提出指导和评估意见，制定防控和医疗救治方案；及时向省委省政府相关部门提交相关处置进展报告。

2.事件结束。

组织省级专家组进行评估，确认受污染区域得到有效消毒、生物安全事件造成的感染者已妥善治疗和安置、在最长的潜伏期内未出现新的感染者后，由省级卫生健康行政部门宣布应急处置工作结束。省级卫生健康行政部门将事件发生及处理情况书面报送省委省政府及国家卫生健康委。

（三）III级响应。

发生较大实验室生物安全事件后，经市级卫生健康行政部门评估确定启动III级响应，并采取以下措施。

1.实验室设立单位应立即启动本单位应急预案；在2小时内向所在县（市、区）卫生健康行政部门报告；按照本单位应急处置预案进行现场处置；做好感染人员的救治工作；做好实验室及外环境的有效消毒。

2.市级卫生健康行政部门应组织应急处置人员组成现场处置组，进行现场调查确认，初步认定事件级别，立即上报上级卫生健康行政部门；指导实验室设立单位做好实验室感染人员治疗、相关人员的追踪；经市级专家组评估确认后宣布应急处突工作结束，将事件发生及处理情况书面报送上级卫生健康行政部门。县（市、区）卫生健康行政部门协助做好有关应急处置工作。

（四）IV级响应。

发生一般实验室生物安全事件后，经实验室设立单位评估确定

启动Ⅳ级响应，并采取以下措施。

实验室设立单位应立即启动本单位应急预案；对实验室内环境做好有效消毒；对暴露人员做好评估工作；指导实验室人员进行预防用药或阻断措施，及时做好相关记录。事件结束后，将事件发生及处理情况书面报送所在地的县（市、区）卫生健康行政部门。

（五）实验室涉及生物恐怖的事件应急程序与处置。

实验室涉恐事件是指在病原微生物实验室发生的可能涉及生物恐怖袭击的事件，包括破坏实验室设施、病原微生物菌（毒）种库或其信息系统；抢夺、盗窃高致病性病原微生物菌（毒）种或样本及其他感染性材料；在实验室内故意播撒高致病性病原微生物菌（毒）种或样本等事件。实验室设立单位发现各种实验室涉恐事件要立即向当地公安机关、市级卫生健康行政部门报告，启动本单位应急预案。市级卫生健康行政部门向省级卫生健康行政部门报告，省级卫生健康行政部门接到报告后应立即向省反恐怖工作协调小组报告。

七、预警

办公室组织专家做好可能引发实验室生物安全事件的信息分析，提出预警建议。各级卫生健康行政部门应及时发布预警。预警信息应包括事件类别、可能波及范围、可能危害程度、可能延续时间、提醒事宜和应采取的相应防控措施等，并随着事态的

发展和变化进行调整。根据其可能造成的危害程度、紧急程度和发展态势，预警级别分别用红色（特别重大）、橙色（重大）、黄色（较大）、蓝色（一般）来展示。事件得到有效控制后，应及时解除预警。

八、善后处理

（一）职责分配。

善后处理工作由事发地有关主管部门负责，省级有关部门提供必要的支持。造成实验室生物安全事件的责任单位和责任人应当按照有关要求组织开展整改，消除安全隐患。

（二）监督整改。

事发地卫生健康行政部门根据应急处置工作情况，对实验室生物安全事件发生单位、责任单位的整改工作进行监督，及时跟踪处理情况，通报处理结果。

（三）责任追究。

对在事件的预防、监测、报告、调查、控制和处置过程中，存在玩忽职守、失职、渎职等行为，以及不遵守有关规定、不配合或拒不执行应急管控措施的，依据相关法律法规追究责任。

（四）后期评估。

实验室生物安全事件善后处理工作结束后，各级卫生健康行政部门要会同实验室设立单位的主管部门，对实验室生物安全事件发生的起因、性质、影响、后果、责任和现场处置能力等问题，

进行调查评估，总结经验教训，提出改进建议，完成应急处置总结报告报送办公室。

九、应急保障

（一）组织保障。

1.各级政府加强对事件应急处置工作的统一领导、统一指挥;明确各部门职责任务,建立事件防范和应急处置工作责任制,保证事件应急处置工作科学有序、依法规范。

2.应急预案启动后，属地公安部门应配合专业主管部门做好实验室生物安全事件污染区域的外围及周边地区的治安维护工作。

（二）人员保障。

1.各级卫生健康行政部门应根据应急处置需要组建实验室生物安全事件处置组和专家组,各实验室设立单位要明确本单位生物安全事件应急处置部门及人员，明确责任，具体到人，措施到位,保持通讯畅通。定期组织开展应急演练。在重大活动期间,应做好值班值守工作。

2.实验室设立单位根据实验室生物安全事件应急预案，做好应急处置的各项技术保障的准备，制定切实可行的工作方案，并通过举办培训或演练使相关人员熟悉掌握应急处置的基本流程和处置要求，以提高应急处置能力。

（三）经费和物资保障。

1.各级政府按照我省医疗卫生领域财政事权和支出责任划分改革有关规定，落实事件应急准备、处置和常态化建设经费。财政部门安排事件应急预算并及时拨付资金，督促相关部门做好经费的绩效评价工作。

2.各级卫生健康行政部门应当储备必要的现场应急处置个人防护、现场隔离、转运和洗消、废物处置和抢险救援等物资；做好医疗人员、床位、救治设备和应急药品、车辆、疫苗等调配工作；备齐必要的采样、检验、存储、鉴定和检测设备。

3.实验室设立单位应根据本单位从事的实验活动，明确定点救治医疗机构，建立快速对接通道，储备足够的应急处置个人防护装备，包括防护服、防护面屏、呼吸防护装置、手套及鞋（靴）套；储备足量且有效的消毒灭菌设备及试剂；应急药品和急救器材等。

附件：1. 病原微生物实验室生物安全事件报告流程图

2. 浙江省实验室生物安全事件报告表

附件 1

病原微生物实验室生物安全事件报告流程图

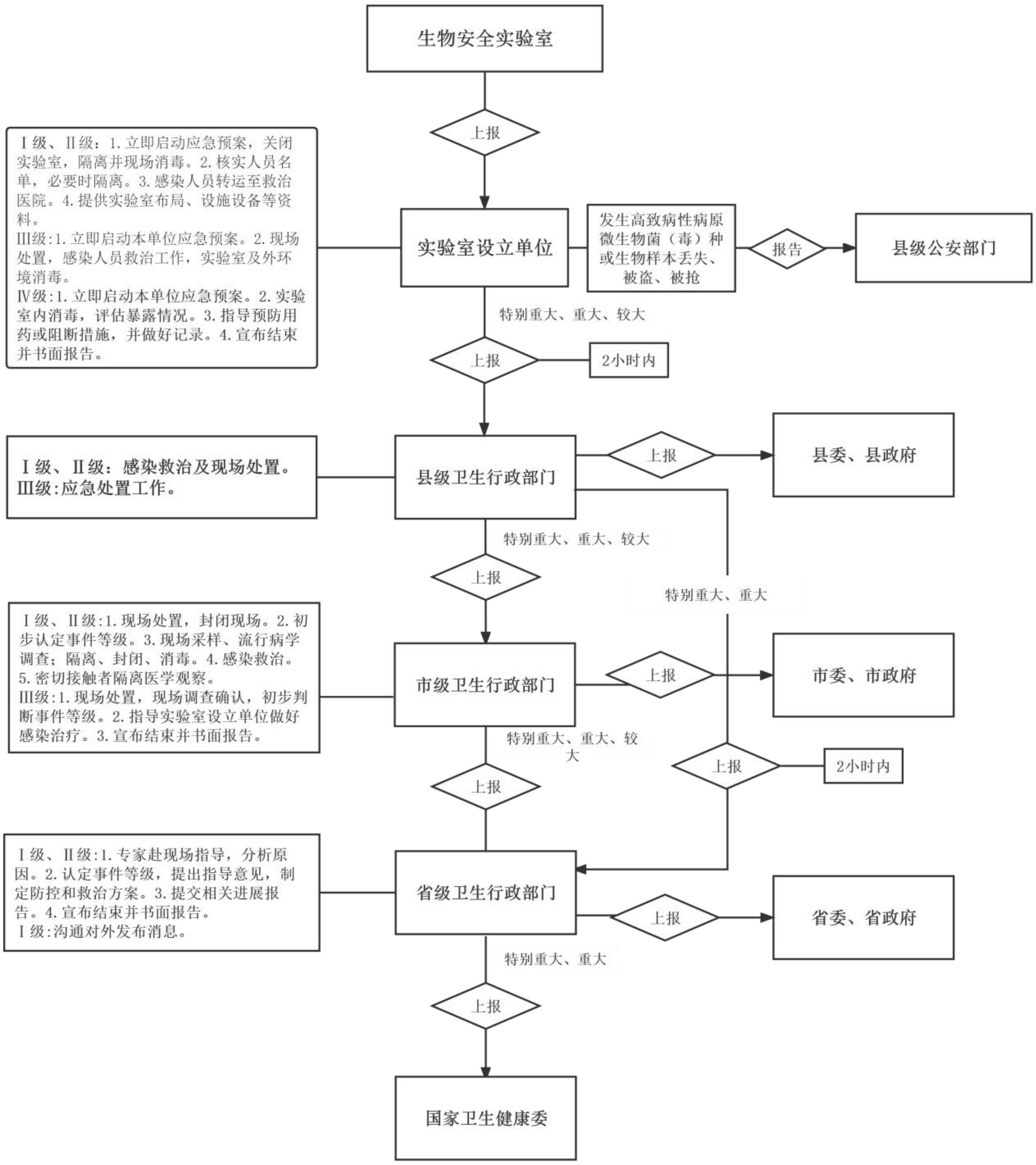

附件 2

浙江省实验室生物安全事件报告表

单位名称（盖章）:　　　　　　　　单位生物安全负责人:

实验室负责人:　　　　　　　　　　联系电话:

报告类别: □初次报告 □阶段报告 □总结报告

<table>
<tr><td>事件发生日期</td><td></td><td>事件发生时间</td><td></td></tr>
<tr><td>发生实验室名称</td><td></td><td>事件发生具体位置</td><td></td></tr>
<tr><td>所涉及病原微生物名称</td><td></td><td>所涉及病原微生物危害等级</td><td></td></tr>
<tr><td>实验活动类型</td><td></td><td>当事人</td><td></td></tr>
<tr><td>事件级别</td><td colspan="3">□特别重大实验室生物安全事件
□重大实验室生物安全事件
□较大实验室生物安全事件
□一般实验室生物安全事件
□待认定</td></tr>
<tr><td>发生环节</td><td colspan="3">□采集 □运输 □实验活动 □销毁 □保存（保藏）</td></tr>
<tr><td>事件类别</td><td colspan="3">□洒溢 □职业暴露 □丢失 □被盗、被抢 □人员感染 □人员死亡
□其它:</td></tr>
</table>

事件发生过程	
事件处理过程	
事件处理结果	
向何部门/人员报告	
报告方式	
报告人	
报告日期及时间	

填 表 人:　　　　　　　　　　　　填表时间:

浙江省卫生健康委员会办公室　　　　　　2022年10月18日印发

（校对：朱颖辉）
